# Supplementary material for: Inhibitory CARs fail to protect from immediate T cell cytotoxicity
Source: Mol Ther. 2024 Feb 22;32(4):982–99. doi: 10.1016/j.ymthe.2024.02.022 (PMC11163222; doi:10.1016/j.ymthe.2024.02.022)
Supplement: Document S1. Figures S1–S14 [file mmc1.pdf]

YMTHE, Volume *32*

Supplemental Information

Inhibitory CARs fail to protect from immediate T cell cytotoxicity

Maximilian A. Funk, Gerwin Heller, Petra Waidhofer-Söllner, Judith Leitner, and Peter Steinberger

Supplemental Figures

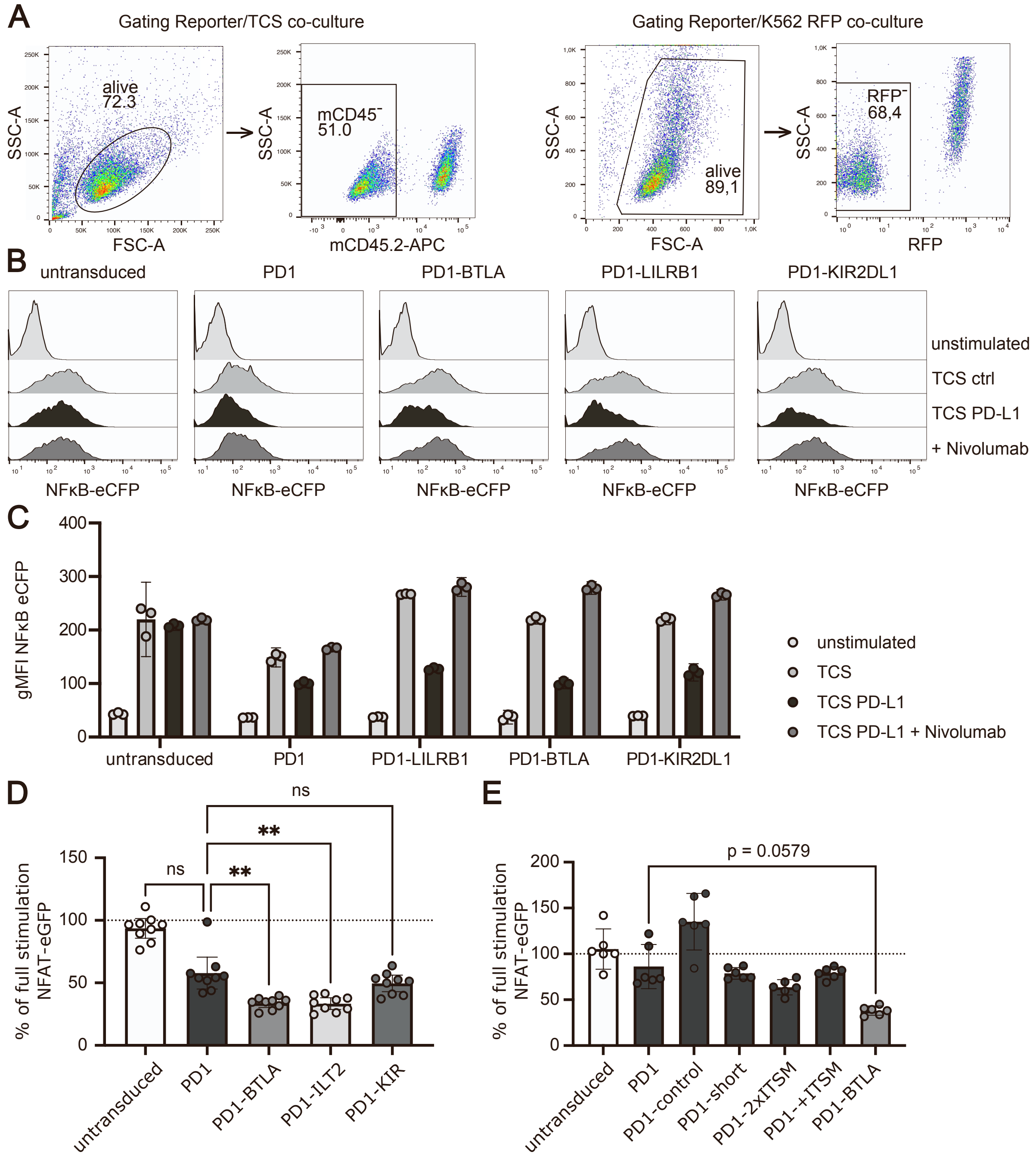

Fig. S1 **Supplemental information to Figure 1. A** Gating strategy to identify TPR cells in coculture assays with TCS cells that express murine CD45 (left) and K562 RFP (right). **B** Representative histograms showing induction of NFκB-eCFP reporter gene expression under the indicated stimulation conditions. **C** Representative assay showing reduced expression of the NFκB-eCFP reporter gene by TPR expressing PD1 or PD1-chimera if stimulated by TCS PD-L1 compared to stimulation with TCS. The effect is reversible by adding the PD1 blocking antibody Nivolumab (n=1, assay performed in triplicates). Data is presented as triplicate mean with 95% CI. Dots indicate individual repeats. **D** Analysis of inhibition of the NFAT-eGFP reporter gene in coculture assay described in Fig. 1D. **E** Analysis of inhibition of the NFAT-eGFP reporter gene in coculture assay described in Fig. 1G. Kruskal-Wallis test with Dunn's multiple comparisons test was used to compare all groups to inhibition by PD1. (** $p<0.01$; ns, not significant)

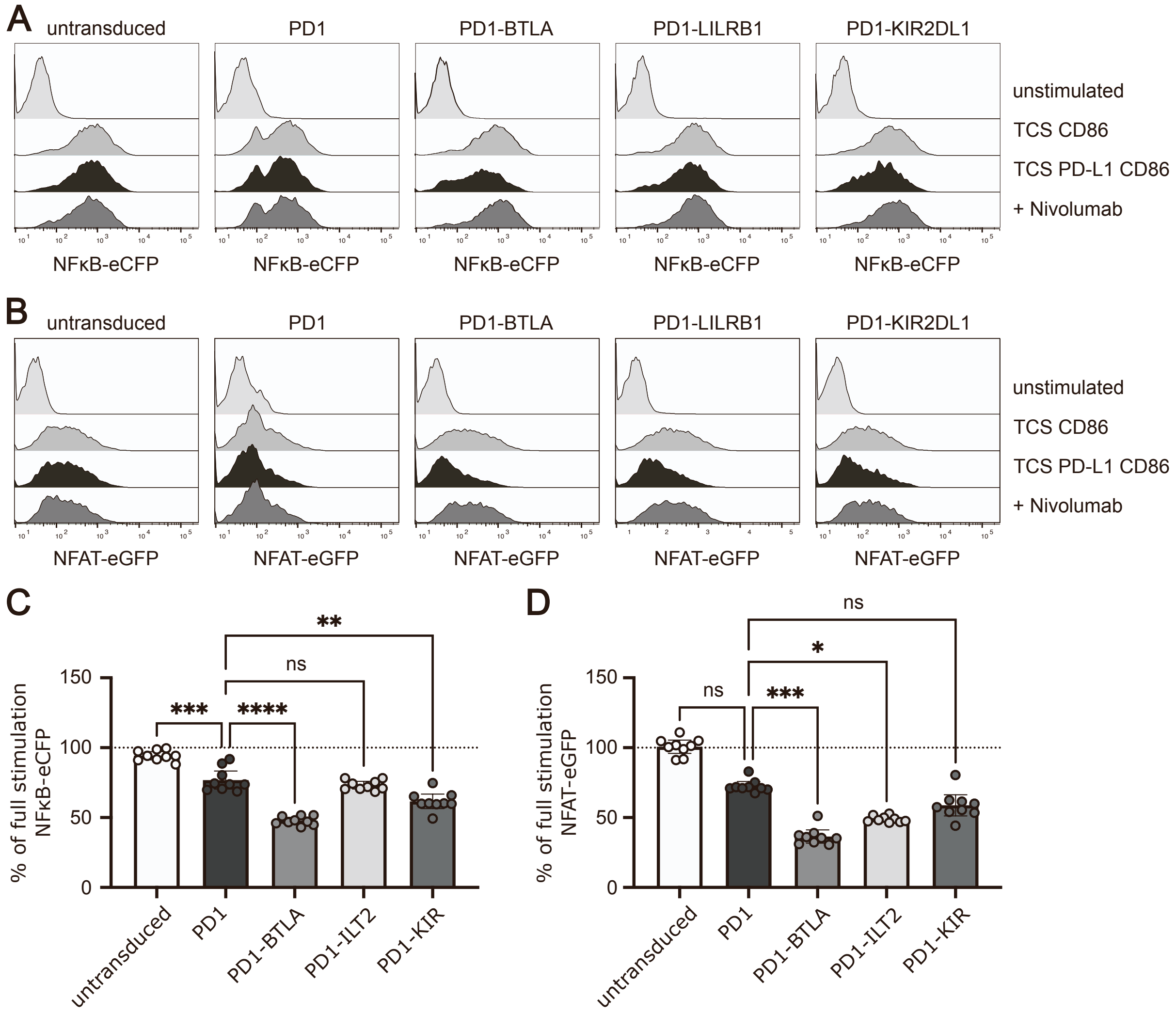

Fig. S2 **Influence of inhibitory signaling on costimulation via the CD28/CD86-pathway** TPR expressing the indicated PD1-chimera were cocultured with TCS CD86 or TCS PD-L1 CD86. After 24h, reporter gene expression was assessed by flow cytometry. **A,B** Representative histograms showing reporter gene fluorescence intensity of the indicated cell lines under different coculture conditions. (**A** NFκB-eCFP, **B** NFAT-eGFP). **C,D** Analysis of pooled data (n=3, each assay in triplicates). Stimulation in presence of TCS PD-L1 CD86 is expressed as percentage of stimulation by TCS CD86. Data is presented as mean with 95% CI. Dots represent individual repeats. **C** Percentage of full stimulation of the NFκB-eCFP reporter gene. Brown-Forsythe and Welch ANOVA tests with Dunnett's T3 multiple comparisons test was used to compare all groups to inhibition by PD1. **D** Percentage of full stimulation of the NFκB-eCFP reporter gene. Kruskal-Wallis test with Dunn's multiple comparisons test was used to compare all groups to inhibition by PD1. (* $p<0.05$; ** $p<0.01$; *** $p<0.001$; **** $p<0.0001$; ns, not significant)

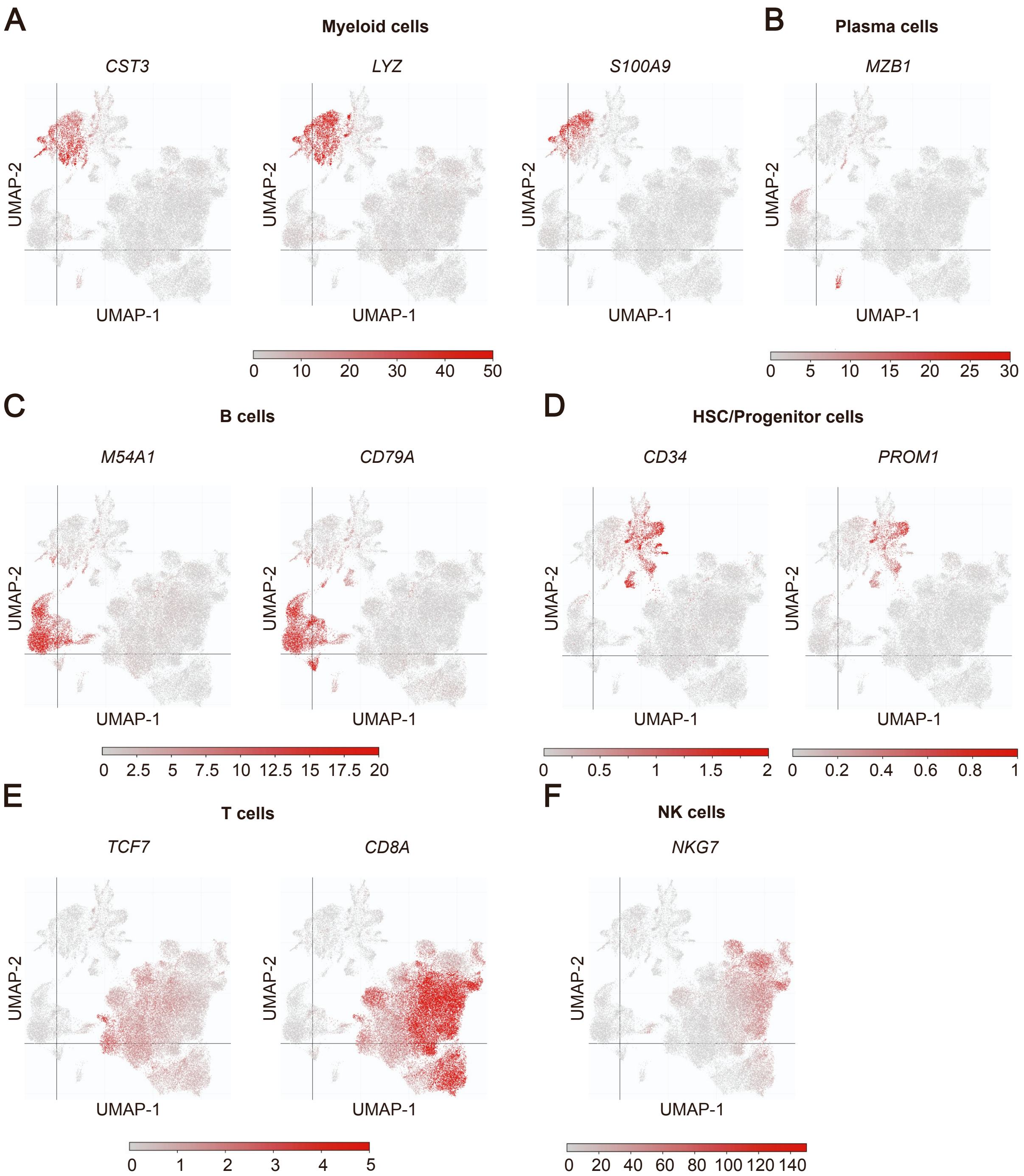

Fig. S3 **Identification of cell types in healthy control bone marrow samples.**
Publicly available, single cell level transcriptional data of bone marrow samples from healthy donors (n=10) was reanalyzed for cell type specific gene expression. UMAP projections of these cells are shown and expression of specific marker genes is highlighted in red **A** Myeloid cells were defined by high expression of *CST3*, *LYZ* and *S100A9,* **B** Plasma cells were defined by high expression of *MZB1,* **C** B cells were defined by high expression of *M54A1* and *CD79A,* **D** HSC/Progenitor cells were defined by high expression of *CD34* and *PROM1,* **E** T cells were defined by high expression of *TCF7* and *CD8A* **F** NK cells were defined by high expression of *NKG7.*

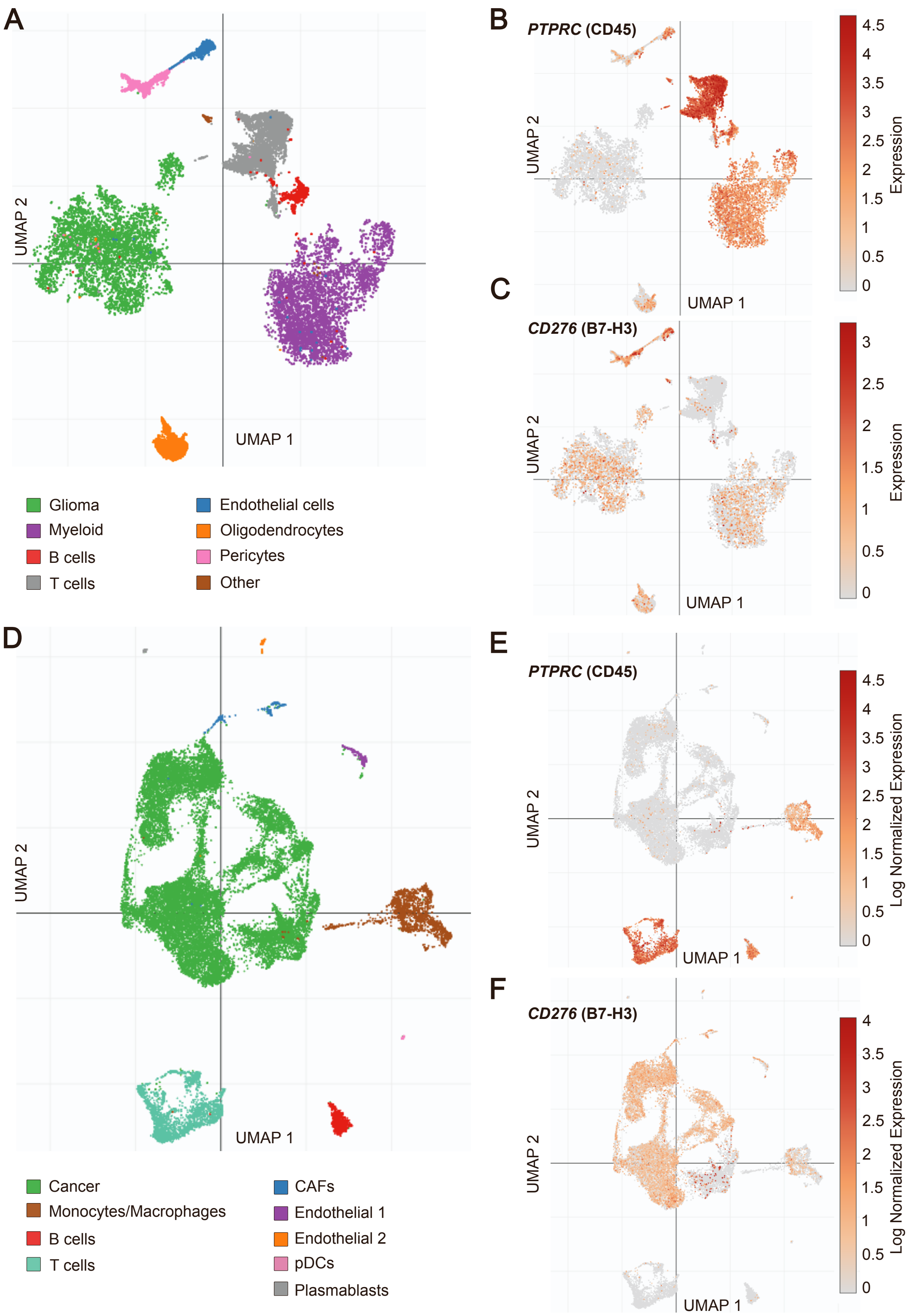
A
UMAP 2
UMAP 1
Glioma
Myeloid
B cells
T cells
Endothelial cells
Oligodendrocytes
Pericytes
Other
B
PTPRC (CD45)
UMAP 2
UMAP 1
4.5
4
3.5
3
2.5
2
1.5
1
0.5
0
Expression
C
CD276 (B7-H3)
UMAP 2
UMAP 1
3
2.5
2
1.5
1
0.5
0
Expression
D
UMAP 2
UMAP 1
Cancer
Monocytes/Macrophages
B cells
T cells
CAFs
Endothelial 1
Endothelial 2
pDCs
Plasmablasts
E
PTPRC (CD45)
UMAP 2
UMAP 1
4.5
4
3.5
3
2.5
2
1.5
1
0.5
0
Log Normalized Expression
F
CD276 (B7-H3)
UMAP 2
UMAP 1
4
3.5
3
2.5
2
1.5
1
0.5
0
Log Normalized Expression

Fig. S4 **Expression pattern of *PTPRC* (CD45) and *CD276* (B7-H3) in primary human tumor samples.** Publicly available, single cell level transcriptional data of glioma patients (n=18, 201.986 cells total) and a melanoma patient (n=1, 21,363 cells) were analyzed using the Single Cell Portal online-tool. **A** UMAP projection showing cell clustering and cell type annotation in glioma samples as defined in the metadata to this dataset (subsampling of 20.000 cells).[64] **B/C** UMAP projections indicating expression pattern of *PTPRC* (CD45) (**B**) and *CD276* (B7-H3) (**C**) **D** UMAP projection showing the cell type annotation in a melanoma patient as defined in the metadata to this dataset.[65] **E/F** UMAP projections indicating expression pattern of *PTPRC* (CD45) (**E**) and *CD276* (B7-H3) (**F**)

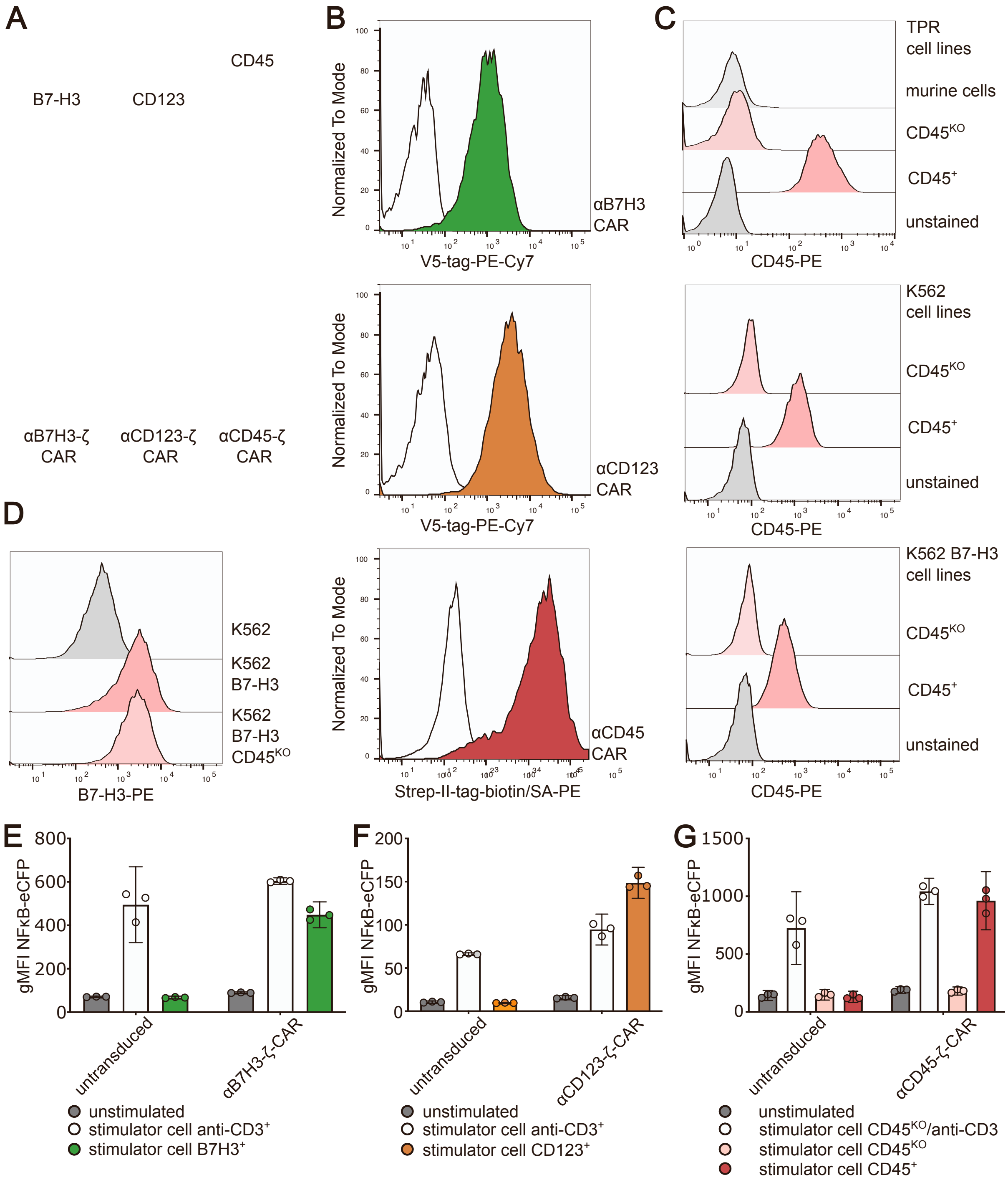

Fig. S5 **Creation of functional CARs from antibody sequences.**

A Scheme depicting assay setup to test functionality of the αB7H3-, αCD123- and αCD45-CARs. **B** Expression of the indicated CAR molecules on TPR cells determined by flow cytometry. Untransduced TPR cells served as negative control (open histograms) **C** To avoid interference with naturally expressed CD45 on TPR and K562 cells, CRISPR/Cas9 mediated knock out of endogenous CD45 was performed. Histograms show expression of CD45 on the treated (light red) and untreated (dark red) cell lines (TPR, K562, K562 B7-H3). Unstained cells (dark grey) and murine cells (light grey) not expressing human CD45 served as negative controls. **D** Expression of B7-H3 on K562 target cell lines analyzed by flow cytometry. **E/F/G** Untransduced TPR or TPR transduced with αB7H3- ζ (**E**), αCD123- ζ (**F**) or αCD45-ζ (**G**) CARs were cocultured with the, indicated K562 stimulator cells (n=1 each, each assay performed in triplicates). Data is presented as individual values with mean and 95% CI.

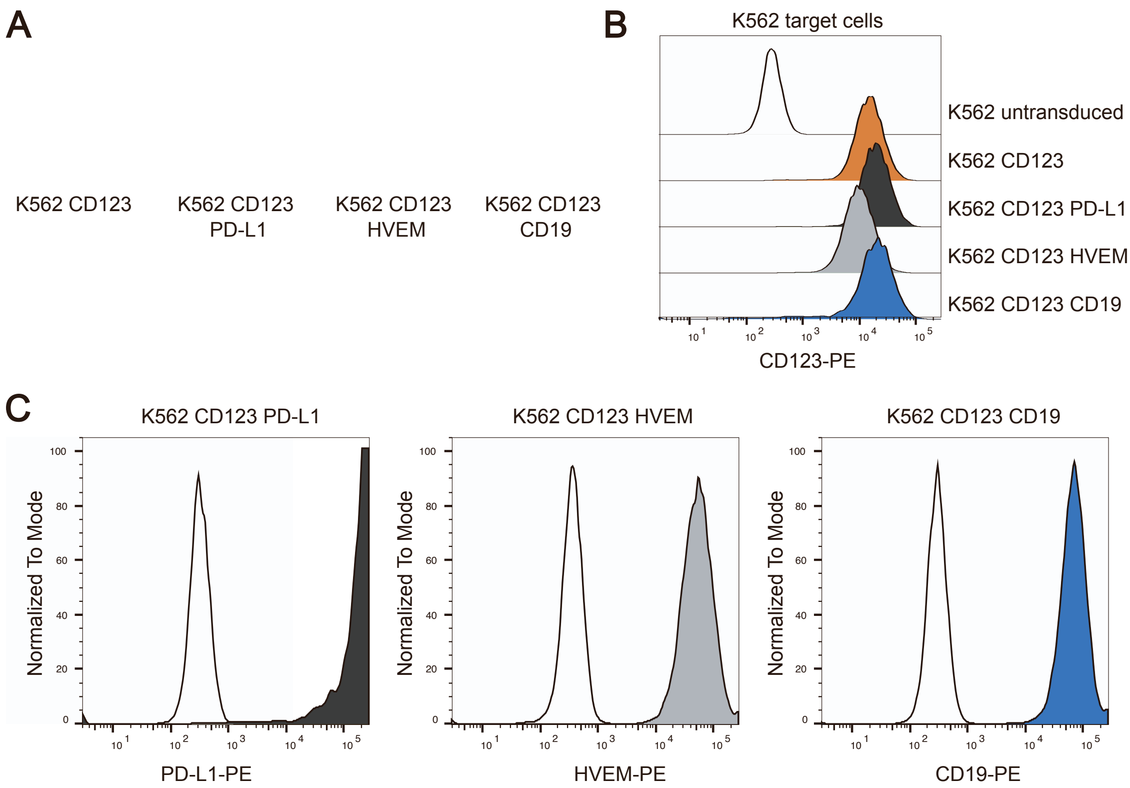

Fig. S6 **Creation and profiling of CD123 expressing K562 target cells. A** Scheme of K562 target cells that expressed CD123 alone or together with PD-L1, HVEM or CD19. **B** Expression of CD123 on all target K562 target cell lines as assessed by flow cytometry. Untransduced K562 cells served as negative control. **C** Panels show expression of inhibitory ligands PD-L1 (left, dark grey), HVEM (middle, light grey) and CD19 (right, blue) on the respective target cells as assessed by flow cytometry. Untransduced K562 served as negative controls.

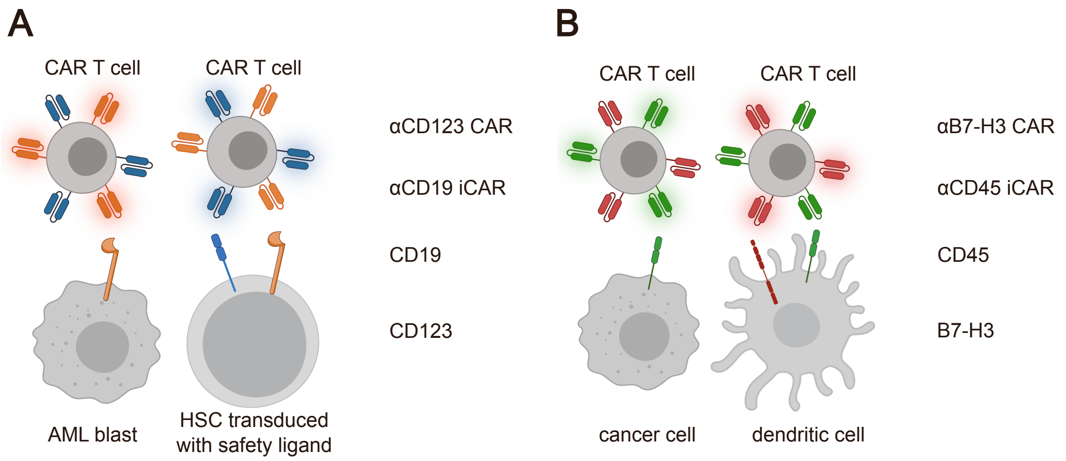

Fig. S7 **Potential CAR/iCAR antigen combinations to address relevant on-target/off-tumor toxicities A** Scheme outlining the αCD123-CAR/αCD19-iCAR strategy. CD123 is expressed on both, AML and HSCs. Transduced of HSCs expressing CD19 as “safety ligand” could avoid CAR/iCAR-T cell mediated toxicity. **B** Scheme outlining the αB7H3-CAR/αCD45-iCAR strategy. B7-H3 is expressed on many cancer entities and certain hematopoietic cell types, including dendritic cells. An αCD45-iCAR could prevent CAR/iCAR-T cell activation against hematopoietic cells.

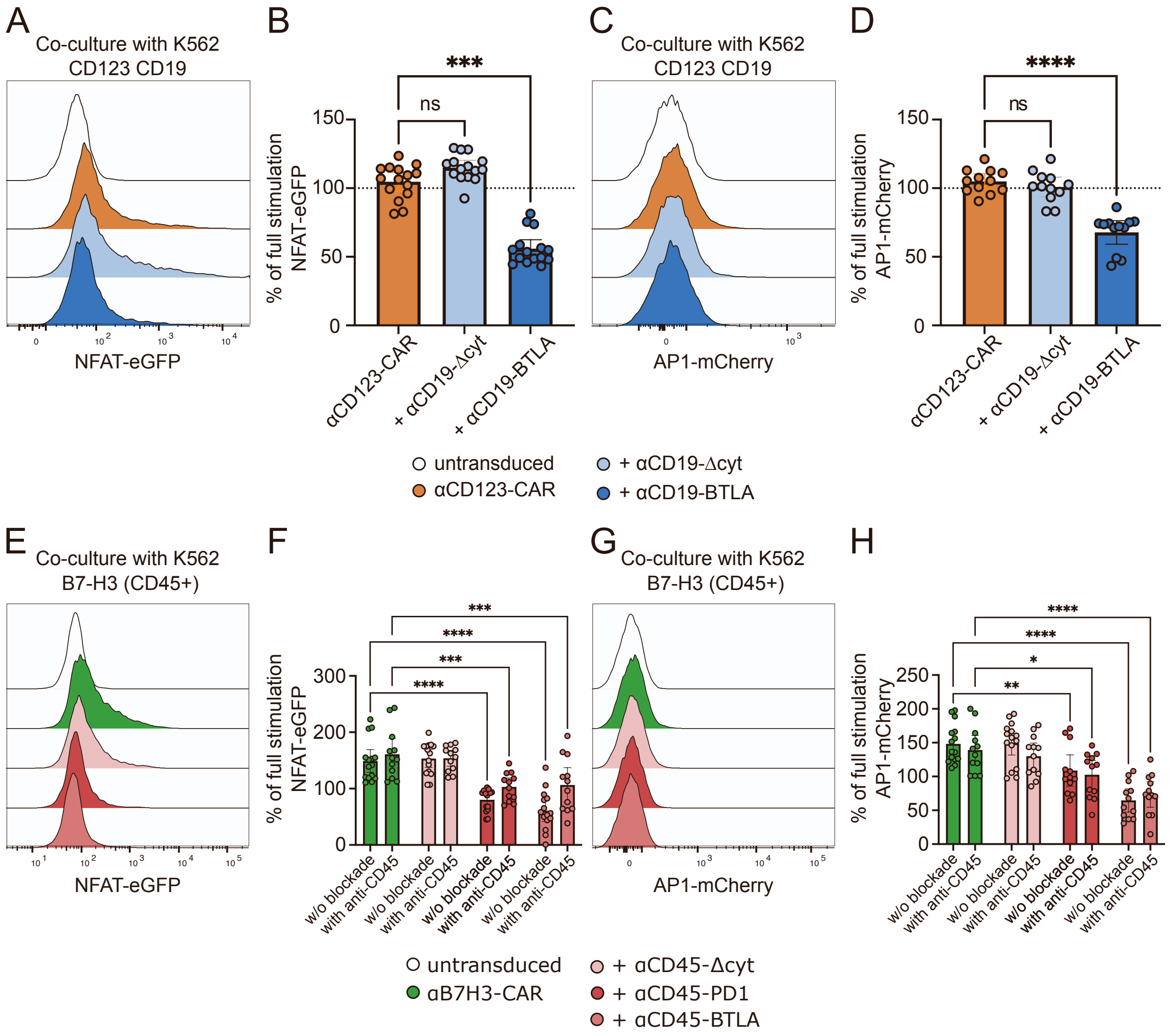

Fig. S8 **Effect of iCARs on NFAT-eGFP and AP1-mCherry reporter gene transcription A-D** Data on NFAT-eGFP and AP1-mCherry reporter gene transcription corresponding to the experiment shown in Fig. 3B,C. **A** Representative histograms showing NFAT-eGFP fluorescence intensity of the indicated TPR cell lines in coculture with K562 CD123 CD19. **B** Pooled data (n=5, each assay in triplicates) presented as mean with 95% CI. Dots represent individual repeats. **C,D** Data on AP1-mCherry reporter gene presented analogous to **A,B**, respectively. **B,D** Kruskal-Wallis test with Dunn's multiple comparisons test was performed to compare groups with αCD19-iCARs to stimulation by αCD123-ζ CAR alone. **E-H** Data on NFAT-eGFP and AP1-mCherry reporter gene transcription corresponding to the experiment shown in Fig. 3E,F. **E** Representative histograms showing NFAT-eGFP fluorescence intensity of the indicated TPR cell lines in coculture with K562 B7-H3 (CD45+). **F** Pooled data (n=5/ n=4 with anti-CD45-antibody, each assay in triplicates) presented as mean with 95% CI. Dots represent individual repeats. **G,H** Data on AP1-mCherry reporter gene presented analogous to **E,F**, respectively. **F,H** 2way-ANOVA with Dunnett's multiple comparisons test was performed to compare all groups to stimulation by αB7H3-ζ CAR alone. (* $p<0.05$; ** $p<0.01$; *** $p<0.001$; **** $p<0.0001$; ns, not significant)

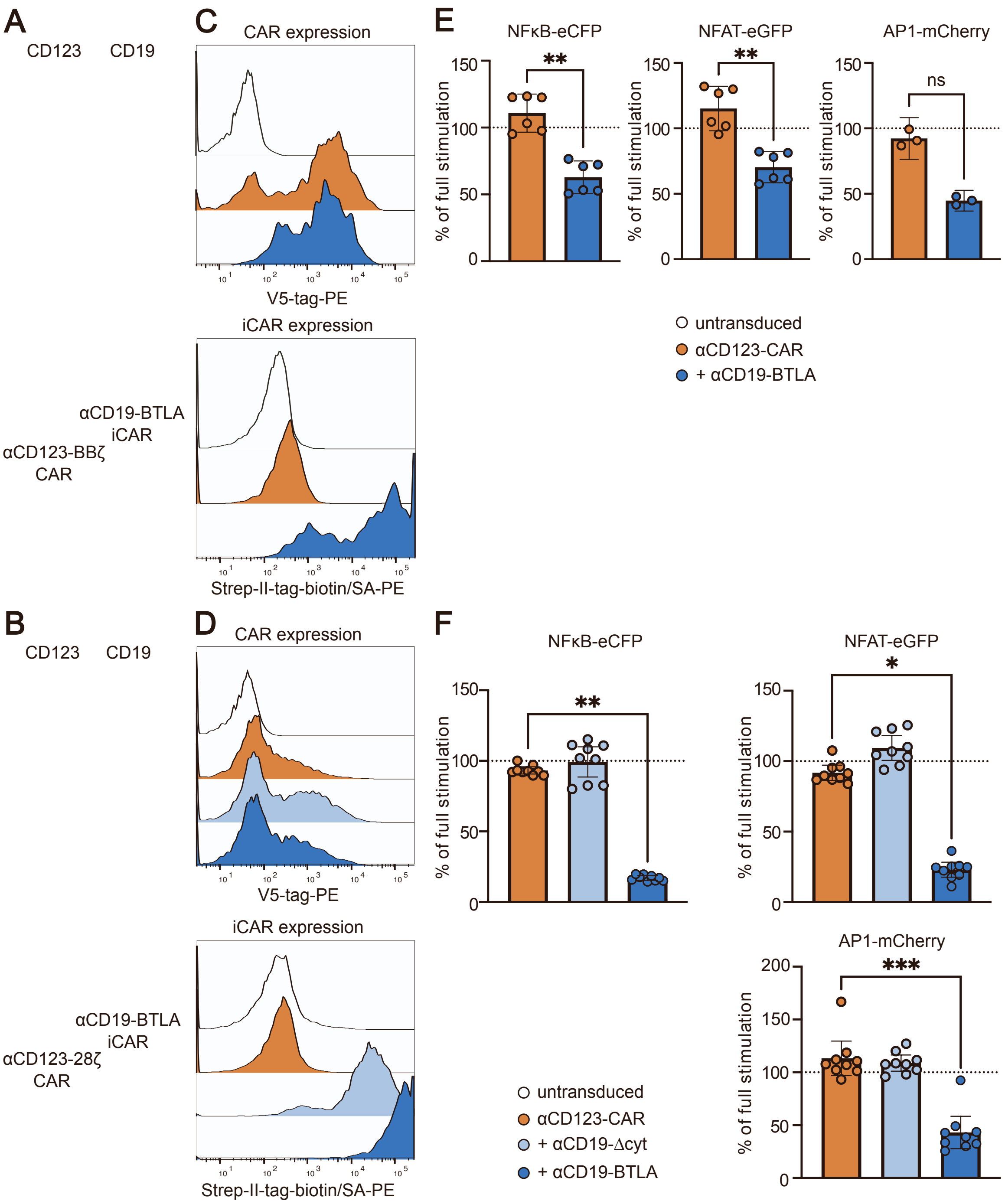
A
CD123
CD19
αCD19-BTLA
iCAR
αCD123-BBζ
CAR
B
CD123
CD19
αCD19-BTLA
iCAR
αCD123-28ζ
CAR
C
CAR expression
V5-tag-PE
iCAR expression
Strep-II-tag-biotin/SA-PE
D
CAR expression
V5-tag-PE
iCAR expression
Strep-II-tag-biotin/SA-PE
E
NFκB-eCFP
NFAT-eGFP
AP1-mCherry
% of full stimulation
**
**
ns
untransduced
αCD123-CAR
+ αCD19-BTLA
F
NFκB-eCFP
NFAT-eGFP
AP1-mCherry
% of full stimulation
**
*
***
untransduced
αCD123-CAR
+ αCD19-Δcyt
+ αCD19-BTLA

Fig. S9 **αCD19-BTLA iCAR inhibits second generation αCD123-BBζ and αCD123-28ζ CARs.**
A,B Schemes depicting assay setup to test capability of the αCD19-BTLA iCAR to suppress TPR activation by a second-generation αCD123 CARs (**A** αCD123-BBζ, **B** αCD123-28ζ). **C,D** Expression of αCD123-BBζ (**C**) and αCD123-28ζ (**D**) CAR and αCD19-BTLA iCAR (**C**) or αCD19-BTLA iCAR and αCD19-Δcyt CAR (**D**) on TPR cells. Untransduced TPR cells served as negative control. **E** TPR αCD123-BBζ and TPR αCD123-BBζ αCD19-BTLA cells were cocultured with K562 CD123 and K562 CD123 CD19. Reporter gene fluorescence intensity (NFκB-eCFP, NFAT-eGFP, AP-1-mCherry) induced by target cells coexpressing CD123 and CD19 is shown normalized to activation induced by target cells expressing CD123 only (n=2, each assay performed in triplicates). Mann-Whitney-test was performed to identify differences between groups. **F** TPR αCD123-28ζ without or with additional expression of αCD19-Δcyt or αCD19-BTLA were cocultured with K562 CD123 and K562 CD123 CD19. Reporter gene fluorescence intensity (NFκB-eCFP, NFAT-eGFP, AP-1-mCherry) induced by target cells coexpressing CD123 and CD19 is shown normalized to activation induced by target cells expressing CD123 only (n=3, each assay performed in triplicates). Kruskal-Wallis test with Dunn's multiple comparison test were performed to compare groups with αCD19-iCARs to stimulation by αCD123-ζ CAR alone. (** $p<0.01$; **** $p<0.0001$; ns, not significant not shown)

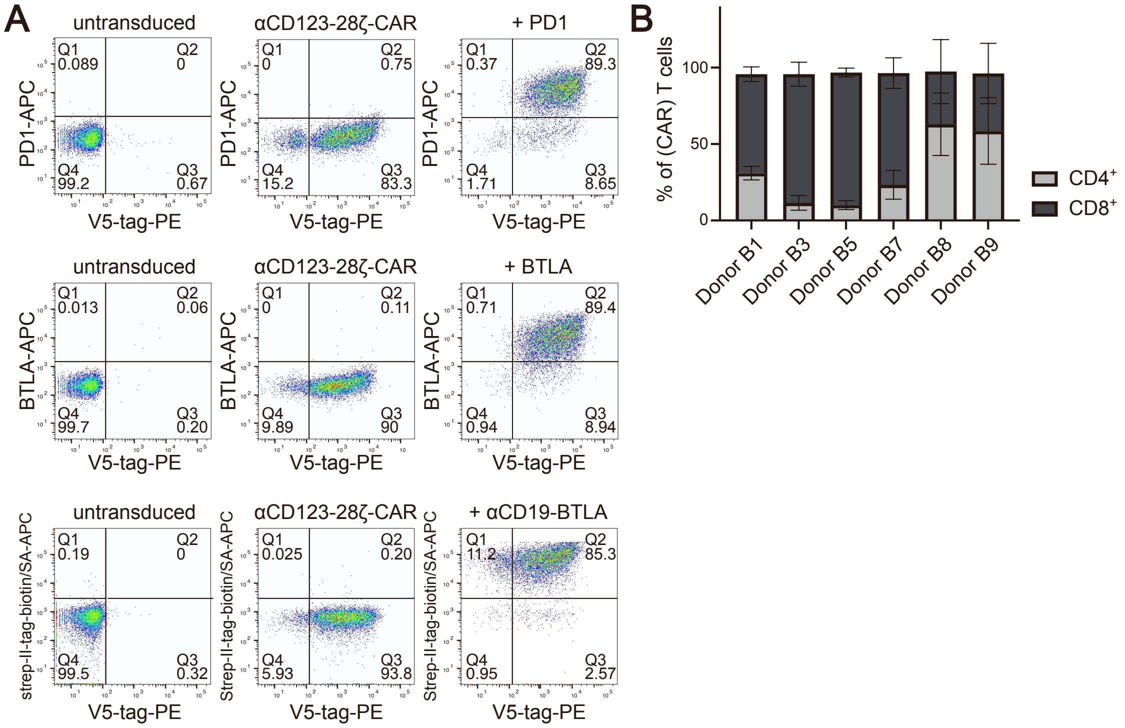

Fig. S10 **CAR T cell production.**

A Exemplary staining of untransduced T cells (left column) and CAR-T cells expressing αCD123-28ζ CAR alone (middle column) or together with inhibitory receptors (right column, PD1 (top row), BTLA (middle row) or αCD19-BTLA (bottom row)) of one donor. **B** Percentage of $CD4^+$ and $CD8^+$ T cells for each of the 6 donors. Mean percentage (95% CI) of $CD4^+$ and $CD8^+$ over all (CAR-)T cells (untransduced, αCD123-28ζ, αCD123-28ζ PD1, αCD123-28ζ BTLA and αCD123-28ζ αCD19-BTLA) derived from each donor are shown.

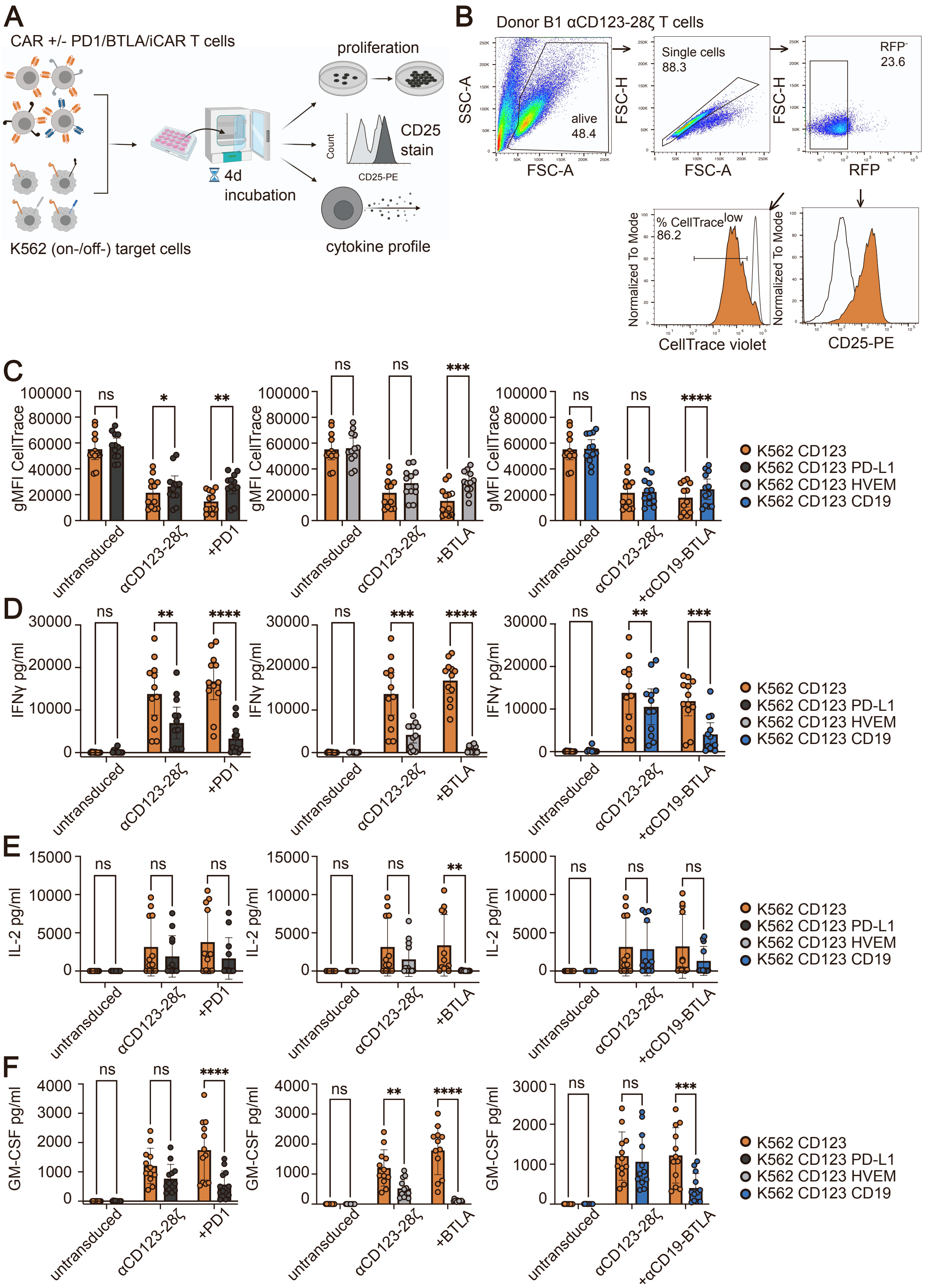
A
CAR +/- PD1/BTLA/iCAR T cells
K562 (on-/off-) target cells
4d
incubation
proliferation
CD25
stain
Count
CD25-PE
cytokine profile
B
Donor B1 αCD123-28ζ T cells
SSC-A
FSC-A
alive
48.4
FSC-H
Single cells
88.3
RFP-
23.6
RFP
Normalized To Mode
% CellTrace low
86.2
CellTrace violet
CD25-PE
C
gMFI CellTrace
untransduced
αCD123-28ζ
+PD1
+BTLA
+αCD19-BTLA
K562 CD123
K562 CD123 PD-L1
K562 CD123 HVEM
K562 CD123 CD19
D
IFNγ pg/ml
E
IL-2 pg/ml
F
GM-CSF pg/ml

Fig. S11 **Gating strategy for proliferation assay, CD25-stain and cytokine profiling of αCD123-CAR T cells. A** Scheme depicting assay set up to measure T cell effector functions (proliferation, upregulation of CD25, cytokine release). **B** Representative gating of Donor B1 αCD123-28ζ T cells to assess proliferation (CellTrace violet dilution assay) and CD25 upregulation. Live, single cells were identified by FSC-A/SSC-A, FSC-A/FSC-H gating and T cells (RFP^{-}) were analyzed as indicated. **C** Geometric mean fluorescent intensity of CellTrace violet in the proliferation assay. Data is presented analogous to Fig. 4B (percentage of $CellTrace^{low}$ T cells). Repeated measure two-way ANOVA with Šídák's multiple comparisons test was applied to compare proliferation of the indicated T cells in coculture with either K562 CD123 or with K562 CD123 expressing one of the inhibitory ligands (PD-L1, HVEM, CD19). **D/E/F** Analysis of further cytokines (**D** IFNγ, **E** IL-2, **F** GM-CSF analogous to Fig. 4C (TNFα). (*p<0.05; ** p<0.01; *** p<0.001; **** p<0.0001; ns, not significant)

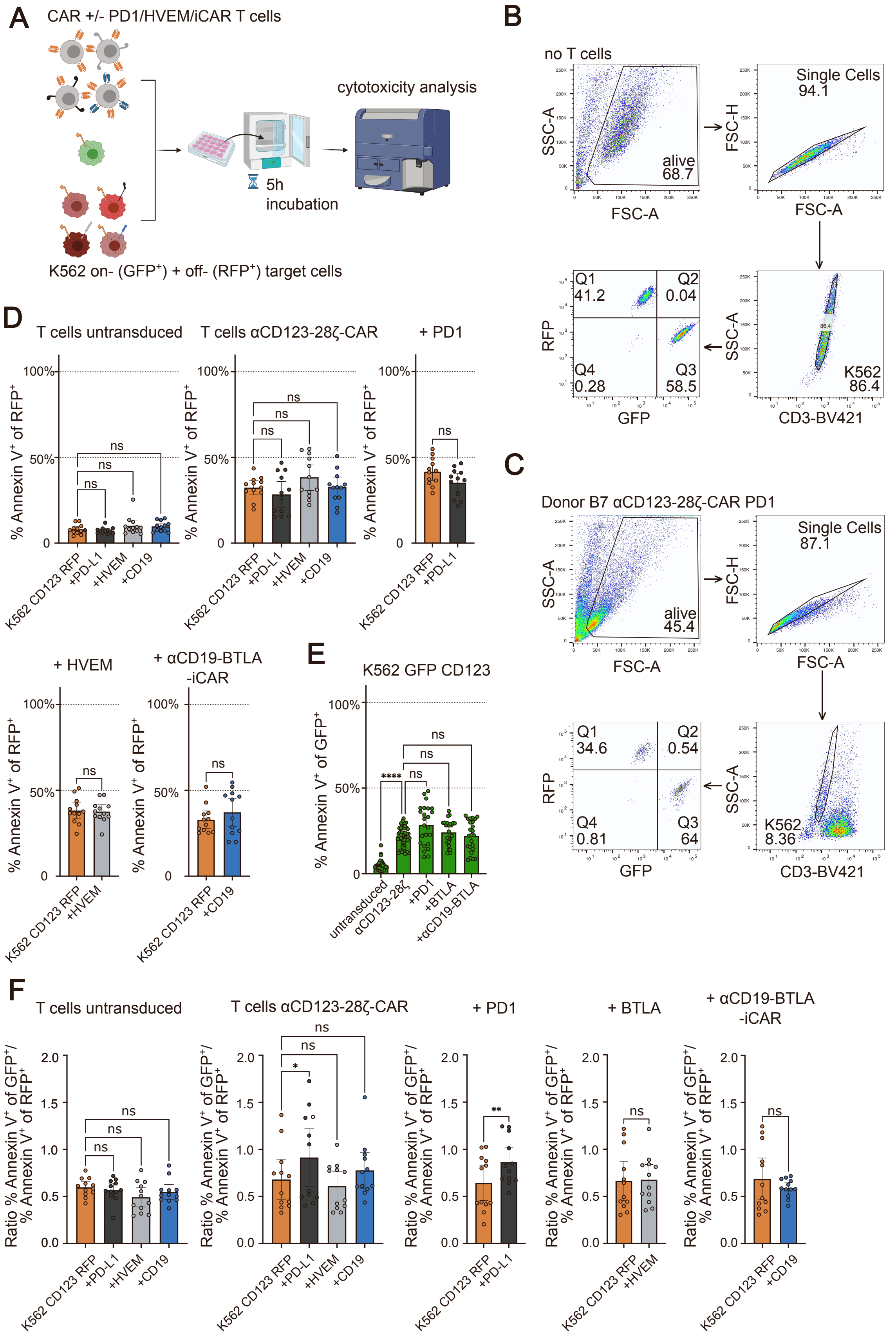
A
CAR +/- PD1/HVEM/iCAR T cells
cytotoxicity analysis
5h
incubation
K562 on- (GFP+) + off- (RFP+) target cells
B
no T cells
SSC-A
FSC-A
alive
68.7
FSC-H
Single Cells
94.1
Q1
41.2
Q2
0.04
Q4
0.28
Q3
58.5
RFP
GFP
SSC-A
K562
86.4
CD3-BV421
C
Donor B7 αCD123-28ζ-CAR PD1
alive
45.4
Single Cells
87.1
Q1
34.6
Q2
0.54
Q4
0.81
Q3
64
K562
8.36
D
T cells untransduced
T cells αCD123-28ζ-CAR
+ PD1
+ HVEM
+ αCD19-BTLA -iCAR
% Annexin V+ of RFP+
100%
50%
0
ns
K562 CD123 RFP
+PD-L1
+HVEM
+CD19
E
K562 GFP CD123
% Annexin V+ of GFP+
****
untransduced
αCD123-28ζ
+PD1
+BTLA
+αCD19-BTLA
F
T cells untransduced
T cells αCD123-28ζ-CAR
+ PD1
+ BTLA
+ αCD19-BTLA -iCAR
Ratio % Annexin V+ of GFP+/ % Annexin V+ of RFP+
2.0
1.5
1.0
0.5
0.0
*
**

Fig. S12 **Assessment of killing in a dual-target-cell killing assay. A** Scheme depicting the setup of CAR-T cell dual-target cell cytotoxicity assay. Untransduced T cells or αCD123-CAR-T cells without or with additional expression of PD1, BTLA or a αCD19-BTLA iCAR were cocultured with K562 GFP^{+} CD123 target cells and K562 RFP^{+} target cells expressing CD123 alone or together with PD-L1, HVEM or CD19 for 5h (E:T ratio of 10:1:1, n=6 donors, each assay in duplicates). Subsequently, cells were stained with Annexin-V-PE and apoptotic target cells were identified by flow cytometry. **B,C** Gating strategy for the flow cytometry based dual-target-cell cytotoxicity assay. (**B**) shows representative plots of the no T cell control, (**C**) shows representative plots of a condition with CAR-T cells. **D** Rate of Annexin-V^{+}/RFP^{+} of total RFP^{+} cells presented as mean with 95% CI. Dots represent individual repeats. Panels show data for untransduced, CAR-, CAR/PD1-, CAR/BTLA- and CAR/iCAR-transduced T cells separately. Bars indicate different RFP^{+} target cell lines (K562 CD123 (orange), K562 CD123 PD-L1 (dark grey), K562 CD123 HVEM (light grey) and K562 CD123 CD19 (blue). Comparisons between different target cell lines were analyzed by Friedman-test with Dunn's multiple comparisons test (T cell untransduced, non-normally distributed), RM one-way ANOVA with Holm-Šidák's multiple comparisons test (T cells αCD123-28ζ) or paired, two-tailed t-test (T cells αCD123-28ζ PD1, T cells αCD123-28ζ BTLA, T cells αCD123-28ζ αCD19-BTLA). **E** Rate of Annexin-V^{+} GFP^{+} of total GFP^{+} cells presented as mean with 95% CI. Dots represent individual repeats. Differences compared to killing by αCD123-28ζ T cells were analyzed by Kruskal-Wallis test with Dunn's multiple comparisons test. **F** Panels show ratio of Annexin-V^{+} of GFP^{+} cells to Annexin-V^{+} of RFP^{+} cells after coculture with the indicated T cells to detect differential killing of inhibitory ligand expressing target cells. RM one-way ANOVA test with Holm-Šidák's multiple comparisons test or paired, two-tailed t-test for normally distributed data (T cells untransduced, αCD123-ζ + PD1, αCD123-ζ + BTLA, αCD123-ζ + αCD19-BTLA) or Friedman-test with Dunn's multiple comparisons test for non-normally distributed data (T cells αCD123-ζ) was used. (* $p<0.05$; ** $p<0.01$; ns, not significant)

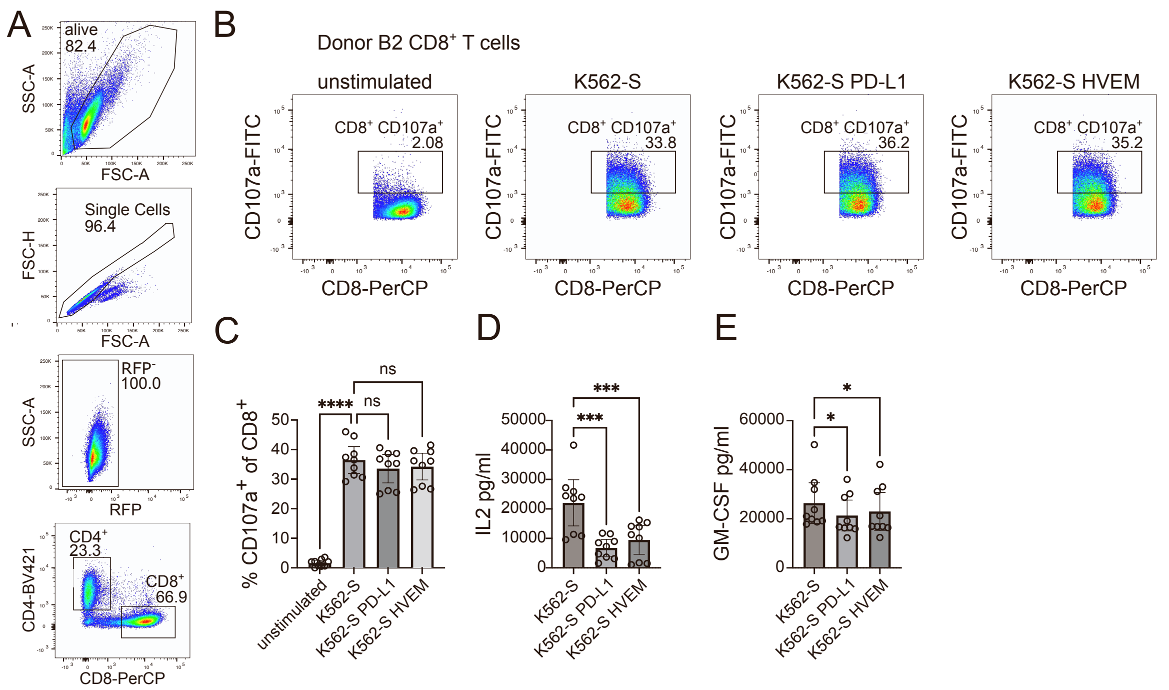

Fig. S13 **PD1 and BTLA engagement does not suppress degranulation of CD8⁺ T cells A** Gating strategy to identify CD8⁺ T cells in coculture with K562-S, K562-S PD-L1 or K562-S HVEM target cells. **B,C** T cells were cocultured with indicated target cells for 5h in presence of CD107a-FITC antibody and Golgistop/Golgiplug reagents. (**B**) Representative plots show CD107a staining of CD8⁺ T cells of one donor. (**C**) Percentage of CD107a⁺ cells of CD8⁺ T cells (n=3, performed in triplicates) **D-E** Parallel cocultures were performed for 24-48h (n=3, performed in triplicates). Then, supernatants were analyzed for cytokine concentration. Bar graphs indicate concentration of IL-2 (**D**) and GM-CSF (**E**) in culture supernatants. For statistical analysis one-way RM ANOVA with Šidák's multiple comparisons test with donor-specific matching was performed. (* p<0.05; *** p<0.001 ; **** p< 0.0001; ns, not significant)

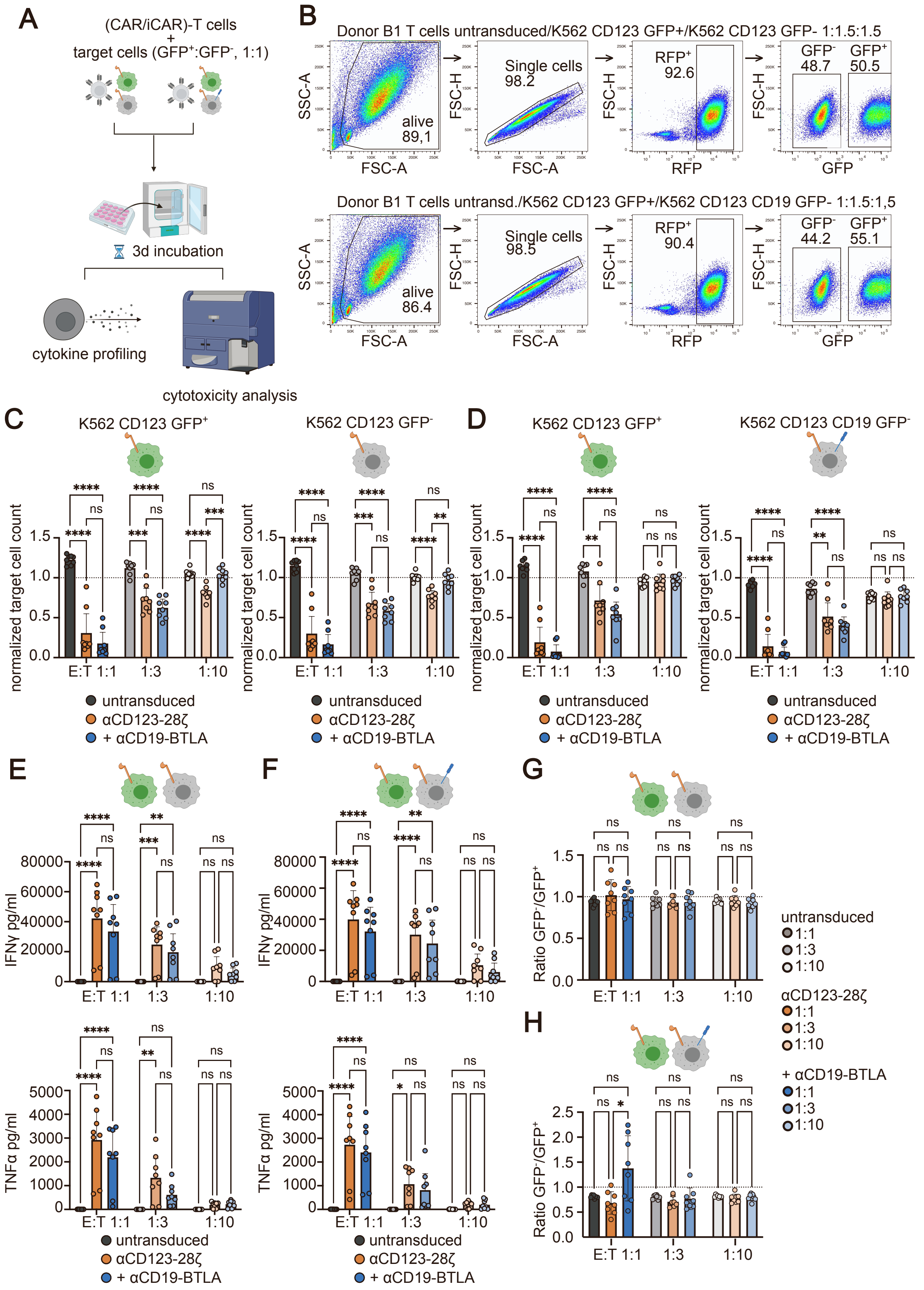
A
(CAR/iCAR)-T cells
+
target cells (GFP+:GFP-, 1:1)
3d incubation
cytokine profiling
cytotoxicity analysis
B
Donor B1 T cells untransduced/K562 CD123 GFP+/K562 CD123 GFP- 1:1.5:1.5
alive 89,1
Single cells 98.2
RFP+ 92.6
GFP- 48.7
GFP+ 50.5
Donor B1 T cells untransd./K562 CD123 GFP+/K562 CD123 CD19 GFP- 1:1.5:1,5
alive 86.4
Single cells 98.5
RFP+ 90.4
GFP- 44.2
GFP+ 55.1
SSC-A
FSC-A
FSC-H
RFP
GFP
C
K562 CD123 GFP+
K562 CD123 GFP-
D
K562 CD123 GFP+
K562 CD123 CD19 GFP-
normalized target cell count
E:T 1:1
1:3
1:10
untransduced
αCD123-28ζ
+ αCD19-BTLA
E
F
IFNγ pg/ml
TNFα pg/ml
G
Ratio GFP-/GFP+
H
untransduced
αCD123-28ζ
+ αCD19-BTLA
1:1
1:3
1:10

Fig. S14 **Cytotoxic activity and cytokine production in cocultures containing target cells expressing CD123 only as well as target cells coexpressing CD123 and CD19. A** Scheme depicting assay setup. Untransduced T cells or αCD123-28ζ CAR-T cells with or without αCD19-BTLA were cocultured for 72h with GFP^{+} K562 CD123 and either GFP^{-} K562 CD123 or GFP^{-} K562 CD123 CD19 cells. Subsequently, cytotoxicity and cytokine concentrations were assessed (n=4 donors, all conditions in duplicates). **B** Gating strategy to assess target cell counts in coculture. **C,D** Target cell counts were normalized to target cell count without T cells. Panels separately show the counts of the two indicated target cell populations in cocultures containing no target cells expressing CD19 (**C**) and of the two indicated target cell populations in cocultures containing target cells expressing CD19 (**D**). **E,F** Concentration of IFNγ (top panels) and TNFα (bottom panels) in the cocultures containing no target cells expressing CD19 (**E**) and in cocultures containing target cells expressing CD19 (**F**). **G,H** Ratio of normalized counts of GFP^{+} and GFP^{-} target cells in cocultures with GFP^{-} target cells not expressing CD19 (**G**) and GFP^{-} target cells expressing CD19 as indicated (**H**). For statistical analysis two-way RM ANOVA with Tukey's multiple comparisons test with donor-specific matching was performed. (* p<0.05; ** p<0.01; *** p<0.001 ; **** p< 0.0001; ns, not significant)
