# Supplementary material for: Inhibitory CARs fail to protect from immediate T cell cytotoxicity
Source: Mol Ther. 2024 Feb 22;32(4):982–99. doi: 10.1016/j.ymthe.2024.02.022 (PMC11163222; doi:10.1016/j.ymthe.2024.02.022)
Supplement: Document S2. Article plus supplemental information [file mmc2.pdf]

Molecular Therapy
Original Article

Inhibitory CARs fail to protect from immediate T cell cytotoxicity

Maximilian A. Funk,[1,3,4,5] Gerwin Heller,[2] Petra Waidhofer-Söllner,[1] Judith Leitner,[1] and Peter Steinberger[1]

[1]Center for Pathophysiology, Infectiology and Immunology, Institute of Immunology, Division for Immune Receptors and T Cell Activation, Medical University of Vienna, Vienna, Austria; [2]Division of Oncology, Department of Medicine I, Medical University of Vienna, Vienna, Austria; [3]University Hospital LMU Munich, Department of Medicine III, Munich, Germany; [4]Gene Center, LMU Munich, Cancer and Immunometabolism Research Group, Munich, Germany; [5]German Cancer Consortium (DKTK), Munich Site and German Cancer Research Center, Heidelberg, Germany

Chimeric antigen receptors (CARs) equipped with an inhibitory signaling domain (iCARs) have been proposed as strategy to increase on-tumor specificity of CAR-T cell therapies. iCARs inhibit T cell activation upon antigen recognition and thereby program a Boolean NOT gate within the CAR-T cell. If cancer cells do not express the iCAR target antigen while it is highly expressed on healthy tissue, CAR/iCAR coexpressing T cells are supposed to kill cancer cells but not healthy cells expressing the CAR antigen. In this study, we employed a well-established reporter cell system to demonstrate high potency of iCAR constructs harboring BTLA-derived signaling domains. We then created CAR/iCAR combinations for the clinically relevant antigen pairs B7-H3/CD45 and CD123/CD19 and show potent reporter cell suppression by iCARs targeting CD45 or CD19. In primary human T cells αCD19-iCARs were capable of suppressing T cell proliferation and cytokine production. Surprisingly, the iCAR failed to veto immediate CAR-mediated cytotoxicity. Likewise, T cells overexpressing PD-1 or BTLA did not show impaired cytotoxicity toward ligand-expressing target cells, indicating that inhibitory signaling by these receptors does not mediate protection against cytotoxicity by CAR-T cells. Future approaches employing iCAR-equipped CAR-T cells for cancer therapy should therefore monitor off-tumor reactivity and potential CAR/iCAR-T cell dysfunction.

INTRODUCTION

A general principle of oncologic therapy is to develop drugs and treatments that are more toxic to cancerous cells than healthy cells, thereby establishing a therapeutic window. This allows for effective treatment with limited adverse effects for the patient. This also applies to adoptive cell transfer therapies such as chimeric antigen receptor (CAR)-T cell therapies.[1]

CAR-T cells have very successfully been applied to treat certain cancer entities, such as B cell malignancies (B cell lymphomas and leukemias) and multiple myeloma.[2] A desired feature of CAR-T cell treatable malignancies is the presence of a highly expressed surface antigen with little to no off-tumor expression. Successful current CAR therapies target antigens such as CD19 or B cell maturation antigen (BCMA), which are coexpressed on malignant cells and healthy B cells. While αCD19-CAR-T cell therapies lead to prolonged B cell aplasia, this is a tolerable side effect that may be treated by intravenous supplementation of immunoglobulins.[3] Most cancer entities do not express unique surface antigens and target antigen coexpression on healthy tissue has led to severe on-target off-tumor (OTOT) adverse events in the case of new CAR-T cell therapies.[4–8] Of note, a recent study indicates that the immune effector cell-associated neurotoxicity syndrome (ICANS) commonly occurring with the approved αCD19-CAR-T cell therapies may be linked to CD19 expression on pericytes in the brain.[9] OTOT adverse events also occur with other adoptive T cell therapies, as illustrated by immune-mediated cardiotoxicity in the case of a T cell receptor (TCR) transgenic T cell therapy, in which a TCR specific to MAGE A3 was cross reactive to a peptide derived from the cardiac protein titin.[10]

Therefore, concerns about OTOT adverse events warrant careful evaluation of the designated target antigen expression pattern focusing on potential off-tumor ectopic expression on healthy cells.[11] This greatly limits the pool of targetable cancer antigens, hampering the expansion of the CAR-T cell therapy repertoire.[1,12–15]

A great advantage of CAR-T cell therapies is the possibility to genetically engineer the CAR-T cell product to overcome biological hurdles imposed by cancers.[16] Potential strategies to address the problem of OTOT adverse events include increasing CAR-T cell on-tumor specificity by linking multiple antigens by engineered Boolean logic-gating circuits.[1] Several approaches have shown ways to connect two antigens by an AND-gate logic, so that CAR-T cells only activate in the presence of both antigens but not either one of them alone.[17–24] These approaches might, however, increase the risk of tumor immune escape since the downregulation of either target antigen will render the tumor cells protected. While this also occurs with single-antigen targeted CAR-T cells,[2]

Received 16 October 2023; accepted 19 February 2024;
https://doi.org/10.1016/j.ymthe.2024.02.022.

Correspondence: Peter Steinberger, Center for Pathophysiology, Infectiology and Immunology, Institute of Immunology, Division for Immune Receptors and T Cell Activation, Medical University of Vienna, Vienna, Austria.
E-mail: peter.steinberger@meduniwien.ac.at

 © 2024 The Author(s).
This is an open access article under the CC BY license (http://creativecommons.org/licenses/by/4.0/).

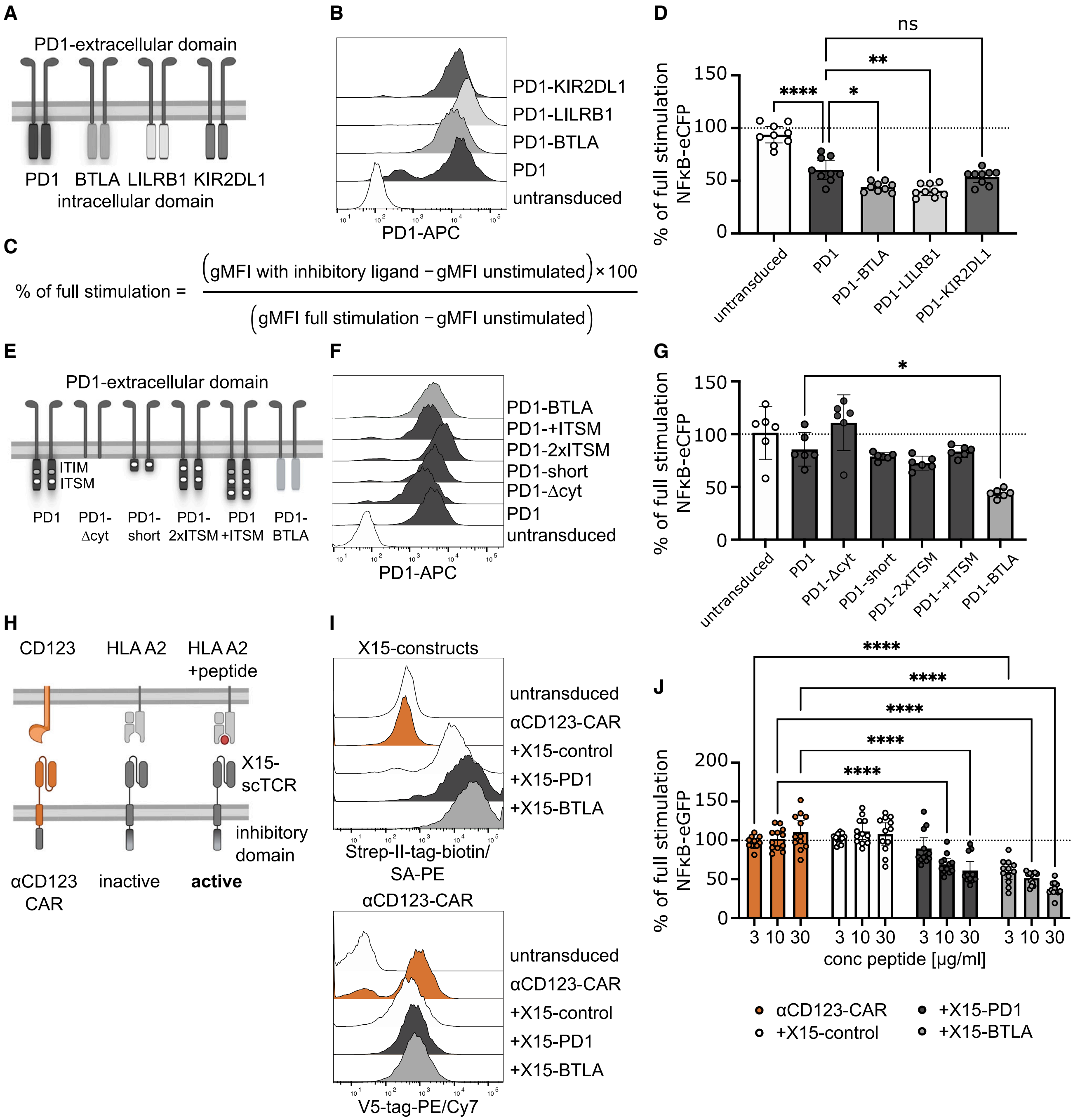

Figure 1. BTLA signaling domain potently suppresses T cell activation

(A) Scheme illustrating design of PD-1-chimera. (B) Expression of PD-1-KIRDL1, PD-1-LILRB1, PD-1-BTLA, and PD-1 constructs on TPR cells. Untransduced cells served as negative control. (C) Formula used to calculate percentage of full stimulation in reporter cells in the presence of inhibitory ligands on stimulator cells. (D) TPR expressing the indicated PD-1-chimera were cocultured with TCS or TCS PD-L1. After 24 h, NF-κB-eCFP reporter gene expression was assessed by flow cytometry. Stimulation in presence of TCS PD-L1 is expressed as percentage of stimulation by TCS (n = 3, each assay in triplicates). Data are presented as mean with 95% confidence interval (CI). Dots represent individual repeats. Brown-Forsythe and Welch ANOVA tests with Dunnett's T3 multiple comparisons test were used to compare all groups to inhibition by PD-1. (E) Scheme illustrating PD-1 constructs with engineered signaling domains (PD-1-Δcyt, -short, -2xITSM, -+ITSM, -BTLA). (F) Expression of PD-1-constructs with engineered signaling domains on TPR cells. Untransduced cells served as negative control. (G) Assay setup was analogous to (C), except TPR expressing the PD-1-constructs with engineered signaling domains were used (n = 2, each assay in triplicates). Data are presented as mean with 95% CI. Dots represent individual repeats.

(legend continued on next page)

this escape mechanism is likely to increase if two antigens are required for proper CAR-T cell functionality.

Mechanistically closely linked to the AND-logic approaches are those that include signaling circuits that "turn on" CAR-T cells if certain exogenous stimuli, such as the right tumor environment, is present (IF/THEN logic).[25–29] However, presence of specific tumor microenvironmental features may not be perfectly stable and tumor specific, especially in multilocular disease and over a prolonged disease duration. This can provide potential pathways for tumor cell persistence and OTOT adverse events.[1] Another potential logic-gating strategy is combining a cancer antigen with a non-cancer antigen in a NOT-gate fashion. While the cancer antigen is present on both cancer and healthy cells, the non-cancer antigen is exclusive to healthy cells and, in its presence, CAR-T cell activation against the expressing cell is blocked.[30,31] The concept was first presented using inhibitory chimeric antigen receptors that consist of an antigen recognition domain fused to a cytotoxic T lymphocyte (CTL)-associated protein 4 (CTLA4) or a programmed cell death protein 1 (PD-1) signaling domain that triggers an antigen-specific immune checkpoint in the presence of a non-cancer antigen.[30] Others have adapted this strategy, identifying alternative inhibitory signaling domains from receptors such as T-cell immunoreceptor with immunoglobulin and ITIM domains (TIGIT), Leukocyte immunoglobulin-like receptor subfamily B member 1 (LILRB1), and B- and T -lymphocyte attenuator (BTLA) as suitable for iCAR design.[32–35] However, to our knowledge, so far no CAR/iCAR combination suitable to address a clinically relevant OTOT problem has been investigated. We have previously established a Jurkat-based T cell reporter system that is suitable to analyze costimulatory and inhibitory receptors on T cells.[36] Here, we applied this reporter cell system to study CAR/iCAR-T cells. For this, we first identified potent inhibitory signaling domains. Next, we analyzed publicly available single-cell RNA sequencing (scRNA-seq) datasets to identify clinically relevant CAR/iCAR-antigen combinations. We then went on to create the respective CAR and iCAR molecules and tested their functionality in the reporter cell system. Finally, we expressed a selected CAR/iCAR combination in primary T cells to evaluate iCAR-mediated suppression of T cell proliferation, upregulation of CD25, and cytokine release as well as the capability of iCARs to prevent cytotoxicity toward target cells coexpressing the iCAR antigen.

RESULTS

BTLA signaling domain potently suppresses T cell activation

Previous iCAR molecules have been designed using signaling domains derived from various immune checkpoint receptors, including CTLA4, PD-1, BTLA, LILRB1, and TIGIT.[30,32,33,35] To test which of the proposed signaling domains would be most suitable for an iCAR design, a Jurkat-based reporter cell system was applied for head-to-head comparison of isolated inhibitory signaling domains. The well-established T cell inhibitory receptor PD-1 was used as a reference molecule. Through molecular cloning, we generated PD-1 chimera where the intracellular domain of PD-1 was replaced with the signaling domains of BTLA, LILRB1, an inhibitory receptor for major histocompatibility complex (MHC) class I molecules, and KIR2DL1, which is an inhibitory receptor on natural killer (NK) cells (Figure 1A).[37] BTLA and LILRB1 were chosen because previous studies have shown inhibition of CAR-T cell activation by these molecules.[33,35] According to the "missing self" hypothesis, inhibitory NK cell receptors protect cells expressing MHC class I, while downregulation of MHC class I results in NK cell-mediated killing.[38] This mode of activation resembles the NOT gate we aimed to establish in CAR-T cells and therefore KIR2DL1 was chosen to be evaluated as a signaling module for iCARs. Since the inhibitory mechanism of CTLA4 at least in part depends on its extracellular domain, and it was previously shown to be inferior to PD-1 in other studies, it was not included in this evaluation.[30,39–43] Similarly, since an αCD19-TIGIT iCAR was reported to induce tonic, antigen-independent inhibitory signaling, we did not use it in our study.[32] As CAR and therefore also iCAR signaling is highly dependent on target antigen density, CAR/iCAR affinity and expression levels,[30,44] careful monitoring of chimeric PD-1-construct expression levels was warranted and close matching of the different constructs was ensured (Figure 1B). To compare the inhibitory capability of the inhibitory domains, Jurkat triple parameter reporter (TPR) cells harboring the chimeric PD-1 constructs were cocultured with T cell stimulator (TCS) cells[45] expressing a membrane-bound anti-CD3 scF_v (mb-α-CD3) alone or TCS coexpressing mb-α-CD3 and programmed death-ligand 1 (PD-L1). Following 24 h of stimulation, the activation of the nuclear factor κB-enhanced cyan fluorescent protein (NF-κB-eCFP) and nuclear factor of activated T cells-enhanced green fluorsecent protein (NFAT-eGFP) reporters was analyzed by flow cytometry (Figures S1A–S1C). Inhibition was then calculated as ratio of geometric mean fluorescence intensity in the presence of PD-L1 to stimulation without PD-L1 (Figure 1C). With this well-controlled, head-to-head assay it could be shown that BTLA and LILRB1 exhibited significantly superior suppression of NF-κB-eCFP and NFAT-eGFP reporter gene induction in comparison to PD-1 (Figures 1D and S1D). The activator protein 1 (AP1)-mCherry signal was hardly detectable in this Jurkat TPR sub-cell line and was therefore not evaluated. Importantly, the inhibitory effect of all chimeric PD-1-constructs was reverted by addition of nivolumab, a PD-1-blocking antibody, confirming the specificity of the inhibitory effect (Figures S1B and S1C). In the next set of experiments, the possibility of enhancing the inhibitory capability of PD-1 by engineering additional

Kruskal-Wallis test with Dunn's multiple comparisons test was used to compare all groups to inhibition by PD-1. (H) Scheme depicting assay setup to study inhibition of an αCD123-CAR by ×15 constructs. (I) Expression of X15-Δcyt, X15-PD-1, and X15-BTLA constructs and αCD123-CAR on TPR cells. Untransduced cells served as negative control. (J) TPRs expressing αCD123-CAR alone or together with X15-iCARs were cocultured with K562 CD123 HLA-A2 without or with addition of Tax peptide. X15-iCAR-mediated inhibition was expressed as percentage of full stimulation (without peptide) reached in the presence of the indicated amount of peptide (n = 4, each assay in triplicate). Data are presented as mean with 95% CI. Dots represent individual repeats. Brown-Forsythe and Welch ANOVA tests with Dunnett's T3 multiple comparisons test was used to compare all groups to stimulation with αCD123-CAR alone (*$p < 0.05$; **$p < 0.01$; ****$p < 0.0001$; ns, not significant).

immunoreceptor tyrosine-based switch motif (ITSM) phosphatase-binding motifs was explored. In addition to wild-type PD-1 and the previously tested PD-1-BTLA chimeric molecule, four PD-1 molecules with engineered signaling domains were tested: PD-1-Δcyt, lacking any signaling domain; PD-1-short, featuring only the ITSM motif that is indispensable for PD-1 signaling[46]; PD-1-2xITSM, in which the non-functional immunoreceptor tyrosine-based inhibitory motif (ITIM) is replaced by an ITSM motif; and PD-1-+ITSM, in which the PD-1-short signaling domain was fused to wild-type PD-1 (Figure 1E). High expression of all constructs was confirmed by flow cytometry (Figure 1F). As expected, the PD-1-Δcyt molecule did not induce any inhibitory effect and the PD-1-short molecule performed comparably to wild-type PD-1 in accordance to reports that identify the ITSM motif as being indispensable for PD-1 signaling.[46–48] Additional ITSM sites did not confer enhanced inhibition. Also in these experiments, the PD-1-BTLA chimeric molecule outperformed wild-type PD-1, underscoring the superior inhibitory potency of BTLA over PD-1 on NF-κB and NFAT transcription (Figures 1G and S1E). While CD28-mediated costimulation is dispensable for T cell cytotoxicity,[49,50] it is crucial for initial T cell activation, and costimulatory domains are an essential part of currently used CAR-T cell constructs.[51,52] We therefore explored how inhibitory signaling mediated by PD-1 and the PD-1-BTLA, PD-1-LILRB1, and PD-1-KIRDL1 chimeric constructs would affect costimulation by CD28. Jurkat TPR cells expressing the respective molecules were cocultured with TCS CD86 and TCS CD86 PD-L1. Similar to the coculture experiment without CD86, there was a marked decrease in NF-κB-eCFP and NFAT-eGFP reporter gene transcription in cocultures of TCS CD86 PD-L1 with Jurkat TPR expressing one of the inhibitory receptors (Figure S2). Interestingly, in this experiment, BTLA outperformed the LILRB1 construct. Taken together with the previous results, this led us to pursue further experiments using BTLA as inhibitory signaling domain.

Finally, to further characterize how antigen density would influence iCAR signaling, we constructed iCAR molecules with an antigen recognition domain derived from the X15 single-chain TCR, a CAR-like molecule that recognized the HTLV-1 Tax peptide presented on human leukocyte antigen (HLA) A2.[53] Using this model, we could explore antigen-“dose” dependency of the inhibitory molecules by pulsing HLA-A2-expressing stimulator cells with various concentrations of Tax peptide (Figure 1H). High expression of all construct on the reporter cells was confirmed by flow cytometry (Figure 1I). In this experiments we used an activating CAR to deliver "signal 1" to better model the intended CAR/iCAR interaction. As expected, X15-Δcyt-expressing reporter cells did not show any inhibition in the presence of Tax peptide, while both X15-PD-1 and X15-BTLA-expressing reporter cells showed reduced activation in a Tax-peptide dose-dependent manner (Figure 1J). Importantly, the BTLA signaling domain was superior to the PD-1 signaling domain and mediated significant inhibition also at low doses of Tax peptide, emphasizing its usefulness as an iCAR-signaling domain that may function even with antigens expressed at low density. All in all, our results indicate that, because of its strong inhibitory potency and high sensitivity, the BTLA signaling domain might be highly suitable for the design of iCAR molecules.

Identifying disease relevant CAR/iCAR combinations

We aimed to develop iCARs that could restrain activating CARs targeting antigens with high risk for OTOT adverse reactions. One model comprised the antigen CD123 in acute myeloid leukemia (AML) and blastic plasmacytoid dendritic cell neoplasia (BPDCN). While CD123 is an antigen expressed on BPDCN cells and both AML blasts and importantly also on leukemic stem cells (LSCs), it is also found on certain hematopoietic progenitor cells.[54–56] Targeting CD123 may lead to severe hematotoxicity and potentially aplastic anemia.[15] Current approaches of targeting CD123 therefore pursue a bridge-to-transplant strategy, in which αCD123-CAR-T cells are used to induce remission but have to be depleted before rescue by hematopoietic stem cell transplantation (HSCT).[57] CAR-T cell depletion, however, may put the patient at higher risk for relapse, because sustained remission is associated with CAR-T cell persistence.[58] If hematopoietic stem cells (HSCs) could be engineered to express a “safety” iCAR ligand (e.g., CD19), CAR-T cells would be able to discriminate healthy $CD123^+$ $CD19^+$ hematopoietic cells from malignant $CD123^+$ $CD19^-$ cells. As the second model, we chose B7-H3, which has been implicated as a suitable target for CAR-T cell therapy because it is expressed on many cancer entities and has limited expression in healthy tissue.[59,60] However, while tissue expression of B7-H3 is generally low, it is highly expressed on cells that have central roles in immunity, including myeloid cells such as macrophages and dendritic cells and also on activated T cells.[60,61] We therefore reasoned that a CD45-directed iCAR protecting cells of hematopoietic origin would likely increase safety of B7-H3-directed CAR-T cell therapies. To verify that these target antigen combinations play a clinically meaningful role, we reanalyzed a publicly available single-cell transcriptomics dataset[62] of primary AML and healthy bone marrow samples for the expression of *IL3RA* (CD123) and *CD19* (CD19). For this, we first annotated cell types to clusters of healthy donor bone marrow cells by identification of marker genes (Figure S3). Within these clusters, expression of *CD19* was restricted to B cells while *IL3RA* was mainly expressed in the HSC/progenitor cell cluster and to a lesser extent the myeloid cell cluster (potentially plasmacytoid dendritic cells) and plasma cells (Figures 2A and 2B). In AML samples, *IL3RA* was broadly expressed in the malignant cells, while *CD19* was mostly restricted to the microenvironmental cell compartment, most likely consisting of B cells. Some expression of *CD19* was noted in malignant cells, all stemming from a single patient with $CD19^+$ AML (Figures 2C and 2D). To analyze the expression pattern of *PTPRC* (CD45) and *CD276* (B7-H3), we accessed scRNA-seq datasets of cells from patients with glioblastoma[63] (Figures 2E and 2F), glioma[64] (Figures S4A–S4C), and a patient with melanoma[65] (Figures S4D–S4F) for the expression of the respective molecules. Cell type annotations were already available at the single-cell portal (Figures 2E, S4A, and S4D). As expected, hematopoietic cells

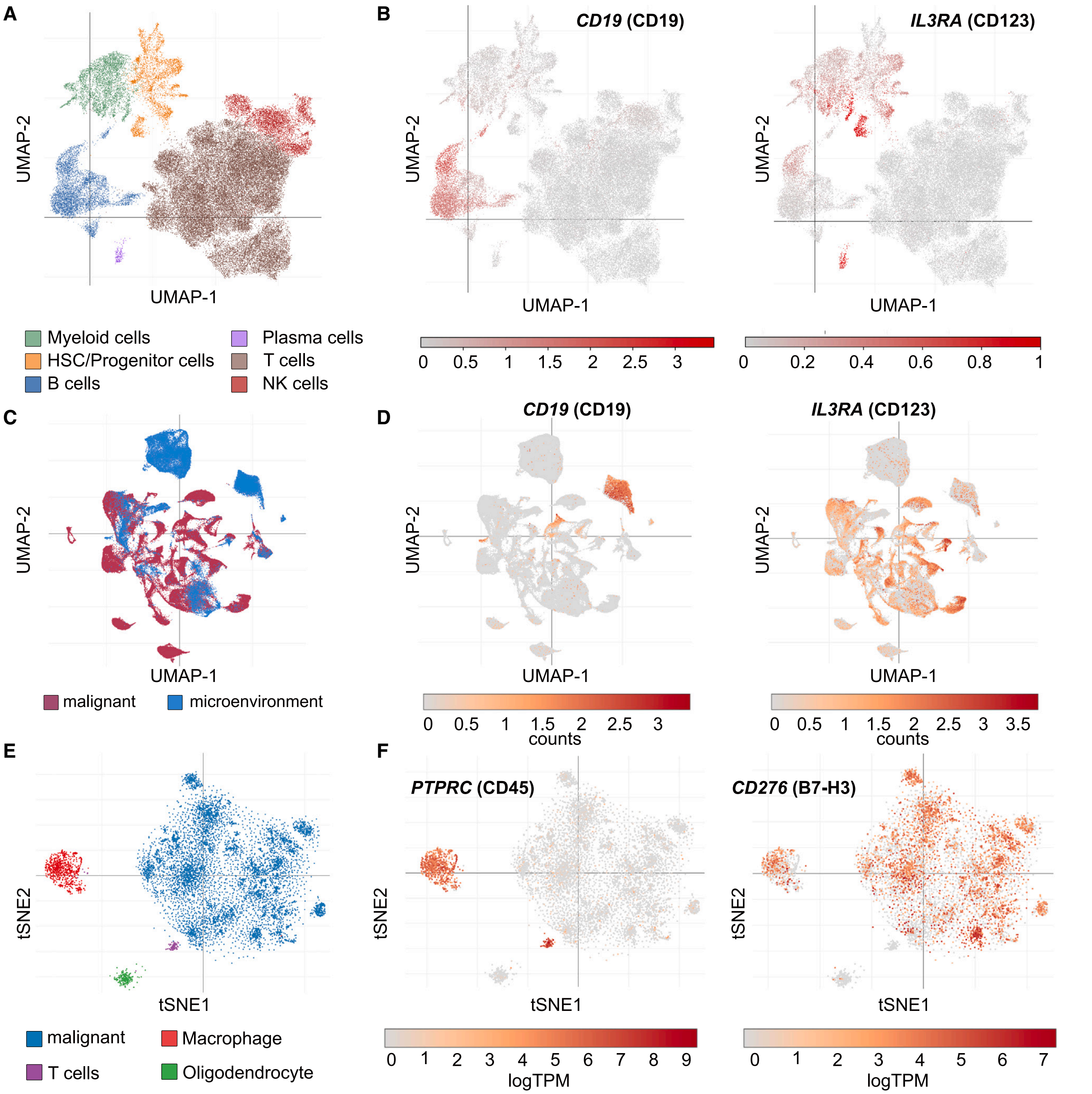

Figure 2. Identifying disease-relevant CAR/iCAR combinations in publicly available single-cell transcriptomic datasets

(A) UMAP projection of cells from healthy donor bone marrow (n = 10) showing cell type annotation as defined by marker genes (Figure S2). (B) UMAP projections indicating expression of *CD19* (CD19) (left) and *IL3RA* (CD123) (right) in healthy control bone marrow cells. (C) UMAP projection of bone marrow cells from adult (n = 20) and pediatric (n = 22) AML patients (subsampling of 100,000 cells). Annotation indicates malignant and microenvironmental cells in patient bone marrow as defined in the metadata to this dataset.[62] (D) UMAP projections indicating expression of *CD19* (CD19) (left) and *IL3RA* (CD123) (right) in bone marrow cells from AML patients. (E) t-distributed stochastic neighborhood embedding (tSNE) projection showing the cell type annotation in samples from glioblastoma patients (n = 28, 24,131 cells total) as defined in the metadata to this dataset.[63] (F) tSNE projections indicating expression pattern of *PTPRC* (CD45) (left) and *CD276* (B7-H3) (right).

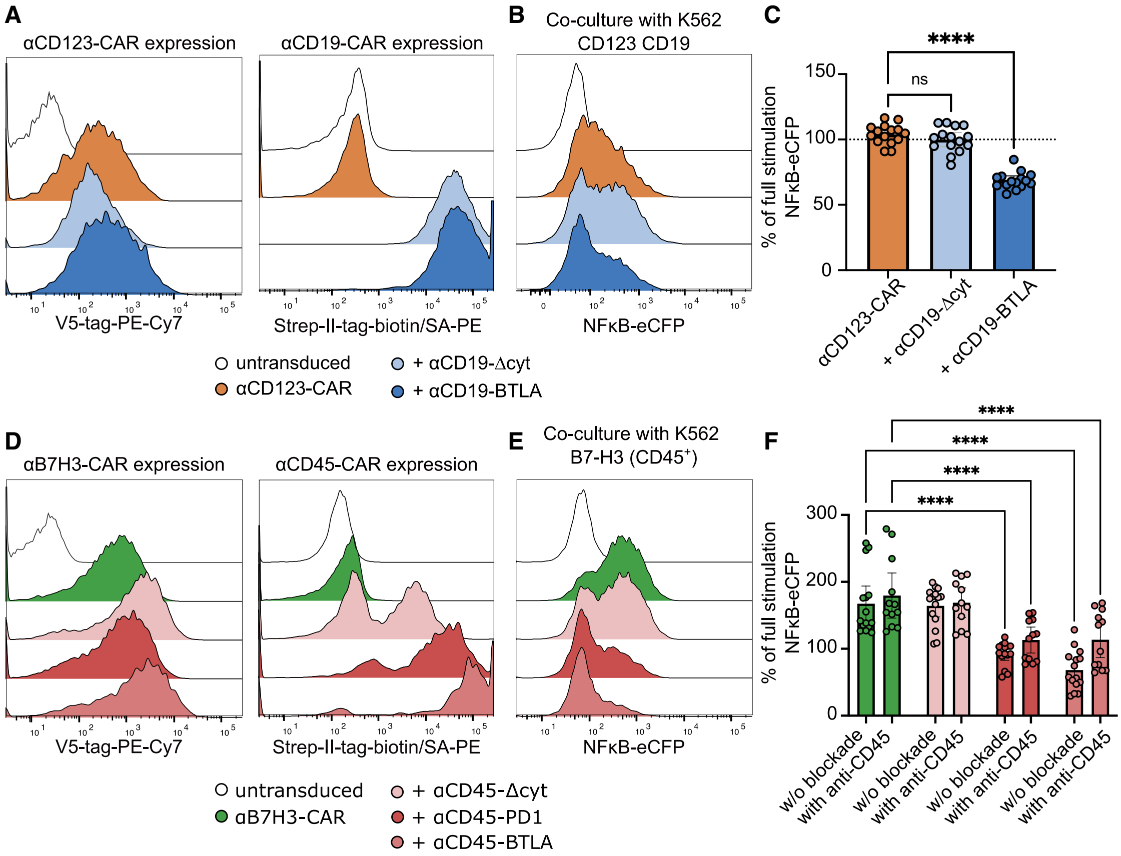

Figure 3. αCD19- and αCD45-BTLA iCARs block T cell activation by αCD123- and αB7H3-ζ CARs

(A) Expression of αCD123-CAR and αCD19-iCAR constructs on TPR cells. Untransduced cells served as negative control. (B and C) TPR expressing an αCD123-CAR alone or together with the indicated αCD19-iCARs were cocultured with K562 CD123 or K562 CD123 CD19 (n = 5, each assay in triplicate). Inhibition was expressed as percentage of full stimulation by K562 CD123. (B) Representative histograms showing NF-κB-eCFP fluorescence intensity of the indicated TPR cell lines in coculture with K562 CD123 CD19. (C) Pooled data presented as mean with 95% CI. Dots represent individual repeats. Brown-Forsythe and Welch ANOVA tests with Dunnett's T3 multiple comparisons test was performed to compare groups with αCD19-iCARs to stimulation by αCD123-ζ CAR alone. (D) Expression of αB7H3-CAR and αCD45-iCAR constructs on TPR cells. Untransduced cells served as negative control. (E and F) TPR expressing an αB7H3-ζ CAR alone or together with the indicated αCD45-iCARs or αCD45-Δcyt were co-cultured with K562 B7-H3 $CD45^{KO}$ or K562 B7-H3. Some assays were also performed in presence of an anti-CD45 antibody (n = 5/n = 4 with anti-CD45 antibody, each assay in triplicate). Inhibition was expressed as percentage of full stimulation by K562 B7-H3 $CD45^{KO}$. (E) Representative histograms showing NF-κB-eCFP fluorescence intensity of the indicated TPR cell lines in coculture with K562 B7-H3 ($CD45^{+}$). (F) Pooled data presented as mean with 95% CI. Dots represent individual repeats. Two-way ANOVA with Dunnett's multiple comparisons test was performed to compare all groups to stimulation by αB7H3-ζ CAR alone (****$p < 0.0001$; ns, not significant).

(T cells, B cells, and myeloid cells) expressed high levels of *PTPRC* (Figure 2F left panel, S4B, and S4E). Expression of *CD276* occurred in malignant cells and in myeloid cells (macrophages/monocytes) (Figure 2F right panel, S4C, and S4F). In this dataset, T cells expressing *CD276* (B7-H3) were not identified, potentially because of a lack of strongly activated T cells in the analyzed samples. In conclusion, single-cell-level transcriptomic data of multiple primary human cancers support our choice of CAR/iCAR target antigen combinations.

αCD19- and αCD45-BTLA iCARs block T cell activation by αCD123- and αB7H3-ζ CARs

To create CAR molecules with the desired specificities, V_H and V_L chain sequences of a CAR or therapeutic antibodies targeting B7-H3, CD123, and CD45, respectively, were derived from patent literature or the ImMunoGeneTics (IMGT) database.[66–69] These sequences were incorporated in a first-generation CAR backbone (Figure S5A). The CARs were then expressed in reporter cells (Figure S5B) and their functionality was validated in coculture assays using engineered K562 cell lines expressing the respective surface antigens as target cells (Figures S5C, S5D, and S6). For all assays involving CD45-CARs or -iCARs, Jurkat TPR $CD45^{KO}$ were used to avoid stimulator cell independent antigen ligation (Figure S5C). All CAR domains induced NF-κB-eCFP reporter cell transcription in the presence of the respective antigen on stimulator cells (Figures S5E–S5G). Having validated the functionality of the CAR molecules, we next wanted to test CAR/iCAR combinations in the reporter cell system. As outlined earlier, an αCD19-iCAR

could be useful to suppress αCD123-CAR-T cells targeting AML. In combination with engineered HSPCs expressing the respective safety ligand (CD19), this could prevent hematotoxicity in αCD123-CAR-T cell therapy (Figure S7A). Therefore, an αCD19-BTLA iCAR as well as a signaling-incompetent αCD19-Δcyt CAR were created from a well-established αCD19-activating CAR.[70] Comparable expression of CAR and iCAR was confirmed for all cell lines by flow cytometry (Figure 3A). In a coculture assay, the αCD19-BTLA iCAR but not the αCD19-Δcyt CAR significantly inhibited transcription of the NF-κB-eCFP reporter gene induced by the αCD123-ζ CAR in the presence of CD19 on stimulator cells (Figures 3B and 3C). Importantly, the same could be observed with the NFAT-eGFP and even the AP1-mCherry reporter genes, although, as stated previously, the AP1-mCherry signal was very weak in this sub-cell line (Figures S8A–S8D). The second CAR/iCAR combination is schematically illustrated in Figure S7B. Here, αCD45-iCARs are used to protect healthy hematopoietic cells in the context of αB7H3-CAR-T cell therapy. Similar to the previous experiment, αCD45-PD-1 and αCD45-BTLA iCARs, as well as an αCD45-Δcyt CAR were generated and expression of all constructs was confirmed by flow cytometry (Figure 3D). For validation of the functionality of the αB7H3-CAR/αCD45-iCAR combination, we used K562 cells expressing B7-H3 (Figure S5D). K562 cells are $CD45^{+}$ and, to generate target cells lacking the iCAR target, CD45 was knocked out on B7-H3-expressing K562 cells (Figure S5C). Again, coculture assays with stimulator cells were performed. These showed that, in reporter cells coexpressing an αB7H3-CAR with αCD45-PD-1 or αCD45-BTLA iCARs, reporter genes for all transcription factors were significantly inhibited by the presence of CD45 on target cells. As expected, there was no inhibition mediated by the αCD45-Δcyt CAR. The iCAR-mediated inhibition was partly reversible by addition of an antibody to CD45, underscoring the antigen dependency of the inhibitory effect (Figures 3E, 3F, and S8E–S8H). Thus, we present two possible CAR/iCAR combinations that could address clinically relevant OTOT adverse reactions.

αCD19-BTLA iCAR inhibits second-generation αCD123-28ζ and αCD123-BBζ CARs

Since current CAR molecules harbor a costimulatory signaling domain, we also wanted to confirm that our αCD19-BTLA iCAR also efficiently inhibits T cell reporter activation mediated by second-generation CARs, which are the current standard in CAR design.[71] Therefore, we generated αCD123-BBζ and αCD123-28ζ second-generation CARs and coexpressed them with an αCD19-BTLA iCAR in our reporter cells (Figures S9A and S9B). Surface expression of αCD123-CARs and αCD19 iCAR was confirmed by staining for V5- and strep-II tags, respectively (Figures S9C and S9D). In coculture assays with CD123 and CD123/CD19-expressing stimulator cells, the αCD19-BTLA iCAR significantly inhibited activation by both second-generation αCD123-CARs (Figures S9E and S9F). The particularly strong inhibition of reporter gene transcription in cells expressing the αCD123-28ζ CAR is most likely attributable to the overall low expression of this CAR (Figure S9D). Importantly, the expression of both activating CARs was similar or higher in reporter cell lines coexpressing additional αCD19-BTLA or αCD19-Δcyt when compared to the cell line expressing the respective activating CAR alone. Therefore, it can be ruled out that reduced activation in the presence of the iCAR is an artifact caused by lower expression of the activating CAR. These results show that the αCD19-BTLA iCAR can suppress activation by second-generation CARs, while highlighting the importance of fine-tuning CAR and iCAR signal strength to achieve the optimum between on-tumor activation and off-tumor inhibition.

αCD19-BTLA iCAR dampens proliferation, cytokine secretion, and upregulation of CD25

Based on these encouraging results, we decided to translate the αCD123-28ζ/αCD19-BTLA combination to primary human T cells and evaluate the impact of iCAR engagement on proliferation, cytokine release, and upregulation of the T cell activation marker CD25. As positive inhibitory control, we overexpressed PD-1 and BTLA to benchmark the αCD19-BTLA iCAR potency to established immune checkpoints. Similar expression of CD123 and high expression of the inhibitory ligands (PD-L1, herpesvirus entry mediator [HVEM]) and the iCAR antigen (CD19) on the K562 cells that were used as target cells was confirmed (Figure S6). CAR-T cells were manufactured from donor peripheral blood mononuclear cells (PBMCs) and to achieve a pure and high-expressing CAR-T cell product, desired CAR-T cells were enriched by flow sorting (Figure S10). Cells were cocultured for 4 days and analyzed by flow cytometry and multiplex cytokine measurement (Figures S11A and S11B). Indeed, we found significantly reduced proliferation of PD-1, BTLA, and αCD19-BTLA-expressing CAR-T cells under conditions where the checkpoint receptor was engaged by its ligand (Figures 4A, 4B, and S11C). We also analyzed cytokine secretion by CAR-T cells (Figures 4C and S11D–S11F). CAR-T cells expressing PD-1, BTLA, or the αCD19-BTLA iCAR did secrete significantly less tumor necrosis factor alpha (TNFα), interferon gamma (IFNγ), and granulocyte-macrophage colony-stimulating factor (GM-CSF) and showed a clear tendency to lower secretion of interleukin (IL)-2. Furthermore, the upregulation of the activation marker CD25 was significantly reduced in the presence of inhibitory ligands (Figure 4D). In line with the stronger effect of the BTLA signaling domain observed in our reporter experiments, BTLA mediated stronger suppression than PD-1. Notably, proliferation and cytokine secretion tended to be reduced in cocultures of αCD123-28ζ CAR-T cells with stimulator cells expressing HVEM or PD-L1 even if there was no overexpression of BTLA or PD-1, respectively. This was most likely due to endogenous expression of these checkpoint receptors in T cells. These results indicate that iCARs similar to checkpoint receptors are able to block T cell effector functions such as proliferation, cytokine release, and upregulation of activation markers.

αCD19-BTLA iCAR does not prevent cytotoxicity by primary αCD123-CAR-T cells *in vitro*

Next, we also assessed the killing capability of these CAR-T cells. Therefore, we set up a flow cytometry-based killing assay. CAR-T

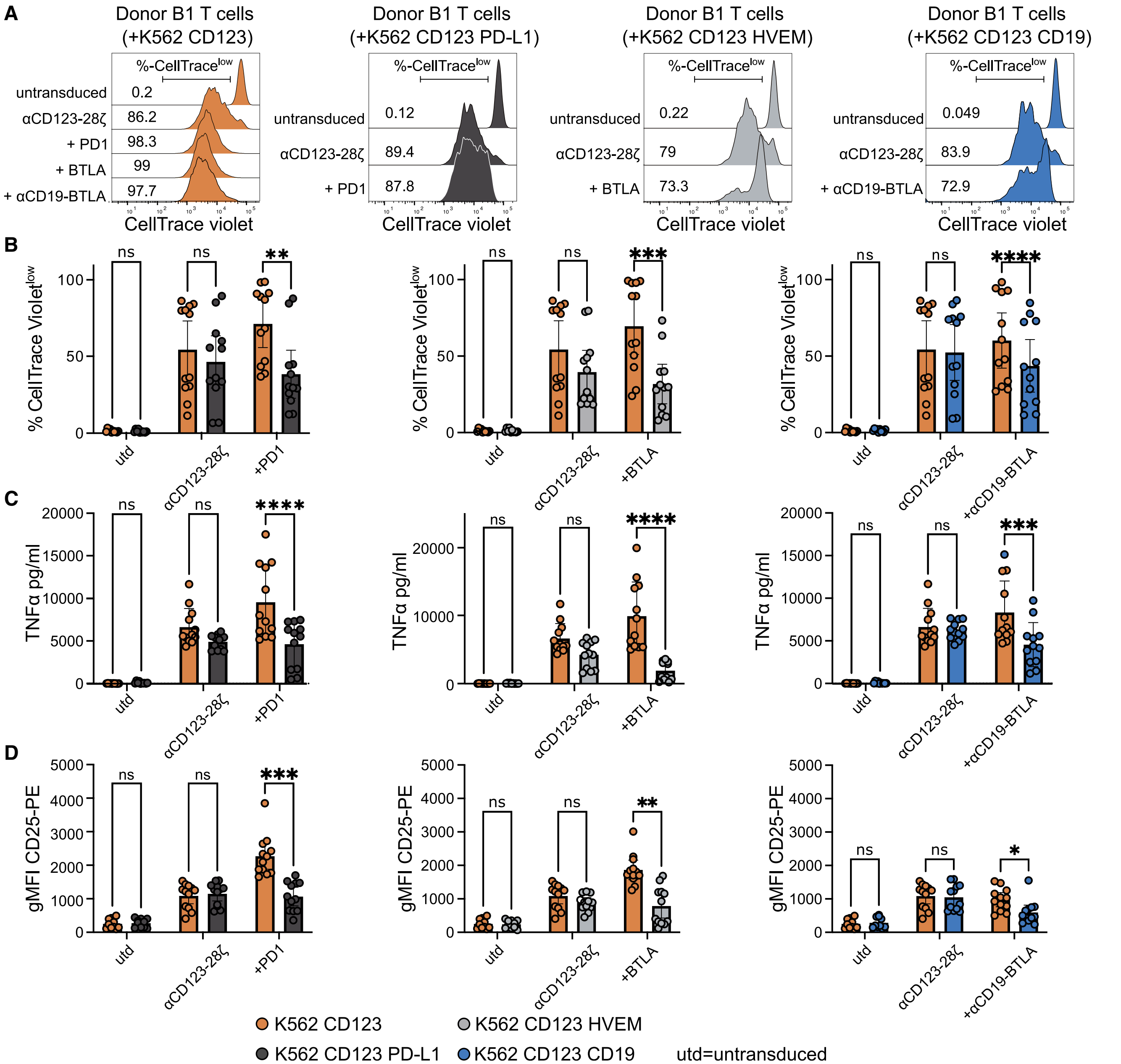

Figure 4. αCD19-BTLA iCAR acts by toning down transcriptionally dependent T cell effector functions

Untransduced T cell or αCD123-28ζ CAR-T cells without or with the indicated checkpoint/iCAR molecules were labeled with CellTrace Violet proliferation dye and cocultured with target cells (K562 CD123, orange; K562 CD123 PD-L1, dark gray; K562 CD123 HVEM, light gray; K562 CD123 CD19, blue). After 4 days of coculture, cells were stained with a CD25-PE antibody and analyzed by flow cytometry. Culture supernatants were analyzed for cytokine concentration (n = 6 donors, untransduced n = 5, each assay in duplicate). (A) Representative CellTrace Violet histograms of T cells from one donor. (B) Percentage of CellTrace Violet^{low} T cells. (C) Concentration of TNFα (pg/mL) in culture supernatants. (D) gMFI of T cells after staining with CD25-PE antibody. (B–D) Pooled data from all donors are presented as bar graphs (mean with 95% CI, dots represent individual repeats) comparing proliferation, TNFα, and expression of CD25 of the indicated T cells induced by K562 CD123 (orange) to K562 CD123 PD-L1 (dark gray), HVEM (light gray), or CD19 (blue). Mixed effects analysis with Šidák's multiple comparisons test was used to detect differences between the effect induced by K562 CD123 and the effect by the indicated other cell lines (*p < 0.05; **p < 0.01; ***p < 0.001; ****p < 0.0001; ns, not significant).

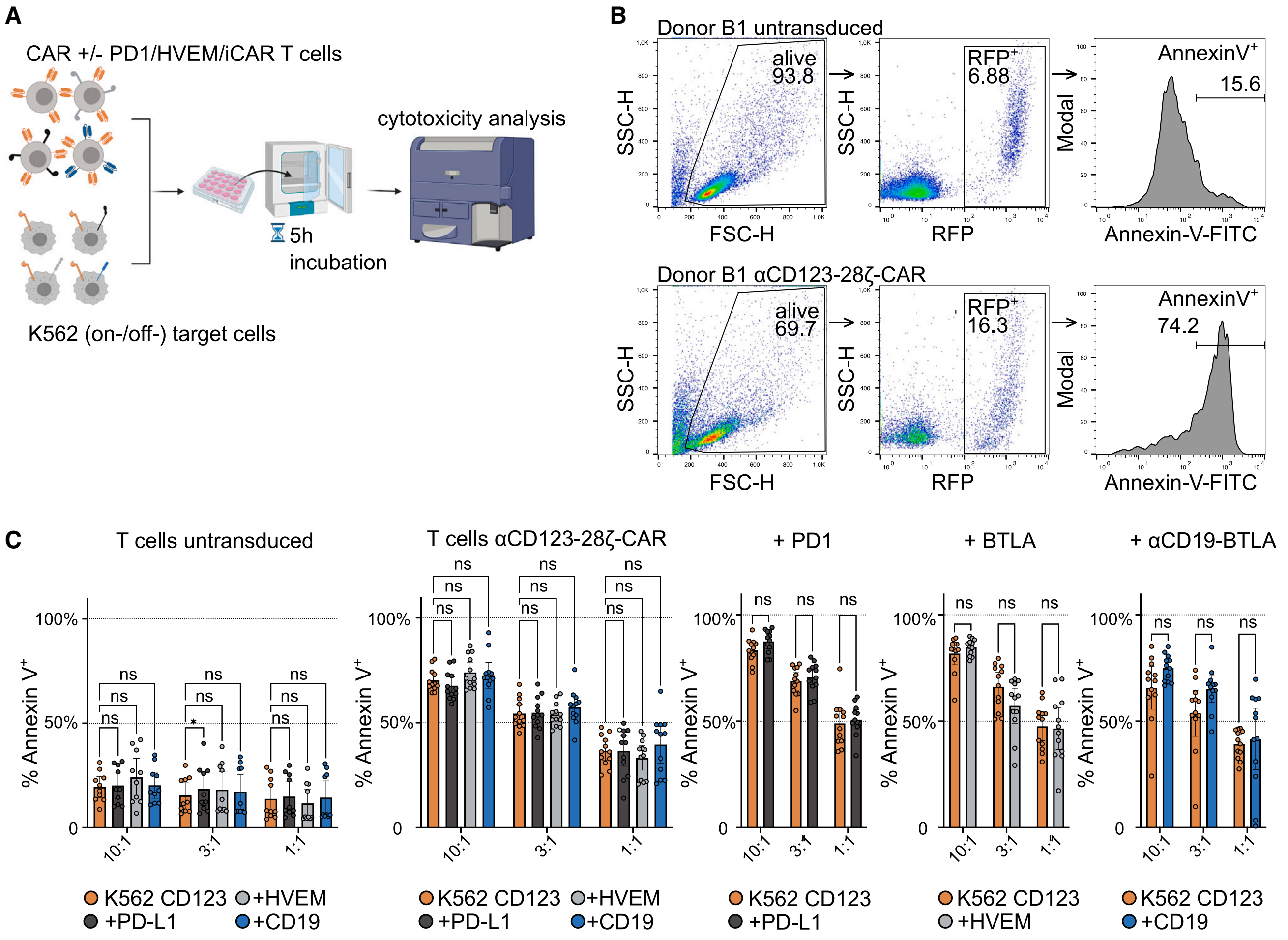

Figure 5. αCD19-BTLA iCAR does not prevent cytotoxicity by primary αCD123-CAR-T cells *in vitro*

(A) Scheme depicting the setup of CAR-T cell cytotoxicity assay. Untransduced and CAR-T cells were cocultured with K562 target cells expressing CD123 alone or in combination with an inhibitory ligand (PD-L1; HVEM or CD19) for 5 h. Subsequently, cells were stained with Annexin V-FITC and apoptotic target cells were identified by flow cytometry. (B) Gating strategy for the flow cytometry-based cytotoxicity assay. Target cells were identified by FSC-H/SSC-H gating (left) and RFP positivity (middle). Apoptotic target cells were identified by Annexin V-FITC positivity (right). (C) Untransduced T cells or αCD123-CAR-T cells without or with additional expression of PD-1, BTLA, or an αCD19-BTLA iCAR were cocultured with target cells at an E:T ratio of 10:1, 3:1, and 1:1 (n = 6 donors, untransduced n = 5, each assay in duplicate). Each panel represents killing capacity by the indicated T cell condition. Different colored bars represent K562 CD123 (orange), K562 CD123 PD-L1 (dark gray), K562 CD123 HVEM (light gray), and K562 CD123 CD19 (blue) target cells. Killing is measured as percentage of Annexin V$^+$ target cells. Data are presented as mean with 95% CI. Dots represent individual repeats. Within each panel, differences between groups were analyzed by two-way RM ANOVA with Šidák's multiple comparisons test (ns, not significant).

cells were cocultured with the target cell lines for 5 h and subsequently cells were stained with Annexin V and evaluated by flow cytometry. Annexin V positivity of RFP$^+$ cells was defined as surrogate marker for cytotoxic activity (Figures 5A and 5B). As expected, αCD123-28ζ transduced T cells killed K562 CD123 target cell lines markedly better than untransduced control T cells (Figure 5C, left two panels). Importantly, additional expression of iCAR, PD-1, or BTLA did not impair killing of K562 CD123 cells (Figure 5C, orange bars). Surprisingly, αCD123-28ζ/PD-1 and αCD123-28ζ/BTLA CAR-T cells did not kill PD-L1$^+$ and HVEM$^+$ target cells less efficiently than PD-L1$^-$ and HVEM$^-$ target cells, respectively. Similarly, the coexpression of the αCD19-BTLA iCAR on αCD123-28ζ CAR-T cells did not mediate protection of CD19$^+$ target cells (Figure 5C, right three panels). Notably, there actually was a tendency toward augmented cytotoxicity when both CAR and iCAR were engaged. This could be due to enhanced killing caused by increased CAR-T cell-target cell interaction upon coengagement of αCD19-BTLA iCAR.

In the next step, we addressed whether the presence of the inhibitory ligands had a protective effect, granting a selection advantage to inhibitory ligand-expressing target cells over target cells lacking the inhibitory ligand in direct coculture experiments. For this, we used CD123$^+$ GFP$^+$ cells mixed in a 1:1 ratio with the RFP$^+$ target cells co-expressing CD123 and PD-L1, HVEM, or CD19. In these experiments, a protective effect would lead to an increased ratio of apoptotic GFP$^+$ to RFP$^+$ target cells determined by Annexin V positivity

(Figures S12A–S12C). To ensure that killing occurred, the rates of Annexin V positivity of RFP^+ and GFP^+ cells were first assessed separately. It was found that all cell lines were killed at comparable rates (Figures S12D and S12E). As shown in Figure S12F, the presence of CD19 or HVEM on the RFP^+ target cells did not increase this ratio, indicating that the expression of the inhibitory receptor BTLA or αCD19-iCAR did not reduce cytotoxic effects of CAR-T cells toward target cells expressing the respective ligand. Target cells expressing PD-L1 were less efficiently killed by αCD123-/PD-1 CAR-T cells but this effect was also observed with CAR-T cells not overexpressing PD-1 and might thus indicate that these cells were intrinsically less prone to cytotoxic effects independent of PD-L1 (Figure S12F).

To further characterize the influence and mechanism of checkpoint molecules on T cell-mediated cytotoxicity, we analyzed the effect of PD-1 and BTLA signaling on the degranulation marker CD107a. For this, PBMCs were activated *in vitro* to induce effector cell differentiation and placed in coculture with K562-based target cells that expressed a membrane bound anti-CD3-scFv (K562-S) (Figure S13A). Additional expression on PD-L1 and HVEM on K562-S did not affect upregulation of the degranulation marker CD107a (Figures S13B and S13C). As previously observed, parallel cocultures, however, revealed significantly decreased secretion of IL-2 and GM-CSF (Figures S13D and S13E).

Taken together, these results suggest that neither iCAR nor classical checkpoint receptors are capable of delivering a killing-veto signal to CAR-T cells in a short-term *in vitro* killing assay, a phenomenon that has been observed by other groups previously but conflicts with the general perspective on how iCARs and checkpoint receptors prevent off-tumor T cell activation.[30,35]

Limited efficacy of αCD19-iCAR-T cells to prevent cytotoxicity in long-term *in vitro* assays

To further investigate the interplay of immediate cytotoxicity and transcriptionally dependent T cell effector functions, we set up a long-term coculture assay at suboptimal effector to target (E:T) ratios to ensure target cell persistence. Untransduced, CAR- and CAR/iCAR-T cells were cocultured with K562 CD123 or K562 CD123 CD19 for 72 h. Then, we analyzed cytotoxicity by determining the count of remaining live target cells using flow cytometry. Additionally, culture supernatants were analyzed for IFNγ and TNFα (Figures 6A and 6B).

As expected, there was an E:T ratio-dependent reduction in target cells after 72 h in cocultures with αCD123-28ζ CAR-T cells or αCD123-28ζ αCD19-BTLA CAR/iCAR-T cells but not with untransduced T cells. Also, in these experiments we did not obtain evidence for a reduced cytotoxic activity of T cells expressing the αCD19-BTLA iCAR toward target cells coexpressing CD19 (Figures 6C and 6D). Cytokine secretion, however, was reduced in conditions where the αCD19-BTLA iCAR was engaged, corroborating our previous results (Figures 6E and 6F). These results show that, while iCAR engagement efficiently suppressed cytokine production, it failed to decrease cytotoxicity by the primed CAR/iCAR-T cells toward target cells. This dissociation hints at a differential regulation of T cell cytotoxicity and other effector functions. Next, we asked if there would be a selective advantage for iCAR-antigen-expressing target cells in a head-to-head comparison to iCAR-antigen-negative target cells. To achieve matching of CD123 expression, we expressed GFP in the K562 CD123 target cells, thereby creating three distinguishable target cell lines, matched in CD123 expression (K562 GFP^+ CD123, K562 GFP^- CD123, K562 GFP^- CD123 CD19). The GFP^+ cells and either one of the GFP^- cell lines were mixed 1:1 and set in coculture with untransduced T cells and CAR- or CAR/iCAR-T cells. As in the previous experiment, we analyzed cytotoxicity and cytokine release after 72 h (Figures S14A and S1B). For each target cell line, an E:T-ratio-dependent reduction of target cell numbers was observed in cocultures with T cells expressing the αCD123-28ζ alone or in combination with the αCD19-BTLA CAR (Figures S14C and S14D). Surprisingly, in these experiments the cytokine release was not reduced upon iCAR engagement. This may indicate that the presence of target cells lacking the iCAR antigen is able to overcome the inhibitory effects of target cells expressing the iCAR ligand (Figures S14E and S14F). When comparing the counts of GFP^+ to GFP^- cells after 72 h of coculture, we noticed that the ratio of GFP^-/GFP^+ cells was skewed toward iCAR-ligand-positive cells after cocultures with iCAR-expressing CAR-T cells at the highest E:T ratio. This was not observed in cocultures with lower numbers of effector cells (Figures S14G and S14H). This result may imply that, despite the massive initial "hit" that is not adverted by the iCAR, there still may be a slight selective advantage of iCAR-ligand-expressing cells under condition of prolonged CAR/iCAR-T cell exposure and high E:T ratio.

DISCUSSION

Inhibitory CAR-based NOT gating of CAR-T cells has been proposed to link CAR-T cell activation to specific antigen combinations rather than single antigens.[72,73] In this study, we set out to create powerful iCARs for clinically relevant disease models. To identify suitable signaling domains, we used signaling-domain chimera of PD-1 in a well-established Jurkat reporter cell line that can easily be adapted for high-throughput evaluation of different receptor constructs.[74] With this method, we were able to identify BTLA as the most efficient inhibitory signaling domain among our test set, a result that is in agreement with a study by Cho and colleagues that also showed that cytoplasmic sequence of BTLA can transduce potent inhibitory signals into CAR-T cells.[35] BTLA inhibitory signaling suppressed both NF-κB and NFAT and tuned down activation in reporter cells receiving CD28-mediated costimulation. In a next step, we reviewed literature to identify potential OTOT problems that may limit the translation of CAR-T cells to clinical testing. We identified two potential applications for iCARs, namely CD45-mediated protection of hematopoietic cells in the context of B7-H3 CAR-T cell therapy and CD19-mediated protection of an engineered hematopoietic stem-cell graft to allow for αCD123-CAR-T cell persistence instead of a mere bridge-to-transplant application in AML and BPDCN. While some groups have investigated the off-tumor expression pattern of B7-H3 studying αB7H3 CAR-T cells, these experiments often rely

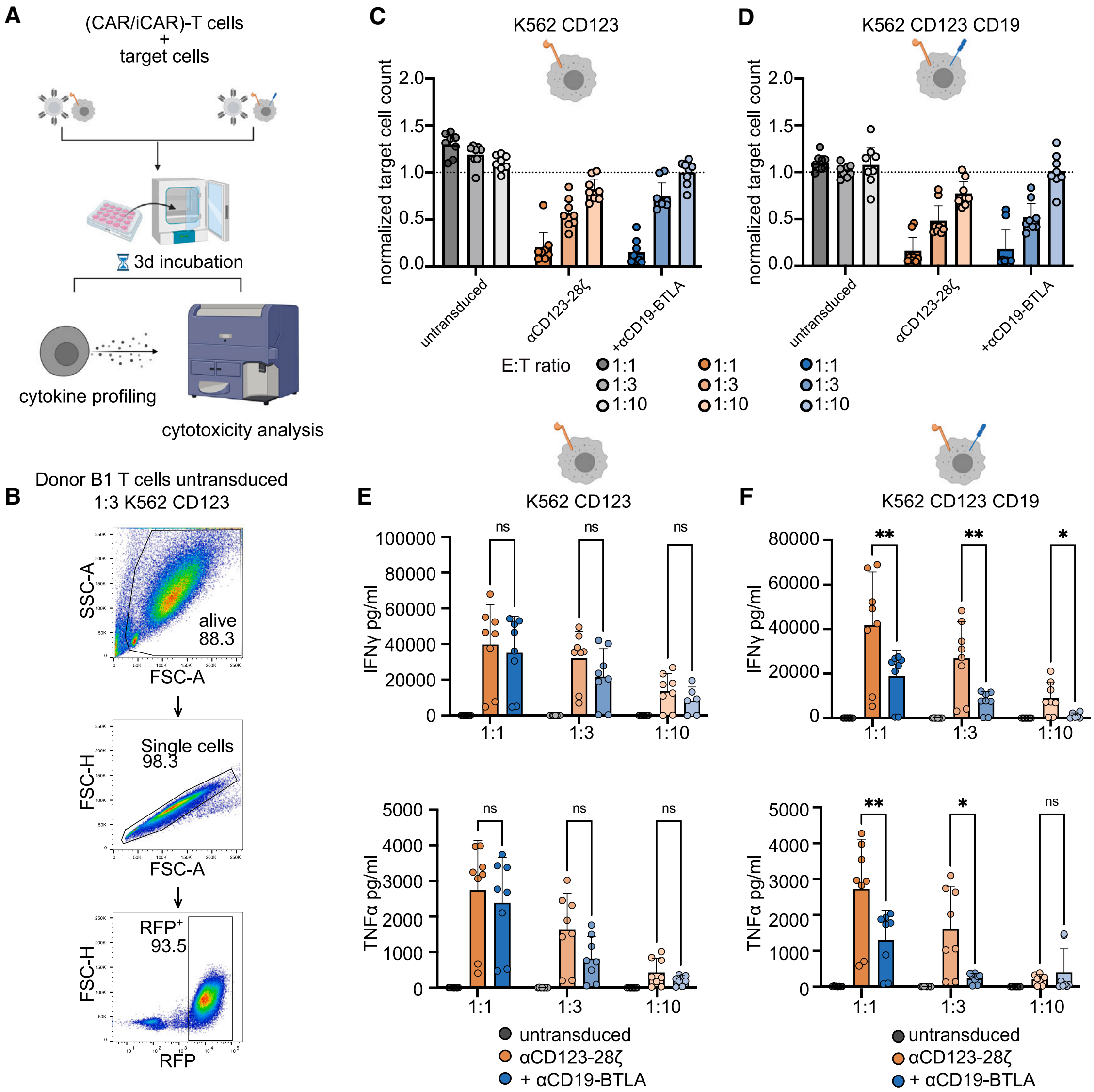

Figure 6. Analysis of iCAR effect on killing and cytokine release in long-term cocultures

(A) Scheme depicting the assay setup of CAR-T cell long-term coculture assay. Untransduced T cells or αCD123-28ζ CAR-T cells with or without αCD19-BTLA were cocultured for 72 h with either K562 CD123 or K562 CD123 CD19 target cells. Subsequently, cytotoxicity and cytokine concentrations were assessed (n = 4, all conditions in duplicate). (B) Gating strategy to assess target cell counts in coculture. (C and D) The count of remaining K562 CD123 (C) and K562 CD123 CD19 (D) under the indicated coculture conditions (E:T ratio, untransduced T cells, CAR-T cells ± iCAR). Target cell counts were normalized to target cell count without T cells. (E and F) Concentration of IFNγ (top) and TNFα (bottom) under the indicated coculture conditions. K562 CD123 (E) and K562 CD123 CD19 (F) were used as target cells. IFNγ data from one donor was excluded as outlier. For statistical analysis, mixed effect model (REML) with Tukey's multiple comparisons test (top) or two-way RM ANOVA with Tukey's multiple comparisons test (bottom) with donor-specific matching was performed (*$p < 0.05$; **$p < 0.01$; ns, not significant).

on immunohistochemistry of tissue where single B7-H3-expressing cells may be hard to detect, especially because B7-H3 expression is context dependent. An αCD45-based iCAR could be useful to protect such comparably rare, yet important, immune cell subsets.[60,61]

The feasibility of combined adoptive transfer of donor CAR-T cells and edited HSCs for therapy of AML has been demonstrated previously.[75] This approach was safe and effective but is limited to target antigens whose deletion does not impair functionality of hematopoietic cell development. Introduction of an inert safety ligand such as CD19 may therefore expand the scope of targetable antigens if sufficient inhibitory potency of the iCAR can be achieved. Reanalysis of public scRNA-seq datasets confirmed the expected antigen expression patterns in primary human cancer material. Again, we applied our versatile reporter cell system to test the potential αB7H3/αCD45 and αCD123/αCD19 CAR/iCAR combinations. We were

able to show significant inhibition of CAR-activated reporter cells by the respective iCAR molecules. The inhibitory effect of the iCARs was apparent for all transcription factors evaluated (NF-κB, NFAT, AP1), which indicates that iCARs interfere with membrane proximal signaling processes. We chose the αCD123-28ζ/αCD19-BTLA CAR/iCAR combination for exploratory evaluation in primary T cells to validate our reporter cell results. As expected, we found an inhibitory effect of the αCD19-BTLA iCAR on key T cell effector functions, namely proliferation, cytokine release, and upregulation of the activation-induced marker CD25. This underscores the usefulness of the reporter cell system as a tool to study transcriptionally dependent T cell effector functions. Surprisingly, however, we did not observe a protective effect of the αCD19-BTLA iCAR in a cytotoxicity assay conducted in parallel. There was even a slight tendency toward enhanced killing, possibly due to an increased avidity of the T cell-target cell interaction resulting from the additional antigen binding by the iCAR. We observed similar "adhesion" effects in conditions where signaling-deficient PD-1 chimera and Δcyt-CARs were engaged in addition to an activating stimulus. At the same time, our experiments with a titratable X15-scTCR-iCAR model showed antigen dose dependency of inhibitory signaling by iCARs. To create a functional iCAR, these two factors will have to be balanced carefully. iCARs targeting low abundance antigens would therefore need a particularly strong inhibitory signaling domain.

While there are reports that iCARs have the potential to reduce cytotoxicity toward target cells coexpressing the iCAR antigen,[30,33] Cho et al. also reported that inhibitory zipCAR signaling reduced CAR-mediated cytokine production in human CD4 and CD8 T cells but failed to reduce cytotoxicity in CD8 T cells.[35] Importantly, we observed that the presence of the inhibitory ligands PD-L1 and HVEM on target cells resulted in diminished CAR-T cell proliferation and cytokine production but failed to protect them from cytotoxicity even when the respective inhibitory receptors were strongly overexpressed in the CAR-T cells. PD-1 differentially regulates T cell functions depending on the activation threshold required for their induction.[76] This may also apply for inhibitory receptors with similar molecular mechanism of action, such as BTLA and the iCAR in this study. A lower activation threshold for cytotoxicity compared to transcriptionally dependent effector functions is likely to be the mechanism behind the observed differential response to inhibitory receptors. In this regard, primed CTLs and, likewise, CAR-T cells differ from other cytotoxic immune effector cells such as NK cells. Target cell killing by NK cells is well known to be vetoed by signals of inhibitory receptors and NK cells could be effective as CAR transduced effector cells for off-the-shelf cancer immunotherapy.[77] Importantly, Cho and colleagues demonstrated that an inhibitory zipCAR that failed to reduce cytotoxic activity of CD8 T cells was efficient in NK cells.[35] The inhibitory zipCAR used in their study also harbored an intracellular domain derived from BTLA, suggesting differential effects of inhibitory signaling in these cell types. Altogether, these results indicate that, in CTLs, including CAR-T cells, cytotoxicity is hardwired to be refractory to inhibitory signaling. Protective effects of iCARs were reported in studies where long-term cytotoxicity assays were performed. Over this extended assay duration, other effects such as proliferation and cytokine-mediated enhancement of cytotoxicity may play a confounding role, which makes it difficult to evaluate cytotoxicity exclusively. We observed a slight advantage of iCAR-ligand-expressing target cells over control target cells in a 3-day coculture assay. However, this was only observed at a certain effector-target cell ratio and the large majority of iCAR-ligand-expressing target cells were not spared also under these conditions. Previously, *in vivo* experiments have demonstrated that tumors expressing an iCAR ligand are capable of outgrowing a tumor lacking the respective antigen.[30] However, in our view, these experiments are confounded in that mice do not vitally rely on the injected tumor cells. Considering the lack of protection against immediate cytotoxicity observed in our study, *in vivo* experiments would have to use models where the animals depend on the OTOT cell population to perform its intended function, as success of an iCAR approach would heavily rely on the initial reservoir and regenerative potential of the OTOT cells.

In conclusion, our study identifies BTLA as a potent signaling domain for iCARs and explores feasibility of clinically relevant CAR/iCAR combinations. Importantly, we show that the tested iCARs and checkpoint receptors are unable to provide a strict veto signal to mediate protection against OTOT-killing but rather affect transcriptionally regulated T cell effector functions. Consequently, OTOT leakiness may remain with every methodology that relies on iCARs harboring signaling domains derived from inhibitory receptors in T cells and this should be considered when translating such approaches into clinical applications.

MATERIALS AND METHODS

Healthy donor PBMCs

Healthy donor, human PBMCs were acquired from the Department of Blood Group Serology and Transfusion Medicine of the Vienna General Hospital (Vienna, Austria) or the Vienna Red Cross (Vienna Red Cross) after obtaining written informed consent. PBMC isolation was performed by standard density-gradient centrifugation with Lymphoprep solution (Technoclone, Vienna, Austria). The study was conducted under approval by the ethics committee of the Medical University of Vienna under the registration number 1772/2017 and in accordance to the Helsinki Declaration of 1964 and its later amendments.

Cell lines, media, reagents, and flow cytometry

All reporter cell experiments were performed using the well-established triple-parameter-reporter cell line JE6.1 TPR.[36] In some experiments, TCS cells, which are based on the cell line BW5147 and express a membrane-bound anti-CD3-scF$_v$ with or without coexpression of PD-L1, were used as described elsewhere.[45] In all experiments involving CARs and iCARs, K562 cells were used as stimulator cells. The K562 cells were engineered to express the indicated antigen molecules and the fluorescent proteins RFP657 (short RFP), eGFP, or both. In some assays, K562 cells expressing the membrane-bound anti-CD3-scF$_v$ with or without coexpression of PD-L1 or HVEM were used as stimulator cells (K562-S, K562-S PD-L1, K562-S

Table 1. Antibodies and reagents used for staining and flow cytometric assessment of cells

Name	Clone	Supplier
B7-H3-PE	MIH42	BioLegend
BTLA-APC	J168-540	BD Bioscience
CD107a-FITC	H4.A3	BioLegend
CD123-PE	6H6	BioLegend
CD19-PE	HIB19	BioLegend
CD25-PE	M-A251	BioLegend
CD4-BV421	OKT4	BioLegend
CD45-PE	2D1	BioLegend
CD8-PerCP	HIT8a	BioLegend
HVEM-PE	122	BioLegend
mCD45.2-APC	104	BioLegend
PD-L1-PE	29E.2A3	BioLegend
PD-1-APC	EH12.2H7	BioLegend
SA-FITC	–	BioLegend
SA-PE	–	BioLegend
Strep-II-tag-biotin	5A9F9	GenScript, NJ, USA
V5-tag	TCM5	Thermo Fisher Scientific

HVEM). For plasmid transfection and production of retro- or lentiviral supernatants, HEK293T cells were used. TPR, TCS, and K562 cells were cultured in RPMI 1640 medium supplemented with 100 μg/mL streptomycin, 100 U/mL penicillin, and 10% heat-inactivated fetal calf serum (Thermo Fisher Scientific, MA, USA) at 37°C and 5% CO_2. For cells transduced with plasmids with a puromycin-resistance gene, the medium was supplemented with 1 μg/mL puromycin. HEK293T cells were cultured in Iscove's Modified Dulbecco's Medium (IMDM) medium supplemented analogously to the RPMI 1640 medium and 2 mM L-glutamine. All cell lines were regularly tested for mycoplasma contamination using a reporter cell-based assay developed by Battin et al.[78] Surface expression of all molecules was confirmed by flow cytometry using the antibodies listed in Table 1. Flow cytometric measurements were performed on a FACSCalibur with CellQuest software or a LSRFortessa with FACSDiva software (both BD Bioscience, NJ, USA). For some cell lines and all primary T cells, flow sorting was performed using a Sony SH800 sorter (Sony Biotechnology, CA, USA). Final analysis of the flow cytometry data was done using the FlowJo 10.8.2 software (Becton Dickinson, NJ, USA).

Retroviral and lentiviral transduction

All molecules described were expressed in cell lines or T cells using retroviral or lentiviral expression. For retroviral transduction of stimulator cells, sequences encoding PD-L1 (UniProt: Q9NZQ7-1), B7-H3 (UniProt Q5ZPR3), and HVEM (UniProt: Q92956-1) were cloned into the pBMN vector and CD123 (UniProt: P26951-1), CD45 (UniProt: P08575-3), and CD19 (UniProt: P15391-1) were cloned into the pCJK2 vector.[45] All other constructs were cloned into a lentiviral vector (pHR-Puro) based on the previously described vector pHR-SIN-BX-IRES-Emerald.[79] In this vector, a P2A ribosomal-skipping sequence followed by a gene encoding puromycin-N-acetyl-transferase is added to the expressed molecule, which allows puromycin selection of transduced cells. For the PD-1-GFP and BTLA-GFP (BTLA: UniProt Q7Z6A9-1, R157S, and P267L natural variant, T212A) constructs the puromycin-resistance gene was exchanged for eGFP to enable flow sorting of transduced cells. TPR were engineered to express PD-1 (UniProt Q15116) or chimera consisting of PD-1 extracellular domain (UniProt Q15116 amino acids [aa] 1–170, S34F sequence conflict), a CD28 transmembrane domain (UniProt P10747 aa 153–179), and the signaling domains BTLA (UniProt Q7Z6A9-1 aa 179–289, T212A, P267L natural variant), LILRB1 (UniProt: Q8NHL6-1 aa 483–650), KIR2DL1: (UniProt P43626-1 aa 265–348), Δcyt (no signaling domain), short (UniProt Q15116 aa 192–220 + aa 246–288), 2xITSM (UniProt Q15116 aa 192–288, 221–226 VDYGEL->TEYATI), or +ITSM (UniProt Q15116 aa 192–253 + aa 227–253). The CAR extracellular domains consisted of a GM-CSF-R-alpha leader sequence (UniProt: P15509-1 aa 1–22) followed by the respective scF_v-sequence and a CD8-alpha chain hinge domain (UniProt: P01732-1 aa 139–178, C164S mutation to prevent dimerization). For cell surface detectability by flow cytometry, αCD123- and αB7H3-CARs or αCD45- and αCD19-iCARs were equipped with different protein tags 3′ of the scF_v sequence, a V5 tag (GKPIPNPLLGLDST), or a strep-II tag (NWSHPQFEK), respectively. Additionally, the αCD19 CAR had an myc tag ($(EQKLISEEDL)_2$) 5′ of the scF_v sequence. The αCD123- and αCD45-CARs are derived from the therapeutics flotetuzumab (MacroGenics, MD, USA; IMGT/monoclonal antibody [mAb]-DB: 679) and apamistamab (Actinium Pharmaceuticals, NY, USA; IMGT/mAb-DB: 633), respectively. Sequences for V_H and V_L chains were obtained from the IMGT-mAb database.[80,] V_H and V_L sequences for the αB7H3 CAR are based on the mAb 376.96 and were obtained from patent literature.[66,81] To create scF_vs, the V_L chains were linked to the V_H chains by a $(G_4S)_3$ linker. The scF_v of the αCD19-iCAR was taken from the well-established αCD19-CAR used in the CAR-T cell product axicabtagene ciloleucel (Gilead, CA, USA; IMGT/mAb-DB: 827). For the X15-construct extracellular domain, we used the X15-scTCR[53] followed by a strep-II tag and a CD8 alpha chain hinge. The scTCR/CAR/iCAR constructs further consisted of a CD28 transmembrane domain (UniProt P10747 aa 153–179) followed by the activating signaling domains ζ (UniProt: P20963-3 aa 52–163), BB-ζ (UniProt: Q07011-1 aa 214–255 + UnitProt: P20963-3 aa 52–163) or 28-ζ (UniProt: P10747-1 aa 180–220 + UniProt: P20963-3 aa 52–163), the inhibitory domains PD-1 (UniProt Q15116 aa 192–288) or BTLA (UniProt: Q7Z6A9-1 aa 179–289, T212A, P267L natural variant), a signaling-deficient domain (UniProt Q15116 aa 192–208, X15-Δcyt), or no signaling domain (αCD45-Δcyt, αCD19-Δcyt). Successful vector integration and sequence accuracy of all constructs was ensured by Sanger sequencing (Eurofins Genomics, Ebersberg, Germany). The protocol used for cell line transduction was previously described in detail.[79] In short, HEK293T cells were transfected by calcium-phosphate precipitation. Resulting virus supernatant was harvested after 48 h and

applied to the cell lines. Finally, stable expression of the respective molecules was confirmed by flow cytometric analysis. Production of primary human CAR-T cells is described separately in a later section.

CRISPR-Cas9

To create CD45^{-} target cells and prevent αCD45-iCAR triggering by CD45^{+} iCAR-reporter cells in coculture assays, CRISPR-Cas9-mediated knockout of *PTPRC* (CD45) was performed in K562 RFP, K562 RFP B7-H3, and JE6.1 TPR cell lines. Predesigned single guide RNA was acquired from Integrated DNA Technology (IDT, IO, USA) (5′-ACAACCACTCTGAGCCCTTC-3′). Together with tracrRNA and Cas9, a ribonucleoprotein complex was assembled and delivered to the cell lines by electroporation using the Neon Transfection system (Thermo Fisher Scientific, MA, USA) according to the manufacturer's protocol. To assess knockout success, we performed flow cytometric analysis of CD45 surface expression on transfected cells. Additionally, genomic DNA was isolated using the Gentra Puregene Cell Kit (Qiagen, Hilden, Germany). We then performed target-site amplification by PCR and Sanger sequencing of the resulting product (Eurofins Genomics). Knockout efficiency was then confirmed by applying the TIDE online tool (http://shinyapps.datacurators.nl/tide/).[82]

Validation of CAR/iCAR target antigens in publicly available transcriptomic datasets

A publicly available scRNA-seq and cellular indexing of transcriptomes and epitopes (CITE)-seq dataset[62] (GEO: GSE185381) of adult as well as pediatric AML patients and healthy controls was reanalyzed to determine the expression pattern of *CD19* (CD19) and *IL3RA* (CD123) across various cell types. Data were filtered, doublet removed, normalized, processed, and visualized using scanpy and the scvi-tools.[83,84] Healthy control cells were clustered, projected as uniform manifold approximation and projection (UMAP), and assigned to certain cell types defined by marker gene expression. Then, expression of *CD19* (CD19) and *IL3RA* (CD123) was analyzed. To differentiate malignant cells from microenvironmental cells in single-cell data of AML patients, the annotation provided in the datasets' metadata on the Single Cell Portal (accessed under https://singlecell.broadinstitute.org/single_cell, Broad Institute, MA, USA) was used and analysis of expression of *CD19* (CD19) and *IL3RA* (CD123) was visualized using the Single Cell Portal online tool. To analyze expression patterns of *PTPRC* (CD45) and *CD276* (B7-H3) in malignant versus healthy cells in primary human glioblastoma, glioma, and melanoma samples, publicly available scRNA-seq datasets (GEO: GSE131928,[63] GEO: GSE182109[64]; European Genome Phenome Archive Dataset ID: EGAS00001005115, sample M-P1[65]) were accessed through the Single Cell Portal. Cell type annotations were taken over from the authors as deposited. Expression of *PTPRC* (CD45) and *CD276* (B7-H3) was then analyzed by searching the respective genes and displaying their expression using the Single Cell Portal online tool.

Reporter cell assays

For reporter assays, 5×10^4 TPR reporter cells were cocultured with 2×10^4 stimulator cells (TCS or K562) in a 96-well flat-bottom plate at 37°C and 5% CO_2. After 18–24 h, cells were harvested; washed in fluorescence-activated cell sorting (FACS) buffer (PBS, 0.5% FCS and 0.01% sodium azide); and, if TCS were used, an mCD45.2-APC antibody was added to allow gating on reporter cells. K562 could be excluded from analysis by expression of RFP. After washing and staining, the cells were analyzed by flow cytometry. Reporter cell (mCD45.2^{-} or RFP^{-} cells) activation was expressed as geometric mean fluorescence intensity (gMFI) of the reporter genes NF-κB-eCFP, NFAT-eGFP, and AP1-mCherry. Full stimulation was defined as gMFI in presence of an activating ligand without an inhibitory ligand. To calculate inhibition by PD-1-chimera or iCARs, the following formula was used:

$$\frac{(\text{gMFI with inhibitory ligand} - \text{gMFI unstimulated}) \times 100}{(\text{gMFI full stimulation} - \text{gMFI unstimulated})}$$

Inhibition is therefore presented as percentage of full stimulation reached in presence of an inhibitory ligand (PD-L1 or iCAR antigen).

CAR-T cell production from healthy donor PBMCs

To produce CAR-T cells, T cells were isolated from healthy donor PBMCs using a pan-T cell isolation kit (Miltenyi Biotec, Bergisch Gladbach, Germany). T cells were resuspended in supplemented RPMI medium containing IL-2 (50 IU/mL, Peprotech, NJ, USA) and anti-CD3 (OKT3, Ortho Pharmaceutical Corporation, NJ, USA) and anti-CD28 (clone 28.2, BioLegend)-coated g'a'm-Dynabeads (Thermo Fisher Scientific) were added in a 1:1 ratio. The T cell/bead suspension was then plated in 24-well plates at a concentration of 1×10^6 T cells/mL and incubated for 48 h at 37°C and 5% CO_2. For production of lentivirus, HEK293T cells were transfected using Lipofectamine 3000 (Thermo Fisher Scientific) according to the manufacturer's protocol. Supernatant was harvested after 24 h and directly added to activated T cells or frozen at −80°C for later use. 500 μL of each supernatant was added to the T cells (final dilution 1:3 or 1:4, αCD123-28ζ CAR only or αCD123-28ζ CAR + inhibitory receptor/iCAR). Dynabeads were removed 1–2 days after transduction and transduced T cells were expanded in supplemented RPMI medium with addition of IL-7 and IL-15 (both 10 ng/mL, both Peprotech) for 11–12 days. To enrich desired transduced cells, T cells were sorted for high expression of cell surface receptors (αCD123-CAR, V5-tag positive; PD-1/BTLA, GFP positive; or αCD19-iCAR, strep-II tag positive) during the expansion phase. Prior to further use, CAR-T cells were analyzed by flow cytometry to determine expression of molecules of interest and to determine CD4:CD8 ratio of final CAR-T cell product.

Annexin V cytotoxicity assay

To determine cytotoxicity by CAR/iCAR-T cells, 10×10^4, 3×10^4, or 1×10^4 T cells were added to 1×10^4 target cells (E:T ratio 10:1, 3:1, or 1:1) in 96-well round-bottom plates. For assays with two distinct target cell populations, the same amount of T cells was added to 1×10^4 target cells each (E:T 10:1:1, 3:1:1, 1:1:1). Coculture was maintained for 5 h at 37°C and 5% CO_2. Next, cells were harvested,

transferred to 1.2-mL microtiter tubes, and washed with FACS buffer. Cells were then pelleted by centrifugation and resuspended in 50 μL of Annexin V binding buffer (BioLegend). Annexin V-FITC for single target cell assays or Annexin V-PE for dual-target-cell assays (both BioLegend) was diluted 1:100 from stock, 5 μL were added to each tube, and cells were incubated for 15 min in the dark at room temperature. Finally, another 50 μL of Annexin V binding buffer was added to a total volume of 105 μL. Cells were then analyzed by flow cytometry. Flow cytometry analysis was performed using constant cell volumes, flow rates, and acquisition time for all samples to detect target cell loss as described recently.[85] Cytotoxicity was measured as percentage of Annexin V-positive target cells of all target cells.

Determining T cell proliferation and upregulation of CD25

Prior to coculture, T cells were stained with CellTrace Violet proliferation dye (Thermo Fisher Scientific). To exclude overgrowth, target cells were treated with Mitomycin C (Carl Roth, Karlsruhe, Germany).[86] Then 10×10^4 T cells were cocultured with 4×10^4 target cells (E:T 5:2) in 200 μL of fresh, supplemented RPMI medium for 4 days in 96-well round-bottom plates. Next, culture supernatant was harvested and frozen at −20°C for later analysis. Cells were washed and stained with CD25-PE antibody (BioLegend). Proliferation was expressed as percentage of CellTrace Violet^{low} T cells. Upregulation of CD25-PE was defined as increase in CD25-PE gMFI of T cells.

Cytokine profiling

As outlined, cell culture supernatants from proliferation assays were harvested after 4 days of coculture and were frozen at −20°C to preserve cytokines for later analysis. IFNγ, TNFα, IL-2, and GM-CSF were measured using HCYTOMAG-60K (IFNγ) and HCD8MAG-15K (other cytokines) kits (both Merck, Darmstadt, Germany) on the Luminex 100 system (Luminex, TX, USA).

CD107a staining assay

To measure degranulation via CD107a staining, PBMCs were cultured in supplemented RPMI medium containing IL-2 (50 IU/mL, Peprotech, NJ, USA) and anti-CD3 (OKT3, Ortho Pharmaceutical Corporation, NJ, USA) and anti-CD28 (clone 28.2, BioLegend)-coated g'a'm-Dynabeads (Thermo Fisher Scientific) were added in a 1:1 ratio. T cells were cultured for 48 h in 24-well plates at a concentration of 1×10^6 cells/mL. Subsequently, 1×10^5 PBMCs were cocultured with K562-S, K562-S-PD-L1, and K562-S HVEM (2×10^4) for 5 h (total volume, 100 μL/well). An anti-CD107a antibody (H4.A3, FITC labeled, BioLegend) was added together with Golgistop and Golgiplug (BD Bioscience) at the onset of coculture. Subsequently, cells were stained for expression of CD4 and CD8 (CD4-BV421, CD8-PerCP, both BioLegend) and analyzed by flow cytometry. Sample acquisition was performed using constant volumes, flow rates, and acquisition time for all samples. Luminex-based cytokine analyses were performed on a parallel coculture after 24–48 h as described above.

Combined cytotoxicity and cytokine profiling assay

Untransduced T cells or CAR-T cells with or without iCAR were cocultured with 3×10^4 target cells at E:T ratios of 1:1, 1:3, and 1:10. In the mixed on-/on- and on-/off-target cell assays, 1.5×10^4 GFP$^+$ and GFP$^-$ target cells were mixed (E:T ratio of 1:0.5:0.5, 1:1.5:1.5, and 1:5:5). After 72 h, culture supernatants were harvested and cytokine analysis was performed. Cells were then washed and resuspended in 100 μL of FACS buffer. To determine cytotoxicity, a constant volume of cells was taken up on the LSRFortessa flow cytometer by setting a fixed uptake time at constant flow (30s, medium flow). Target cell counts were normalized to target cell cultures without addition of T cells to correct for potential cell-line-intrinsic differences in proliferation.

Statistical analysis

All statistical analyses were performed using Prism 9 (GraphPad Software, MA, USA). In comparisons comprising cells from multiple donors stimulated under varying conditions, matching for the individual donor was performed. To compare one variable in two groups, Mann Whitney U test for unpaired, non-normally distributed data or paired t test for matched, normally distributed data were used. For comparison of one variable in multiple groups, Brown-Forsythe and Welch ANOVA with Dunnett's T3 multiple comparisons test or Kruskal-Wallis test with Dunn's multiple comparisons test was used for unmatched data and repeated measurements (RM) one-way ANOVA or mixed effects analysis with Šidák's multiple comparisons test or Friedman test with Dunn's multiple comparisons test was applied for matched datasets. If data were analyzed with respect to two variables, either two-way ANOVA or two-way RM ANOVA was used, both accompanied by either Šidák's or Tukey's multiple comparisons test. In one case, an incomplete dataset was analyzed using a mixed-effects model restricted maximum likelihood (REML) with Tukey's multiple comparisons test. To test for normal distribution of data, the Shapiro-Wilk test was applied. Statistical significance was assumed at two-tailed p values <0.05. The statistical test performed in the respective figure is designated in the figure legend.

Schemes

All schemes were created using the BioRender (Toronto, Canada) platform.

DATA AND CODE AVAILABILITY

scRNA-seq datasets analyzed in this study can be accessed at GEO (GEO: GSE185381, GSE131928, and GSE182109) or through the European Genome Phenome Archive (Dataset ID: EGAS00001005115, sample M-P1). Some visualization (Figures 2C–2F and S3) can be directly accessed through the Single Cell Portal. Other data will be provided through the corresponding author upon request.

SUPPLEMENTAL INFORMATION

Supplemental information can be found online at https://doi.org/10.1016/j.ymthe.2024.02.022.

ACKNOWLEDGMENTS

This study was supported by a grant by the City of Vienna Fund for Innovative Interdisciplinary Cancer Research to M.F. (22025/2021) and a grant of the Austrian Science Fund (FWF-P32411) to P.S. We wish to thank Claus Wenhardt and Simon Gumpelmair for technical assistance.

AUTHOR CONTRIBUTIONS

M.F. performed the experiments, designed the study, analyzed data, and wrote the manuscript. G.H. reanalyzed publicly available scRNA-seq datasets. P.W.-S. performed cytokine measurements. J.L. isolated PBMCs from healthy donors, performed the CD107a degranulation assays, and provided critical support with all primary T cell experiments. P.S. designed and supervised the study and wrote the manuscript. All authors critically revised the manuscript and approved the final version.

DECLARATION OF INTERESTS

The authors declare no competing interests.

REFERENCES

1. Flugel, C.L., Majzner, R.G., Krenciute, G., Dotti, G., Riddell, S.R., Wagner, D.L., and Abou-el-Enein, M. (2023). Overcoming on-target, off-tumour toxicity of CAR T cell therapy for solid tumours. Nat. Rev. Clin. Oncol. *20*, 49–62. https://doi.org/10.1038/s41571-022-00704-3.

2. Larson, R.C., and Maus, M.V. (2021). Recent advances and discoveries in the mechanisms and functions of CAR T cells. Nat. Rev. Cancer *21*, 145–161. https://doi.org/10.1038/s41568-020-00323-z.

3. Wat, J., and Barmettler, S. (2022). Hypogammaglobulinemia After Chimeric Antigen Receptor (CAR) T-Cell Therapy: Characteristics, Management, and Future Directions. J. Allergy Clin. Immunol. Pract. *10*, 460–466. https://doi.org/10.1016/j.jaip.2021.10.037.

4. Lamers, C.H.J., Klaver, Y., Gratama, J.W., Sleijfer, S., and Debets, R. (2016). Treatment of metastatic renal cell carcinoma (mRCC) with CAIX CAR-engineered T-cells - A completed study overview. Biochem. Soc. Trans. *44*, 951–959. https://doi.org/10.1042/BST20160037.

5. Thistlethwaite, F.C., Gilham, D.E., Guest, R.D., Rothwell, D.G., Pillai, M., Burt, D.J., Byatte, A.J., Kirillova, N., Valle, J.W., Sharma, S.K., et al. (2017). The clinical efficacy of first-generation carcinoembryonic antigen (CEACAM5)-specific CAR T cells is limited by poor persistence and transient pre-conditioning-dependent respiratory toxicity. Cancer Immunol. Immunother. *66*, 1425–1436. https://doi.org/10.1007/s00262-017-2034-7.

6. Morgan, R.A., Yang, J.C., Kitano, M., Dudley, M.E., Laurencot, C.M., and Rosenberg, S.A. (2010). Case Report of a Serious Adverse Event Following the Administration of T Cells Transduced With a Chimeric Antigen Receptor Recognizing Erbb2. Mol. Ther. *18*, 843–851. https://doi.org/10.1038/mt.2010.24.

7. Qi, C., Gong, J., Li, J., Liu, D., Qin, Y., Ge, S., Zhang, M., Peng, Z., Zhou, J., Cao, Y., et al. (2022). Claudin18.2-specific CAR T cells in gastrointestinal cancers: phase 1 trial interim results. Nat. Med. *28*, 1189–1198. https://doi.org/10.1038/s41591-022-01800-8.

8. Hou, A.J., Chen, L.C., and Chen, Y.Y. (2021). Navigating CAR-T cells through the solid-tumour microenvironment. Nat. Rev. Drug Discov. *20*, 531–550. https://doi.org/10.1038/s41573-021-00189-2.

9. Parker, K.R., Migliorini, D., Perkey, E., Yost, K.E., Bhaduri, A., Bagga, P., Haris, M., Wilson, N.E., Liu, F., Gabunia, K., et al. (2020). Single-Cell Analyses Identify Brain Mural Cells Expressing CD19 as Potential Off-Tumor Targets for CAR-T Immunotherapies. Cell *183*, 126–142.e17. https://doi.org/10.1016/j.cell.2020.08.022.

10. Cameron, B.J., Gerry, A.B., Dukes, J., Harper, J.V., Kannan, V., Bianchi, F.C., Grand, F., Brewer, J.E., Gupta, M., Plesa, G., et al. (2013). Identification of a Titin-derived HLA-A1-presented peptide as a cross-reactive target for engineered MAGE A3-directed T cells. Sci. Transl. Med. *5*, 197ra103. https://doi.org/10.1126/scitranslmed.3006034.

11. Donnadieu, E., Luu, M., Alb, M., Anliker, B., Arcangeli, S., Bonini, C., De Angelis, B., Choudhary, R., Espie, D., Galy, A., et al. (2022). Time to evolve: predicting engineered T cell-associated toxicity with next-generation models. J. Immunother. Cancer *10*, e003486. https://doi.org/10.1136/jitc-2021-003486.

12. Bueno, C., Velasco-Hernandez, T., Gutiérrez-Agüera, F., Zanetti, S.R., Baroni, M.L., Sánchez-Martínez, D., Molina, O., Closa, A., Agraz-Doblás, A., Marín, P., et al. (2019). CD133-directed CAR T-cells for MLL leukemia: on-target, off-tumor myeloablative toxicity. Leukemia *33*, 2090–2125. https://doi.org/10.1038/s41375-019-0418-8.

13. Richman, S.A., Nunez-Cruz, S., Moghimi, B., Li, L.Z., Gershenson, Z.T., Mourelatos, Z., Barrett, D.M., Grupp, S.A., and Milone, M.C. (2018). High-Affinity GD2-specific CAR T cells induce fatal encephalitis in a preclinical neuroblastoma model. Cancer Immunol. Res. *6*, 36–46. https://doi.org/10.1158/2326-6066.CIR-17-0211.

14. Majzner, R.G., Theruvath, J.L., Nellan, A., Heitzeneder, S., Cui, Y., Mount, C.W., Rietberg, S.P., Linde, M.H., Xu, P., Rota, C., et al. (2019). CAR T Cells Targeting B7-H3, a Pan-Cancer Antigen, Demonstrate Potent Preclinical Activity Against Pediatric Solid Tumors and Brain Tumors. Clin. Cancer Res. *25*, 2560–2574. https://doi.org/10.1158/1078-0432.CCR-18-0432.

15. Baroni, M.L., Sanchez Martinez, D., Gutierrez Aguera, F., Roca Ho, H., Castella, M., Zanetti, S.R., Velasco Hernandez, T., Diaz de la Guardia, R., Castaño, J., Anguita, E., et al. (2020). 41BB-based and CD28-based CD123-redirected T-cells ablate human normal hematopoiesis in vivo. J. Immunother. Cancer *8*, e000845. https://doi.org/10.1136/jitc-2020-000845.

16. Singh, N., and Maus, M.V. (2023). Synthetic manipulation of the cancer-immunity cycle: CAR-T cell therapy. Immunity *56*, 2296–2310. https://doi.org/10.1016/j.immuni.2023.09.010.

17. Wilkie, S., van Schalkwyk, M.C.I., Hobbs, S., Davies, D.M., van der Stegen, S.J.C., Pereira, A.C.P., Burbridge, S.E., Box, C., Eccles, S.A., and Maher, J. (2012). Dual Targeting of ErbB2 and MUC1 in Breast Cancer Using Chimeric Antigen Receptors Engineered to Provide Complementary Signaling. J. Clin. Immunol. *32*, 1059–1070. https://doi.org/10.1007/s10875-012-9689-9.

18. Kloss, C.C., Condomines, M., Cartellieri, M., Bachmann, M., and Sadelain, M. (2013). Combinatorial antigen recognition with balanced signaling promotes selective tumor eradication by engineered T cells. Nat. Biotechnol. *31*, 71–75. https://doi.org/10.1038/nbt.2459.

19. Zhang, E., Yang, P., Gu, J., Wu, H., Chi, X., Liu, C., Wang, Y., Xue, J., Qi, W., Sun, Q., et al. (2018). Recombination of a dual-CAR-modified T lymphocyte to accurately eliminate pancreatic malignancy. J. Hematol. Oncol. *11*, 102. https://doi.org/10.1186/s13045-018-0646-9.

20. Roybal, K.T., Rupp, L.J., Morsut, L., Walker, W.J., McNally, K.A., Park, J.S., and Lim, W.A. (2016). Precision Tumor Recognition by T Cells With Combinatorial Antigen-Sensing Circuits. Cell *164*, 770–779. https://doi.org/10.1016/J.CELL.2016.01.011.

21. Morsut, L., Roybal, K.T., Xiong, X., Gordley, R.M., Coyle, S.M., Thomson, M., and Lim, W.A. (2016). Engineering Customized Cell Sensing and Response Behaviors Using Synthetic Notch Receptors. Cell *164*, 780–791. https://doi.org/10.1016/J.CELL.2016.01.012.

22. Srivastava, S., Salter, A.I., Liggitt, D., Yechan-Gunja, S., Sarvothama, M., Cooper, K., Smythe, K.S., Dudakov, J.A., Pierce, R.H., Rader, C., and Riddell, S.R. (2019). Logic-Gated ROR1 Chimeric Antigen Receptor Expression Rescues T Cell-Mediated Toxicity to Normal Tissues and Enables Selective Tumor Targeting. Cancer Cell *35*, 489–503.e8. https://doi.org/10.1016/j.ccell.2019.02.003.

23. Moghimi, B., Muthugounder, S., Jambon, S., Tibbetts, R., Hung, L., Bassiri, H., Hogarty, M.D., Barrett, D.M., Shimada, H., and Asgharzadeh, S. (2021). Preclinical assessment of the efficacy and specificity of GD2-B7H3 SynNotch CAR-T in metastatic neuroblastoma. Nat. Commun. *12*, 511. https://doi.org/10.1038/s41467-020-20785-x.

24. Salzer, B., Schueller, C.M., Zajc, C.U., Peters, T., Schoeber, M.A., Kovacic, B., Buri, M.C., Lobner, E., Dushek, O., Huppa, J.B., et al. (2020). Engineering AvidCARs for combinatorial antigen recognition and reversible control of CAR function. Nat. Commun. *11*, 4166. https://doi.org/10.1038/s41467-020-17970-3.

25. Juillerat, A., Marechal, A., Filhol, J.M., Valogne, Y., Valton, J., Duclert, A., Duchateau, P., and Poirot, L. (2017). An oxygen sensitive self-decision making engineered CAR T-cell. Sci. Rep. *7*, 39833. https://doi.org/10.1038/srep39833.

26. Kosti, P., Opzoomer, J.W., Larios-Martinez, K.I., Henley-Smith, R., Scudamore, C.L., Okesola, M., Taher, M.Y.M., Davies, D.M., Muliaditan, T., Larcombe-Young, D., et al. (2021). Hypoxia-sensing CAR T cells provide safety and efficacy in treating solid tumors. Cell Rep. Med. *2*, 100227. https://doi.org/10.1016/j.xcrm.2021.100227.

27. Choe, J.H., Watchmaker, P.B., Simic, M.S., Gilbert, R.D., Li, A.W., Krasnow, N.A., Downey, K.M., Yu, W., Carrera, D.A., Celli, A., et al. (2021). SynNotch-CAR T cells overcome challenges of specificity, heterogeneity, and persistence in treating glioblastoma. Sci. Transl. Med. *13*, eabe7378. https://doi.org/10.1126/scitranslmed.abe7378.

28. Han, X., Bryson, P.D., Zhao, Y., Cinay, G.E., Li, S., Guo, Y., Siriwon, N., and Wang, P. (2017). Masked Chimeric Antigen Receptor for Tumor-Specific Activation. Mol. Ther. *25*, 274–284. https://doi.org/10.1016/j.ymthe.2016.10.011.

29. Ellis, G.I., Sheppard, N.C., and Riley, J.L. (2021). Genetic engineering of T cells for immunotherapy. Nat. Rev. Genet. *22*, 427–447. https://doi.org/10.1038/s41576-021-00329-9.

30. Fedorov, V.D., Themeli, M., and Sadelain, M. (2013). PD-1- and CTLA-4-based inhibitory chimeric antigen receptors (iCARs) divert off-target immunotherapy responses. Sci. Transl. Med. *5*, 215ra172. https://doi.org/10.1126/scitranslmed.3006597.

31. Sahillioglu, A.C., Toebes, M., Apriamashvili, G., Gomez, R., and Schumacher, T.N. (2021). Crash-it switch enables reversible and dose-dependent control of tcr and car t-cell function. Cancer Immunol. Res. *9*, 999–1007. https://doi.org/10.1158/2326-6066.CIR-21-0095.

32. Richards, R.M., Zhao, F., Freitas, K.A., Parker, K.R., Xu, P., Fan, A., Sotillo, E., Daugaard, M., Oo, H.Z., Liu, J., et al. (2021). NOT-Gated CD93 CAR T Cells Effectively Target AML with Minimized Endothelial Cross-Reactivity. Blood Cancer Discov. *2*, 648–665. https://doi.org/10.1158/2643-3230.BCD-20-0208.

33. Hamburger, A.E., DiAndreth, B., Cui, J., Daris, M.E., Munguia, M.L., Deshmukh, K., Mock, J.Y., Asuelime, G.E., Lim, E.D., Kreke, M.R., et al. (2020). Engineered T cells directed at tumors with defined allelic loss. Mol. Immunol. *128*, 298–310. https://doi.org/10.1016/J.MOLIMM.2020.09.012.

34. Tokatlian, T., Asuelime, G.E., Mock, J.-Y., DiAndreth, B., Sharma, S., Toledo Warshaviak, D., Daris, M.E., Bolanos, K., Luna, B.L., Naradikian, M.S., et al. (2022). Mesothelin-specific CAR-T cell therapy that incorporates an HLA-gated safety mechanism selectively kills tumor cells. J. Immunother. Cancer *10*, e003826. https://doi.org/10.1136/JITC-2021-003826.

35. Cho, J.H., Okuma, A., Sofjan, K., Lee, S., Collins, J.J., and Wong, W.W. (2021). Engineering advanced logic and distributed computing in human CAR immune cells. Nat. Commun. *12*, 792. https://doi.org/10.1038/s41467-021-21078-7.

36. Jutz, S., Leitner, J., Schmetterer, K., Doel-Perez, I., Majdic, O., Grabmeier-Pfistershammer, K., Paster, W., Huppa, J.B., and Steinberger, P. (2016). Assessment of costimulation and coinhibition in a triple parameter T cell reporter line: Simultaneous measurement of NF-κB, NFAT and AP-1. J. Immunol. Methods *430*, 10–20. https://doi.org/10.1016/j.jim.2016.01.007.

37. Liu, S., Galat, V., Galat, Y., Lee, Y.K.A., Wainwright, D., and Wu, J. (2021). NK cell-based cancer immunotherapy: from basic biology to clinical development. J. Hematol. Oncol. *14*, 7. https://doi.org/10.1186/s13045-020-01014-w.

38. Borrego, F., Kabat, J., Kim, D.-K., Lieto, L., Maasho, K., Peña, J., Solana, R., Coligan, J.E., Pena, J., Solana, R., et al. (2002). Structure and function of major histocompatibility complex (MHC) class I specific receptors expressed on human natural killer (NK) cells. Mol. Immunol. *38*, 637–660. https://doi.org/10.1016/s0161-5890(01)00107-9.

39. Qureshi, O.S., Zheng, Y., Nakamura, K., Attridge, K., Manzotti, C., Schmidt, E.M., Baker, J., Jeffery, L.E., Kaur, S., Briggs, Z., et al. (2011). Trans-endocytosis of CD80 and CD86: a molecular basis for the cell-extrinsic function of CTLA-4. Science *332*, 600–603. https://doi.org/10.1126/science.1202947.

40. Kennedy, A., Waters, E., Rowshanravan, B., Hinze, C., Williams, C., Janman, D., Fox, T.A., Booth, C., Pesenacker, A.M., Halliday, N., et al. (2022). Differences in CD80 and CD86 transendocytosis reveal CD86 as a key target for CTLA-4 immune regulation. Nat. Immunol. *23*, 1365–1378. https://doi.org/10.1038/s41590-022-01289-w.

41. Rosskopf, S., Leitner, J., Zlabinger, G.J., and Steinberger, P. (2019). CTLA-4 antibody ipilimumab negatively affects CD4+ T-cell responses in vitro. Cancer Immunol. Immunother. *68*, 1359–1368. https://doi.org/10.1007/S00262-019-02369-X.

42. Walker, L.S.K., and Sansom, D.M. (2011). The emerging role of CTLA4 as a cell-extrinsic regulator of T cell responses. Nat. Rev. Immunol. *11*, 852–863. https://doi.org/10.1038/nri3108.

43. De Sousa Linhares, A., Leitner, J., Grabmeier-Pfistershammer, K., and Steinberger, P. (2018). Not All Immune Checkpoints Are Created Equal. Front. Immunol. *9*, 1909. https://doi.org/10.3389/fimmu.2018.01909.

44. Hudecek, M., Lupo-Stanghellini, M.T., Kosasih, P.L., Sommermeyer, D., Jensen, M.C., Rader, C., and Riddell, S.R. (2013). Receptor affinity and extracellular domain modifications affect tumor recognition by ROR1-specific chimeric antigen receptor T cells. Clin. Cancer Res. *19*, 3153–3164. https://doi.org/10.1158/1078-0432.CCR-13-0330.

45. Leitner, J., Kuschei, W., Grabmeier-Pfistershammer, K., Woitek, R., Kriehuber, E., Majdic, O., Zlabinger, G., Pickl, W.F., and Steinberger, P. (2010). T cell stimulator cells, an efficient and versatile cellular system to assess the role of costimulatory ligands in the activation of human T cells. J. Immunol. Methods *362*, 131–141. https://doi.org/10.1016/j.jim.2010.09.020.

46. Patsoukis, N., Duke-Cohan, J.S., Chaudhri, A., Aksoylar, H.I., Wang, Q., Council, A., Berg, A., Freeman, G.J., and Boussiotis, V.A. (2020). Interaction of SHP-2 SH2 domains with PD-1 ITSM induces PD-1 dimerization and SHP-2 activation. Commun. Biol. *3*, 128. https://doi.org/10.1038/s42003-020-0845-0.

47. Okazaki, T., Maeda, A., Nishimura, H., Kurosaki, T., and Honjo, T. (2001). PD-1 immunoreceptor inhibits B cell receptor-mediated signaling by recruiting src homology 2-domain-containing tyrosine phosphatase 2 to phosphotyrosine. Proc. Natl. Acad. Sci. USA *98*, 13866–13871. https://doi.org/10.1073/pnas.231486598.

48. Chemnitz, J.M., Parry, R.V., Nichols, K.E., June, C.H., and Riley, J.L. (2004). SHP-1 and SHP-2 Associate with Immunoreceptor Tyrosine-Based Switch Motif of Programmed Death 1 upon Primary Human T Cell Stimulation, but Only Receptor Ligation Prevents T Cell Activation. J. Immunol. *173*, 945–954. https://doi.org/10.4049/jimmunol.173.2.945.

49. Hombach, A., Sent, D., Schneider, C., Heuser, C., Koch, D., Pohl, C., Seliger, B., and Abken, H. (2001). T-cell activation by recombinant receptors: CD28 costimulation is required for interleukin 2 secretion and receptor-mediated T-cell proliferation but does not affect receptor-mediated target cell lysis. Cancer Res. *61*, 1976–1982.

50. Chmielewski, M., Hombach, A.A., and Abken, H. (2011). CD28 cosignalling does not affect the activation threshold in a chimeric antigen receptor-redirected T-cell attack. Gene Ther. *18*, 62–72. https://doi.org/10.1038/gt.2010.127.

51. Chen, L., and Flies, D.B. (2013). Molecular mechanisms of T cell co-stimulation and co-inhibition. Nat. Rev. Immunol. *13*, 227–242. https://doi.org/10.1038/nri3405.

52. Van Der Stegen, S.J.C., Hamieh, M., and Sadelain, M. (2015). The pharmacology of second-generation chimeric antigen receptors. Nat. Rev. Drug Discov. *14*, 499–509. https://doi.org/10.1038/nrd4597.

53. Aggen, D.H., Chervin, A.S., Insaidoo, F.K., Piepenbrink, K.H., Baker, B.M., and Kranz, D.M. (2011). Identification and engineering of human variable regions that allow expression of stable single-chain T cell receptors. Protein Eng. Des. Sel. *24*, 361–372. https://doi.org/10.1093/PROTEIN/GZQ113.

54. Valent, P., Sadovnik, I., Eisenwort, G., Bauer, K., Herrmann, H., Gleixner, K.V., Schulenburg, A., Rabitsch, W., Sperr, W.R., and Wolf, D. (2019). Immunotherapy-Based Targeting and Elimination of Leukemic Stem Cells in AML and CML. Int. J. Mol. Sci. *20*, 4233. https://doi.org/10.3390/ijms20174233.

55. Testa, U., Pelosi, E., and Castelli, G. (2019). CD123 as a Therapeutic Target in the Treatment of Hematological Malignancies. Cancers (Basel) *11*, 1358. https://doi.org/10.3390/cancers11091358.

56. Bôle-Richard, E., Fredon, M., Biichlé, S., Anna, F., Certoux, J.M., Renosi, F., Tsé, F., Molimard, C., Valmary-Degano, S., Jenvrin, A., et al. (2020). CD28/4-1BB CD123 CAR T cells in blastic plasmacytoid dendritic cell neoplasm. Leukemia *34*, 3228–3241. https://doi.org/10.1038/s41375-020-0777-1.

57. Goldsmith, S.R., Ghobadi, A., and DiPersio, J.F. (2020). Hematopoeitic Cell Transplantation and CAR T-Cell Therapy: Complements or Competitors? Front. Oncol. *10*, 608916. https://doi.org/10.3389/FONC.2020.608916.

58. Maude, S.L., Frey, N., Shaw, P.A., Aplenc, R., Barrett, D.M., Bunin, N.J., Chew, A., Gonzalez, V.E., Zheng, Z., Lacey, S.F., et al. (2014). Chimeric Antigen Receptor T Cells for Sustained Remissions in Leukemia. N. Engl. J. Med. *371*, 1507–1517. https://doi.org/10.1056/NEJMOA1407222.

59. Du, H., Hirabayashi, K., Ahn, S., Kren, N.P., Montgomery, S.A., Wang, X., Tiruthani, K., Mirlekar, B., Michaud, D., Greene, K., et al. (2019). Antitumor Responses in the Absence of Toxicity in Solid Tumors by Targeting B7-H3 via Chimeric Antigen Receptor T Cells. Cancer Cell *35*, 221–237.e8. https://doi.org/10.1016/j.ccell.2019.01.002.

60. Steinberger, P., Majdic, O., Derdak, S.V., Pfistershammer, K., Kirchberger, S., Klauser, C., Zlabinger, G., Pickl, W.F., Stöckl, J., and Knapp, W. (2004). Molecular Characterization of Human 4Ig-B7-H3, a Member of the B7 Family with Four Ig-Like Domains. J. Immunol. *172*, 2352–2359. https://doi.org/10.4049/JIMMUNOL.172.4.2352.

61. Chapoval, A.I., Ni, J., Lau, J.S., Wilcox, R.A., Flies, D.B., Liu, D., Dong, H., Sica, G.L., Zhu, G., Tamada, K., and Chen, L. (2001). B7-H3:A costimulatory molecule for T cell activation and IFN-γ production. Nat. Immunol. *2*, 269–274. https://doi.org/10.1038/85339.

62. Lasry, A., Nadorp, B., Fornerod, M., Nicolet, D., Wu, H., Walker, C.J., Sun, Z., Witkowski, M.T., Tikhonova, A.N., Guillamot-Ruano, M., et al. (2023). An inflammatory state remodels the immune microenvironment and improves risk stratification in acute myeloid leukemia. Nat. Cancer *4*, 27–42. https://doi.org/10.1038/s43018-022-00480-0.

63. Neftel, C., Laffy, J., Filbin, M.G., Hara, T., Shore, M.E., Rahme, G.J., Richman, A.R., Silverbush, D., Shaw, M.L., Hebert, C.M., et al. (2019). An Integrative Model of Cellular States, Plasticity, and Genetics for Glioblastoma. Cell *178*, 835–849.e21. https://doi.org/10.1016/J.CELL.2019.06.024.

64. Abdelfattah, N., Kumar, P., Wang, C., Leu, J.S., Flynn, W.F., Gao, R., Baskin, D.S., Pichumani, K., Ijare, O.B., Wood, S.L., et al. (2022). Single-cell analysis of human glioma and immune cells identifies S100A4 as an immunotherapy target. Nat. Commun. *13*, 767. https://doi.org/10.1038/s41467-022-28372-y.

65. Wu, S.Z., Roden, D.L., Al-Eryani, G., Bartonicek, N., Harvey, K., Cazet, A.S., Chan, C.L., Junankar, S., Hui, M.N., Millar, E.A., et al. (2021). Cryopreservation of human cancers conserves tumour heterogeneity for single-cell multi-omics analysis. Genome Med. *13*, 81. https://doi.org/10.1186/S13073-021-00885-Z.

66. Dotti, G., Ferrone, S., Du, H., Wang, X., and Ferrone, C. (2019). Methods and Compositions for Chimeric Antigen Receptor Targeting Cancer Cells. US Patent US10233226.

67. Poiron, C., Wu, Y., Ginestoux, C., Ehrenmann, F., Duroux, P., and Lefranc, M.-P. (2010). IMGT/mAb-DB: The IMGT® Database for Therapeutic Monoclonal Antibodies. JOBIM 2010 conference *Abstract*, Paper 13.

68. Pagel, J.M., Appelbaum, F.R., Eary, J.F., Rajendran, J., Fisher, D.R., Gooley, T., Ruffner, K., Nemecek, E., Sickle, E., Durack, L., et al. (2006). 31 I-anti-CD45 antibody plus busulfan and cyclophosphamide before allogeneic hematopoietic cell transplantation for treatment of acute myeloid leukemia in first remission. Blood *107*, 2184–2191. https://doi.org/10.1182/blood-2005-06-2317.

69. Chichili, G.R., Huang, L., Li, H., Burke, S., He, L., Tang, Q., Jin, L., Gorlatov, S., Ciccarone, V., Chen, F., et al. (2015). A CD3xCD123 bispecific DART for redirecting host T cells to myelogenous leukemia: Preclinical activity and safety in nonhuman primates. Sci. Transl. Med. *7*, 289ra82. https://doi.org/10.1126/SCITRANSLMED.AAA5693.

70. Kochenderfer, J.N., Feldman, S.A., Zhao, Y., Xu, H., Black, M.A., Morgan, R.A., Wilson, W.H., and Rosenberg, S.A. (2009). Construction and Preclinical Evaluation of an Anti-CD19 Chimeric Antigen Receptor. J. Immunother. *32*, 689–702. https://doi.org/10.1097/CJI.0b013e3181ac6138.

71. Cappell, K.M., and Kochenderfer, J.N. (2021). A comparison of chimeric antigen receptors containing CD28 versus 4-1BB costimulatory domains. Nat. Rev. Clin. Oncol. *18*, 715–727. https://doi.org/10.1038/s41571-021-00530-z.

72. Savanur, M.A., Weinstein-marom, H., and Gross, G. (2021). Implementing Logic Gates for Safer Immunotherapy of Cancer. Front. Immunol. *12*, 780399. https://doi.org/10.3389/fimmu.2021.780399.

73. Han, X., Wang, Y., Wei, J., and Han, W. (2019). Multi-antigen-targeted chimeric antigen receptor T cells for cancer therapy. J. Hematol. Oncol. *12*, 128. https://doi.org/10.1186/s13045-019-0813-7.

74. Jutz, S., Hennig, A., Paster, W., Asrak, Ö., Dijanovic, D., Kellner, F., Pickl, W.F., Huppa, J.B., Leitner, J., Steinberger, P., et al. (2017). A cellular platform for the evaluation of immune checkpoint molecules. Oncotarget *8*, 64892–64906. https://doi.org/10.18632/oncotarget.17615.

75. Kim, M.Y., Yu, K.R., Kenderian, S.S., Ruella, M., Chen, S., Shin, T.H., Aljanahi, A.A., Schreeder, D., Klichinsky, M., Shestova, O., et al. (2018). Genetic Inactivation of CD33 in Hematopoietic Stem Cells to Enable CAR T Cell Immunotherapy for Acute Myeloid Leukemia. Cell *173*, 1439–1453.e19. https://doi.org/10.1016/j.cell.2018.05.013.

76. Shimizu, K., Sugiura, D., Okazaki, I.M., Maruhashi, T., Takegami, Y., Cheng, C., Ozaki, S., and Okazaki, T. (2020). PD-1 Imposes Qualitative Control of Cellular Transcriptomes in Response to T Cell Activation. Mol. Cell *77*, 937–950.e6. https://doi.org/10.1016/j.molcel.2019.12.012.

77. Nowak, J., Bentele, M., Kutle, I., Zimmermann, K., Lühmann, J.L., Steinemann, D., Kloess, S., Koehl, U., Roßberg, W., Ahmed, A., et al. (2023). CAR-NK Cells Targeting HER1 (EGFR) Show Efficient Anti-Tumor Activity against Head and Neck Squamous Cell Carcinoma (HNSCC). Cancers (Basel) *15*, 3169. https://doi.org/10.3390/cancers15123169.

78. Battin, C., Hennig, A., Mayrhofer, P., Kunert, R., Zlabinger, G.J., Steinberger, P., and Paster, W. (2017). A human monocytic NF-κB fluorescent reporter cell line for detection of microbial contaminants in biological samples. PLoS One *12*, e0178220. https://doi.org/10.1371/journal.pone.0178220.

79. Paster, W., Brockmeyer, C., Fu, G., Simister, P.C., de Wet, B., Martinez-Riaño, A., Hoerter, J.A.H., Feller, S.M., Wülfing, C., Gascoigne, N.R.J., and Acuto, O. (2013). GRB2-Mediated Recruitment of THEMIS to LAT Is Essential for Thymocyte Development. J. Immunol. *190*, 3749–3756. https://doi.org/10.4049/JIMMUNOL.1203389.

80. Cambon, M., Cherouali, K., Kushwaha, A., Giudicelli, V., Duroux, P., Kossida, S., and Lefranc, M.-P. (2018). IMGT/mAb-DB and IMGT/2Dstructure-DB for IMGT standard definition of an antibody: from receptor to amino acid changes pdf. JOBIM 2018 conference Abstract, Poster 201.

81. Imai, K., Wilson, B.S., Bigotti, A., Natali, P.G., and Ferrone, S. (1982). A 94,000-dalton glycoprotein expressed by human melanoma and carcinoma cells. J. Natl. Cancer Inst. *68*, 761–769.

82. Brinkman, E.K., Chen, T., Amendola, M., and Van Steensel, B. (2014). Easy quantitative assessment of genome editing by sequence trace decomposition. Nucleic Acids Res. *42*, e168. https://doi.org/10.1093/NAR/GKU936.

83. Wolf, F.A., Angerer, P., and Theis, F.J. (2018). SCANPY: large-scale single-cell gene expression data analysis. Genome Biol. *19*, 15. https://doi.org/10.1111/1462-2920.13787.

84. Gayoso, A., Lopez, R., Xing, G., Boyeau, P., Valiollah Pour Amiri, V., Hong, J., Wu, K., Jayasuriya, M., Mehlman, E., Langevin, M., et al. (2022). A Python library for probabilistic analysis of single-cell omics data. Nat. Biotechnol. *40*, 163–166. https://doi.org/10.1038/s41587-021-01206-w.

85. Leitner, J., Egerer, R., Waidhofer-Söllner, P., Grabmeier-Pfistershammer, K., and Steinberger, P. (2023). FcγR requirements and costimulatory capacity of Urelumab, Utomilumab, and Varlilumab. Front. Immunol. *14*, 1208631–1208714. https://doi.org/10.3389/fimmu.2023.1208631.

86. Battin, C., Kaufmann, G., Leitner, J., Tobias, J., Wiedermann, U., Rölle, A., Meyer, M., Momburg, F., and Steinberger, P. (2022). NKG2A-checkpoint inhibition and its blockade critically depends on peptides presented by its ligand HLA-E. Immunology *166*, 507–521. https://doi.org/10.1111/imm.13515.

YMTHE, Volume *32*

Supplemental Information

Inhibitory CARs fail to protect from immediate T cell cytotoxicity

Maximilian A. Funk, Gerwin Heller, Petra Waidhofer-Söllner, Judith Leitner, and Peter Steinberger

Supplemental Figures

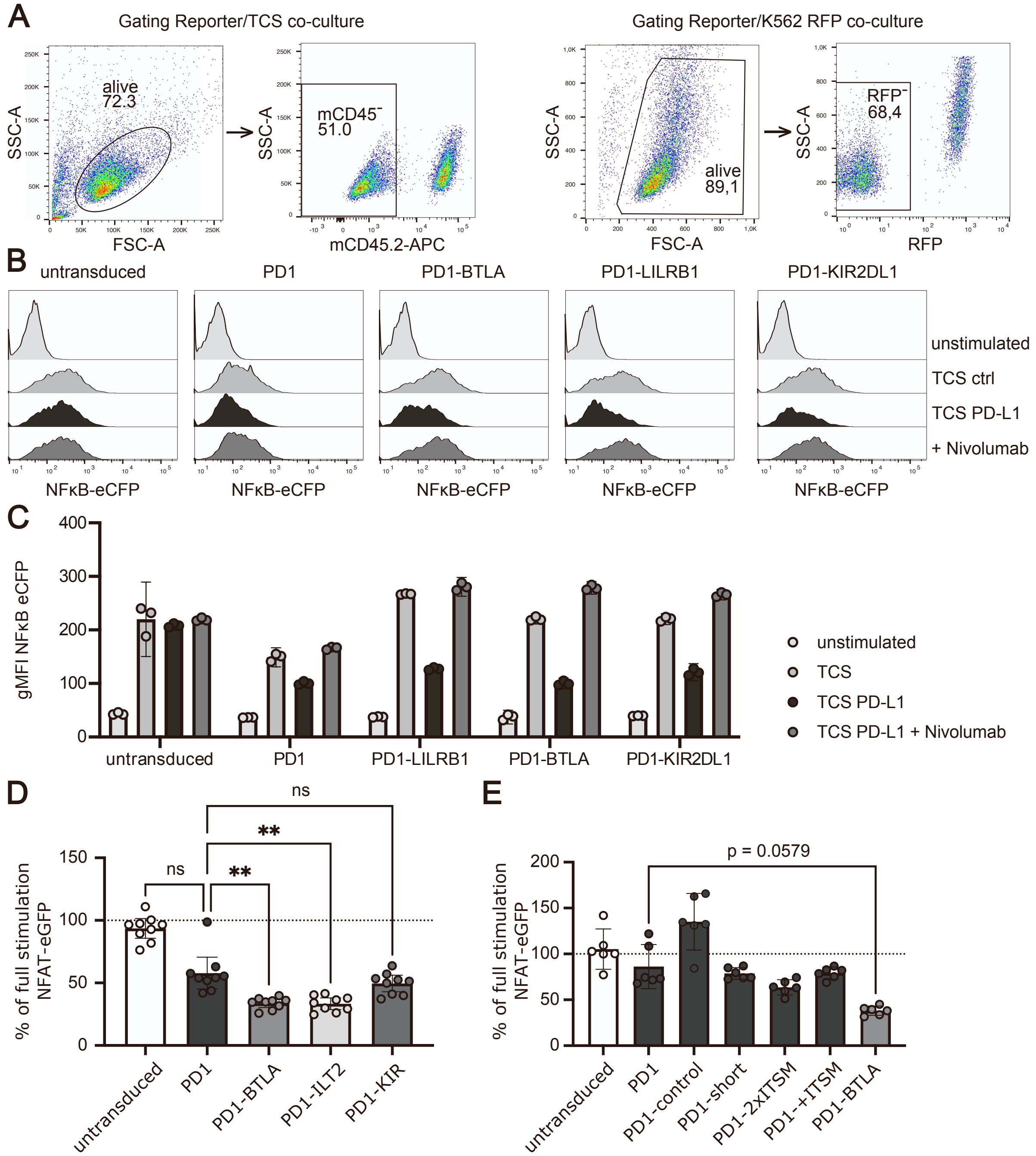

Fig. S1 **Supplemental information to Figure 1. A** Gating strategy to identify TPR cells in coculture assays with TCS cells that express murine CD45 (left) and K562 RFP (right). **B** Representative histograms showing induction of NFκB-eCFP reporter gene expression under the indicated stimulation conditions. **C** Representative assay showing reduced expression of the NFκB-eCFP reporter gene by TPR expressing PD1 or PD1-chimera if stimulated by TCS PD-L1 compared to stimulation with TCS. The effect is reversible by adding the PD1 blocking antibody Nivolumab (n=1, assay performed in triplicates). Data is presented as triplicate mean with 95% CI. Dots indicate individual repeats. **D** Analysis of inhibition of the NFAT-eGFP reporter gene in coculture assay described in Fig. 1D. **E** Analysis of inhibition of the NFAT-eGFP reporter gene in coculture assay described in Fig. 1G. Kruskal-Wallis test with Dunn's multiple comparisons test was used to compare all groups to inhibition by PD1. (** p<0.01; ns, not significant)

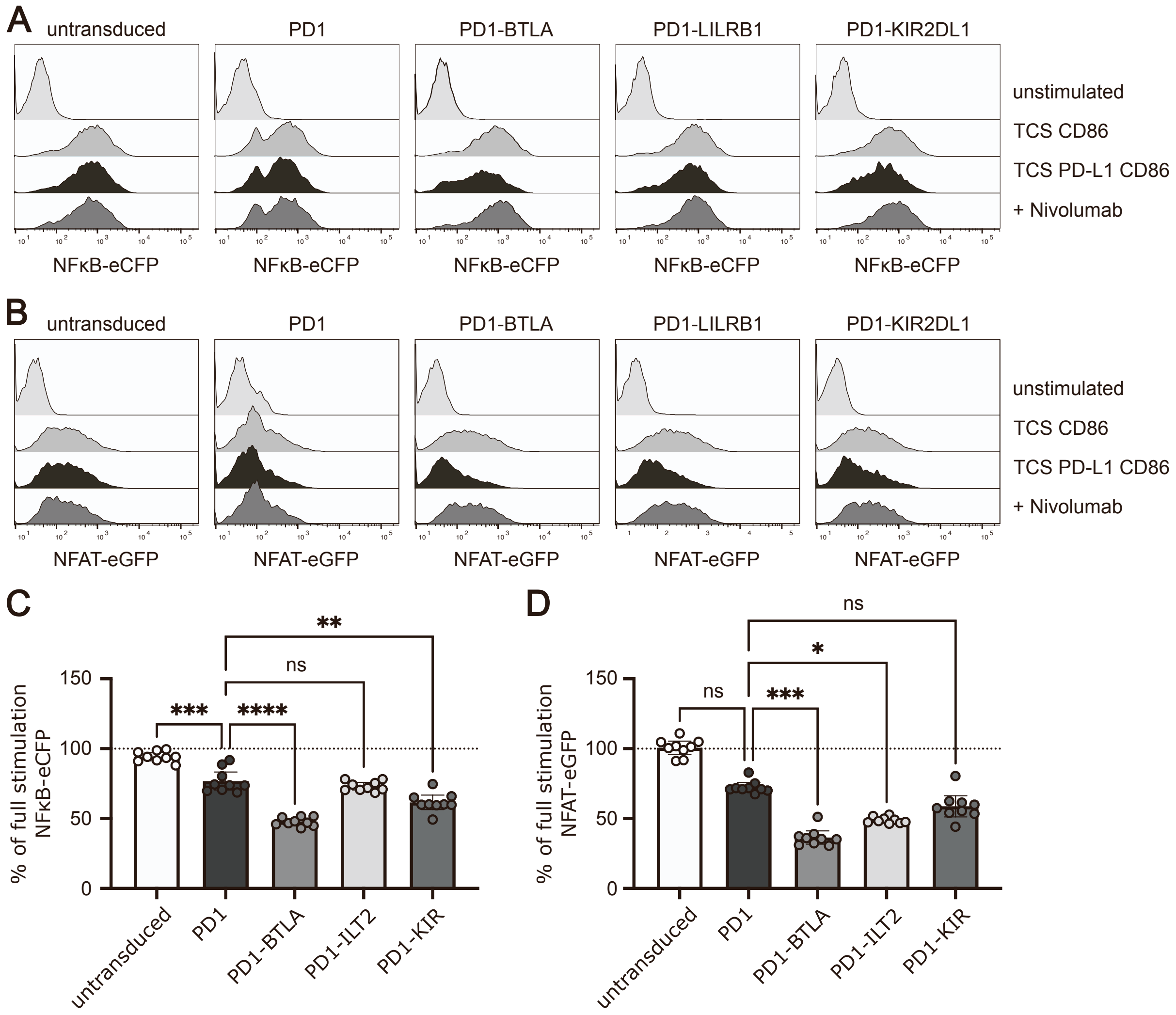

Fig. S2 **Influence of inhibitory signaling on costimulation via the CD28/CD86-pathway** TPR expressing the indicated PD1-chimera were cocultured with TCS CD86 or TCS PD-L1 CD86. After 24h, reporter gene expression was assessed by flow cytometry. **A,B** Representative histograms showing reporter gene fluorescence intensity of the indicated cell lines under different coculture conditions. (**A** NFκB-eCFP, **B** NFAT-eGFP). **C,D** Analysis of pooled data (n=3, each assay in triplicates). Stimulation in presence of TCS PD-L1 CD86 is expressed as percentage of stimulation by TCS CD86. Data is presented as mean with 95% CI. Dots represent individual repeats. **C** Percentage of full stimulation of the NFκB-eCFP reporter gene. Brown-Forsythe and Welch ANOVA tests with Dunnett's T3 multiple comparisons test was used to compare all groups to inhibition by PD1. **D** Percentage of full stimulation of the NFκB-eCFP reporter gene. Kruskal-Wallis test with Dunn's multiple comparisons test was used to compare all groups to inhibition by PD1. (* $p<0.05$; ** $p<0.01$; *** $p<0.001$; **** $p<0.0001$; ns, not significant)

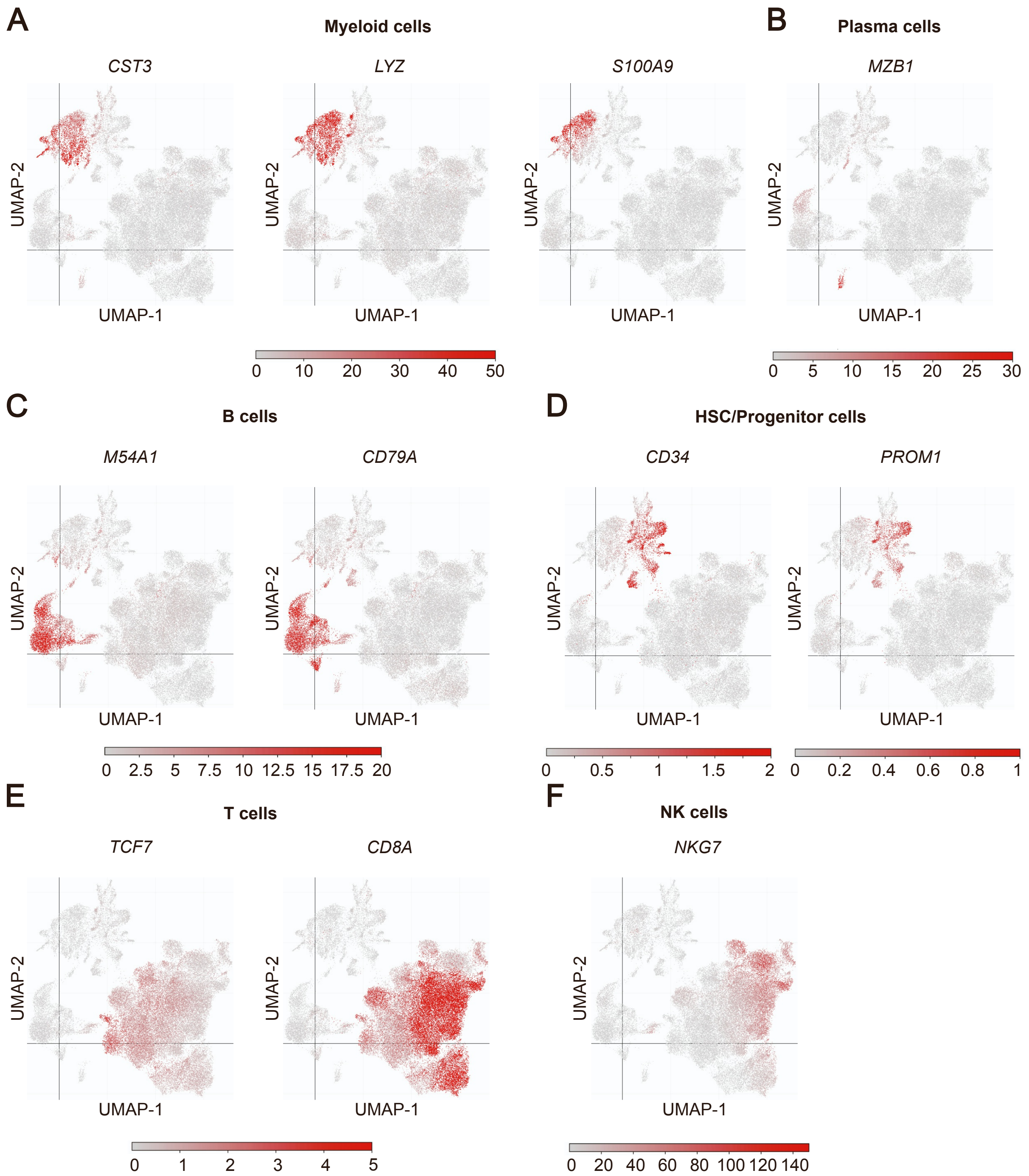

Fig. S3 **Identification of cell types in healthy control bone marrow samples.**

Publicly available, single cell level transcriptional data of bone marrow samples from healthy donors (n=10) was reanalyzed for cell type specific gene expression. UMAP projections of these cells are shown and expression of specific marker genes is highlighted in red **A** Myeloid cells were defined by high expression of *CST3*, *LYZ* and *S100A9,* **B** Plasma cells were defined by high expression of *MZB1,* **C** B cells were defined by high expression of *M54A1* and *CD79A,* **D** HSC/Progenitor cells were defined by high expression of *CD34* and *PROM1,* **E** T cells were defined by high expression of *TCF7* and *CD8A* **F** NK cells were defined by high expression of *NKG7.*

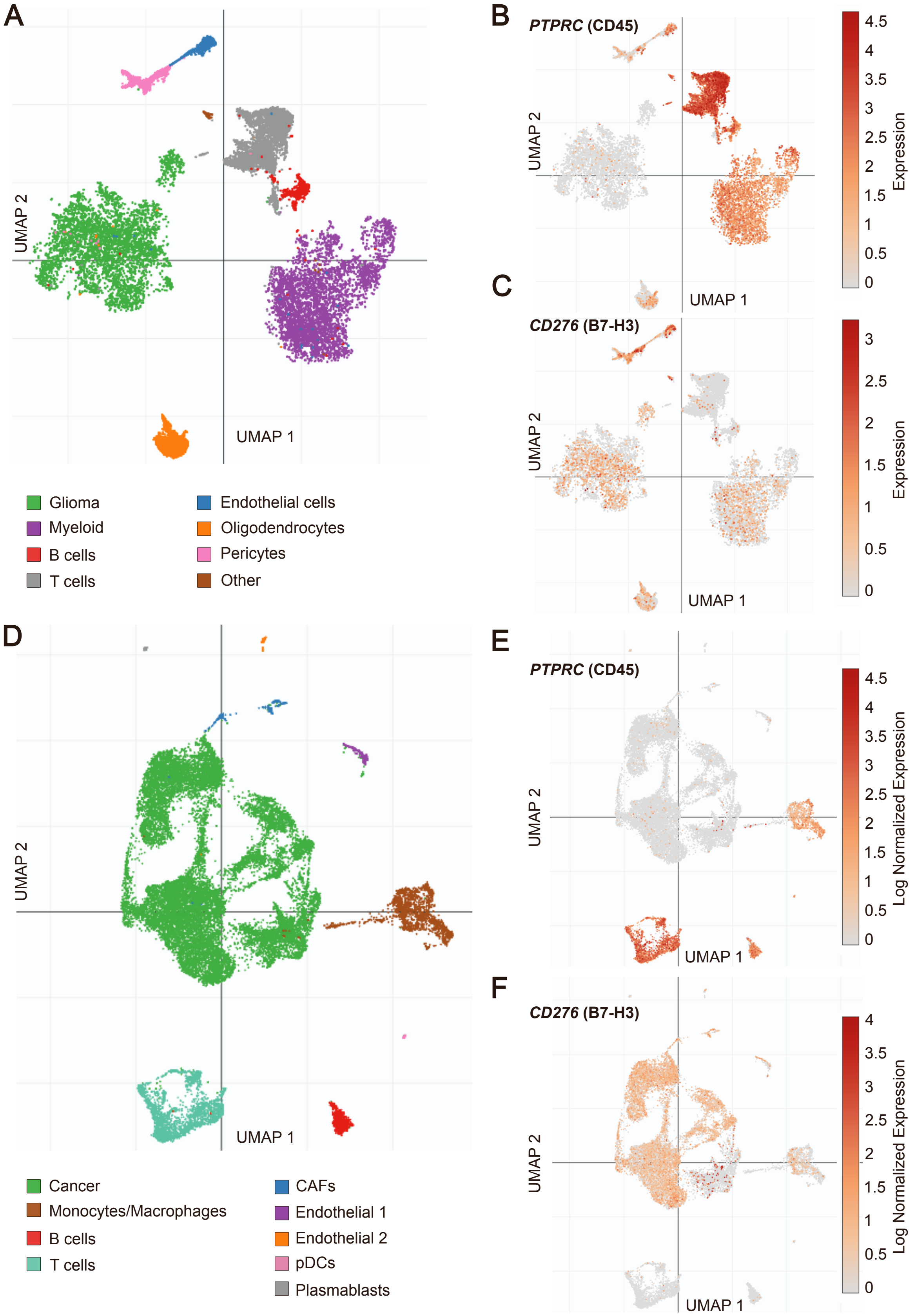
A
UMAP 2
UMAP 1
Glioma
Myeloid
B cells
T cells
Endothelial cells
Oligodendrocytes
Pericytes
Other
B
PTPRC (CD45)
UMAP 2
UMAP 1
Expression
C
CD276 (B7-H3)
UMAP 2
UMAP 1
Expression
D
UMAP 2
UMAP 1
Cancer
Monocytes/Macrophages
B cells
T cells
CAFs
Endothelial 1
Endothelial 2
pDCs
Plasmablasts
E
PTPRC (CD45)
UMAP 2
UMAP 1
Log Normalized Expression
F
CD276 (B7-H3)
UMAP 2
UMAP 1
Log Normalized Expression

Fig. S4 **Expression pattern of *PTPRC* (CD45) and *CD276* (B7-H3) in primary human tumor samples.** Publicly available, single cell level transcriptional data of glioma patients (n=18, 201.986 cells total) and a melanoma patient (n=1, 21,363 cells) were analyzed using the Single Cell Portal online-tool. **A** UMAP projection showing cell clustering and cell type annotation in glioma samples as defined in the metadata to this dataset (subsampling of 20.000 cells).[64] **B/C** UMAP projections indicating expression pattern of *PTPRC* (CD45) (**B**) and *CD276* (B7-H3) (**C**) **D** UMAP projection showing the cell type annotation in a melanoma patient as defined in the metadata to this dataset.[65] **E/F** UMAP projections indicating expression pattern of *PTPRC* (CD45) (**E**) and *CD276* (B7-H3) (**F**)

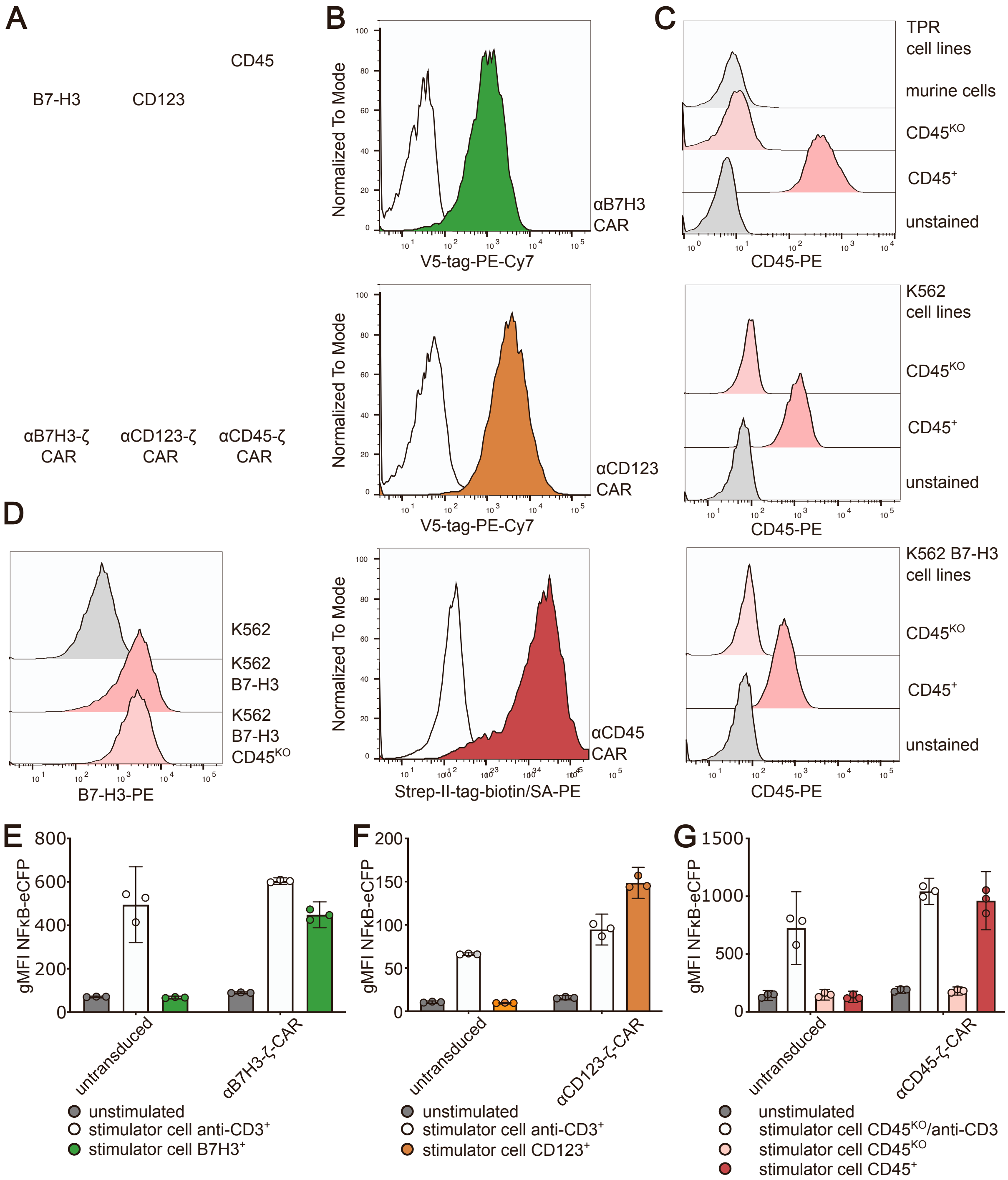

Fig. S5 **Creation of functional CARs from antibody sequences.**

A Scheme depicting assay setup to test functionality of the αB7H3-, αCD123- and αCD45-CARs. **B** Expression of the indicated CAR molecules on TPR cells determined by flow cytometry. Untransduced TPR cells served as negative control (open histograms) **C** To avoid interference with naturally expressed CD45 on TPR and K562 cells, CRISPR/Cas9 mediated knock out of endogenous CD45 was performed. Histograms show expression of CD45 on the treated (light red) and untreated (dark red) cell lines (TPR, K562, K562 B7-H3). Unstained cells (dark grey) and murine cells (light grey) not expressing human CD45 served as negative controls. **D** Expression of B7-H3 on K562 target cell lines analyzed by flow cytometry. **E/F/G** Untransduced TPR or TPR transduced with αB7H3- ζ (**E**), αCD123- ζ (**F**) or αCD45-ζ (**G**) CARs were cocultured with the, indicated K562 stimulator cells (n=1 each, each assay performed in triplicates). Data is presented as individual values with mean and 95% CI.

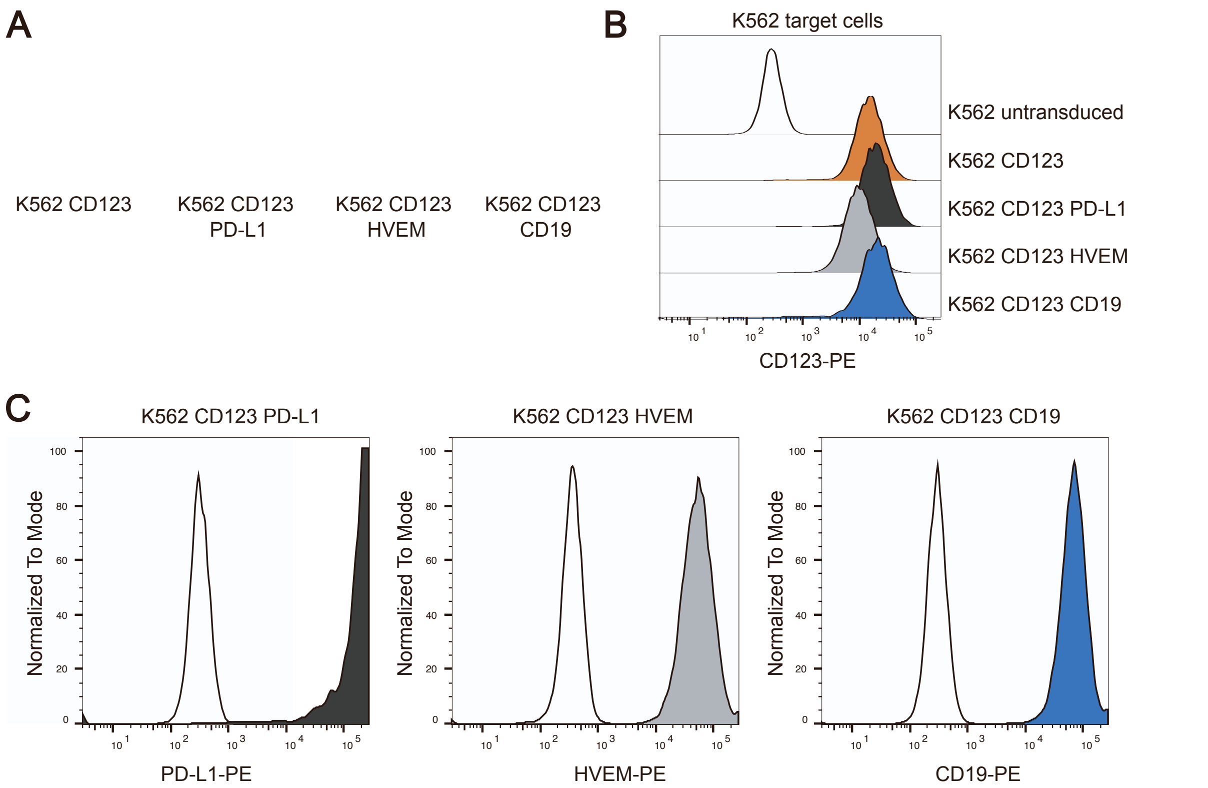

Fig. S6 **Creation and profiling of CD123 expressing K562 target cells. A** Scheme of K562 target cells that expressed CD123 alone or together with PD-L1, HVEM or CD19. **B** Expression of CD123 on all target K562 target cell lines as assessed by flow cytometry. Untransduced K562 cells served as negative control. **C** Panels show expression of inhibitory ligands PD-L1 (left, dark grey), HVEM (middle, light grey) and CD19 (right, blue) on the respective target cells as assessed by flow cytometry. Untransduced K562 served as negative controls.

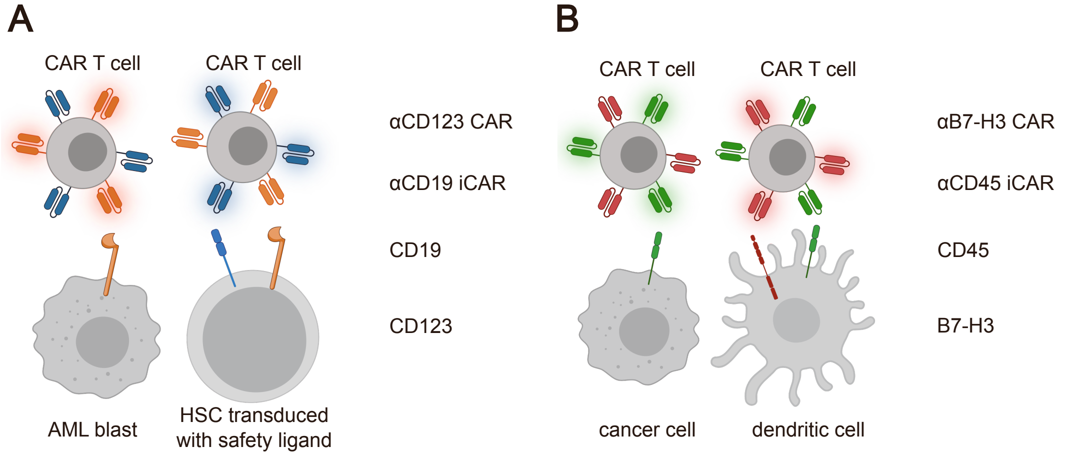

Fig. S7 **Potential CAR/iCAR antigen combinations to address relevant on-target/off-tumor toxicities A** Scheme outlining the αCD123-CAR/αCD19-iCAR strategy. CD123 is expressed on both, AML and HSCs. Transduced of HSCs expressing CD19 as “safety ligand” could avoid CAR/iCAR-T cell mediated toxicity. **B** Scheme outlining the αB7H3-CAR/αCD45-iCAR strategy. B7-H3 is expressed on many cancer entities and certain hematopoietic cell types, including dendritic cells. An αCD45-iCAR could prevent CAR/iCAR-T cell activation against hematopoietic cells.

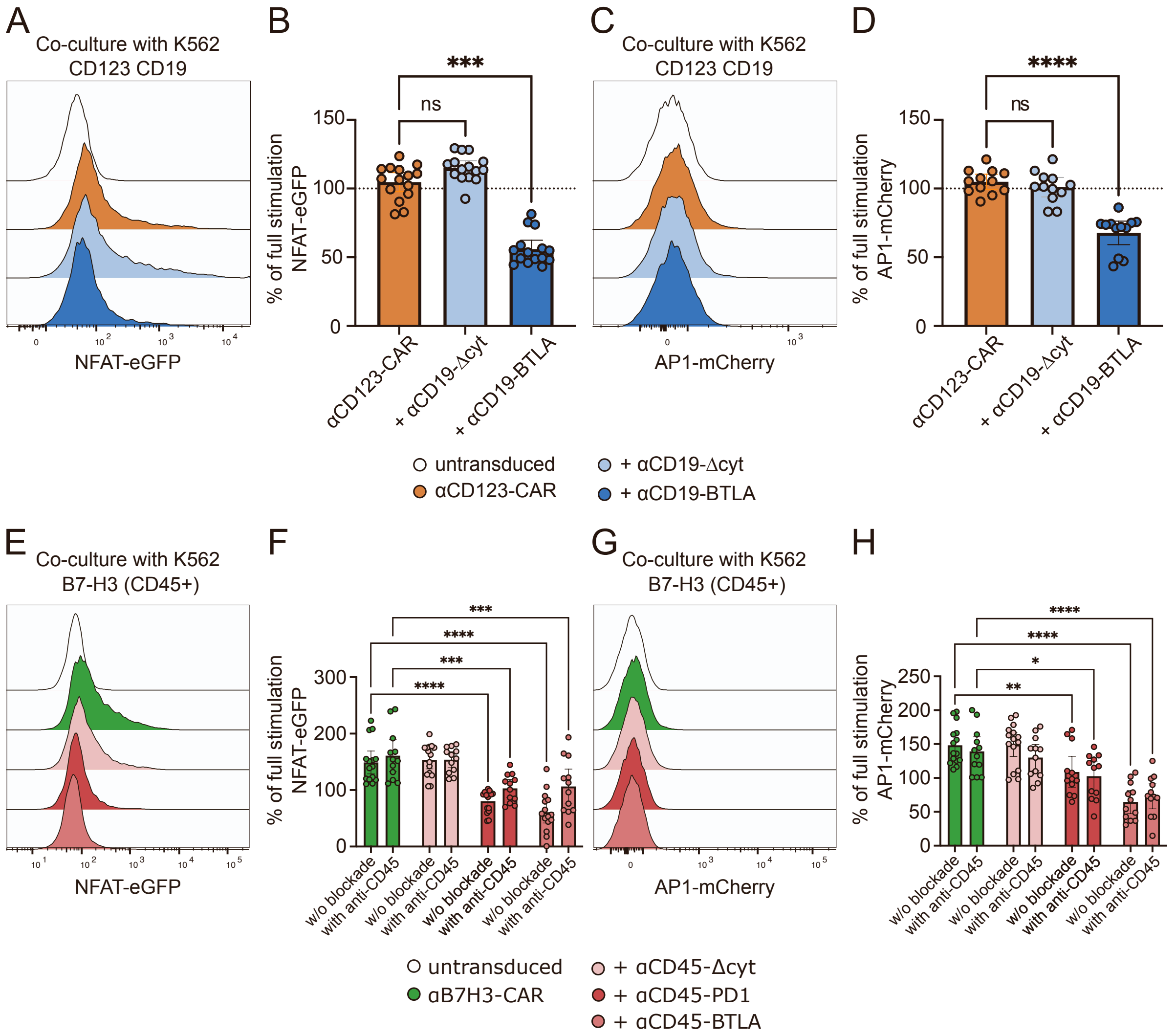

Fig. S8 **Effect of iCARs on NFAT-eGFP and AP1-mCherry reporter gene transcription A-D** Data on NFAT-eGFP and AP1-mCherry reporter gene transcription corresponding to the experiment shown in Fig. 3B,C. **A** Representative histograms showing NFAT-eGFP fluorescence intensity of the indicated TPR cell lines in coculture with K562 CD123 CD19. **B** Pooled data (n=5, each assay in triplicates) presented as mean with 95% CI. Dots represent individual repeats. **C,D** Data on AP1-mCherry reporter gene presented analogous to **A,B**, respectively. **B,D** Kruskal-Wallis test with Dunn's multiple comparisons test was performed to compare groups with αCD19-iCARs to stimulation by αCD123-ζ CAR alone. **E-H** Data on NFAT-eGFP and AP1-mCherry reporter gene transcription corresponding to the experiment shown in Fig. 3E,F. **E** Representative histograms showing NFAT-eGFP fluorescence intensity of the indicated TPR cell lines in coculture with K562 B7-H3 (CD45+). **F** Pooled data (n=5/ n=4 with anti-CD45-antibody, each assay in triplicates) presented as mean with 95% CI. Dots represent individual repeats. **G,H** Data on AP1-mCherry reporter gene presented analogous to **E,F**, respectively. **F,H** 2way-ANOVA with Dunnett's multiple comparisons test was performed to compare all groups to stimulation by αB7H3-ζ CAR alone. (* $p<0.05$; ** $p<0.01$; *** $p<0.001$; **** $p<0.0001$; ns, not significant)

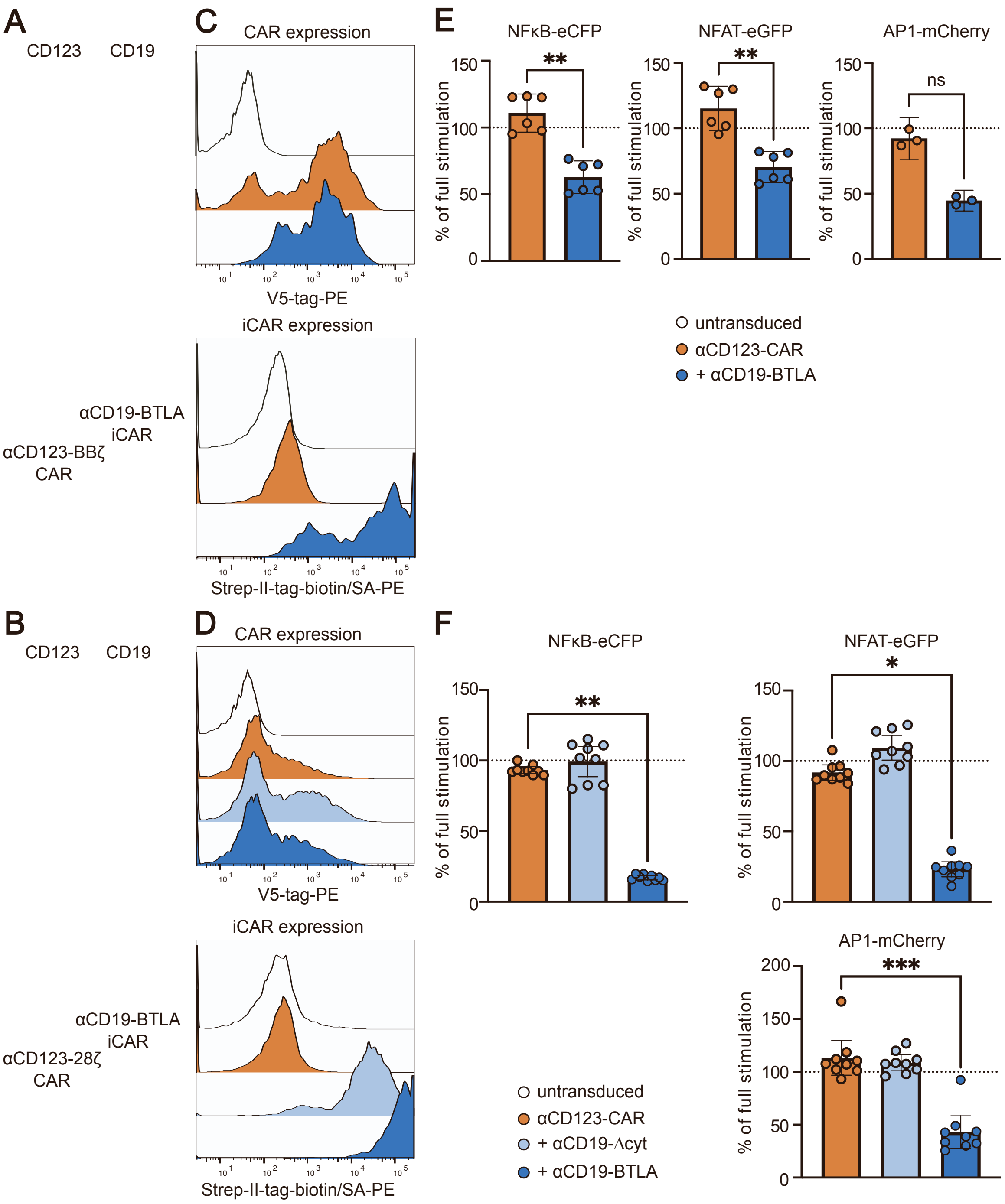
A
CD123
CD19
αCD19-BTLA
iCAR
αCD123-BBζ
CAR
B
CD123
CD19
αCD19-BTLA
iCAR
αCD123-28ζ
CAR
C
CAR expression
V5-tag-PE
iCAR expression
Strep-II-tag-biotin/SA-PE
D
CAR expression
V5-tag-PE
iCAR expression
Strep-II-tag-biotin/SA-PE
E
NFκB-eCFP
NFAT-eGFP
AP1-mCherry
% of full stimulation
**
**
ns
untransduced
αCD123-CAR
+ αCD19-BTLA
F
NFκB-eCFP
NFAT-eGFP
AP1-mCherry
% of full stimulation
**
*
***
untransduced
αCD123-CAR
+ αCD19-Δcyt
+ αCD19-BTLA

Fig. S9 **αCD19-BTLA iCAR inhibits second generation αCD123-BBζ and αCD123-28ζ CARs.**
A,B Schemes depicting assay setup to test capability of the αCD19-BTLA iCAR to suppress TPR activation by a second-generation αCD123 CARs (**A** αCD123-BBζ, **B** αCD123-28ζ). **C,D** Expression of αCD123-BBζ (**C**) and αCD123-28ζ (**D**) CAR and αCD19-BTLA iCAR (**C**) or αCD19-BTLA iCAR and αCD19-Δcyt CAR (**D**) on TPR cells. Untransduced TPR cells served as negative control. **E** TPR αCD123-BBζ and TPR αCD123-BBζ αCD19-BTLA cells were cocultured with K562 CD123 and K562 CD123 CD19. Reporter gene fluorescence intensity (NFκB-eCFP, NFAT-eGFP, AP-1-mCherry) induced by target cells coexpressing CD123 and CD19 is shown normalized to activation induced by target cells expressing CD123 only (n=2, each assay performed in triplicates). Mann-Whitney-test was performed to identify differences between groups. **F** TPR αCD123-28ζ without or with additional expression of αCD19-Δcyt or αCD19-BTLA were cocultured with K562 CD123 and K562 CD123 CD19. Reporter gene fluorescence intensity (NFκB-eCFP, NFAT-eGFP, AP-1-mCherry) induced by target cells coexpressing CD123 and CD19 is shown normalized to activation induced by target cells expressing CD123 only (n=3, each assay performed in triplicates). Kruskal-Wallis test with Dunn's multiple comparison test were performed to compare groups with αCD19-iCARs to stimulation by αCD123-ζ CAR alone. (** $p<0.01$; **** $p<0.0001$; ns, not significant not shown)

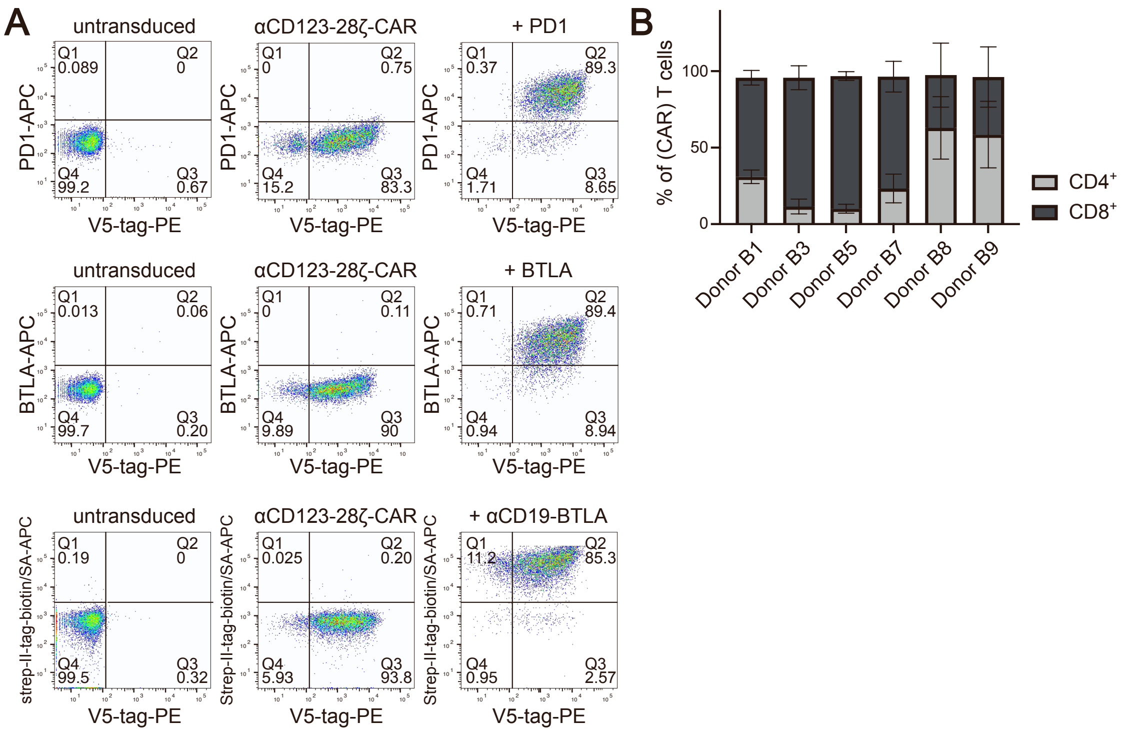

Fig. S10 **CAR T cell production.**

A Exemplary staining of untransduced T cells (left column) and CAR-T cells expressing αCD123-28ζ CAR alone (middle column) or together with inhibitory receptors (right column, PD1 (top row), BTLA (middle row) or αCD19-BTLA (bottom row)) of one donor. **B** Percentage of $CD4^+$ and $CD8^+$ T cells for each of the 6 donors. Mean percentage (95% CI) of $CD4^+$ and $CD8^+$ over all (CAR-)T cells (untransduced, αCD123-28ζ, αCD123-28ζ PD1, αCD123-28ζ BTLA and αCD123-28ζ αCD19-BTLA) derived from each donor are shown.

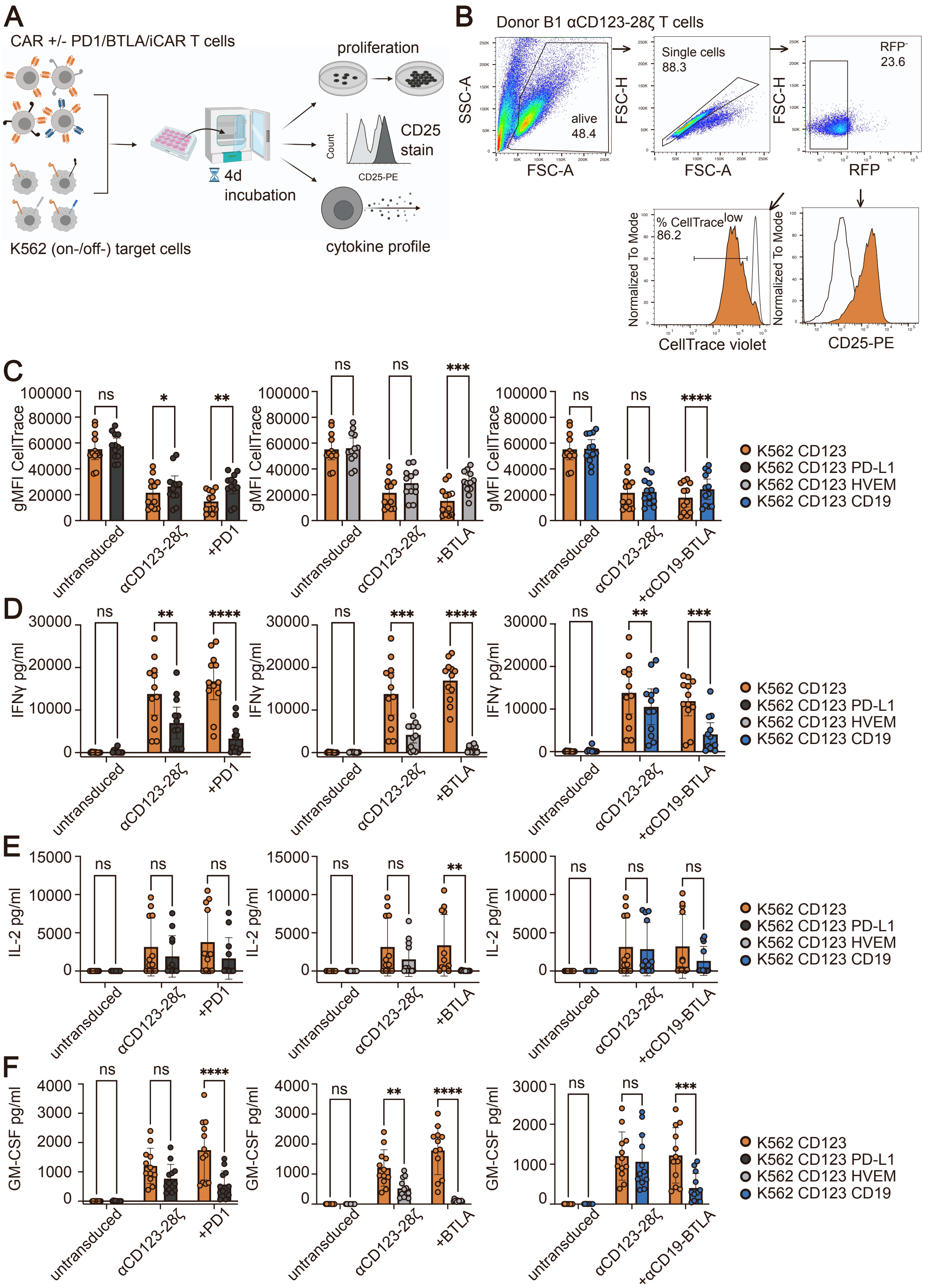

A
CAR +/- PD1/BTLA/iCAR T cells
proliferation
CD25 stain
4d incubation
cytokine profile
K562 (on-/off-) target cells
B
Donor B1 αCD123-28ζ T cells
alive 48.4
Single cells 88.3
RFP- 23.6
% CellTrace low 86.2
CellTrace violet
CD25-PE
C
gMFI CellTrace
D
IFNγ pg/ml
E
IL-2 pg/ml
F
GM-CSF pg/ml
untransduced
αCD123-28ζ
+PD1
+BTLA
+αCD19-BTLA
K562 CD123
K562 CD123 PD-L1
K562 CD123 HVEM
K562 CD123 CD19

Fig. S11 **Gating strategy for proliferation assay, CD25-stain and cytokine profiling of αCD123-CAR T cells. A** Scheme depicting assay set up to measure T cell effector functions (proliferation, upregulation of CD25, cytokine release). **B** Representative gating of Donor B1 αCD123-28ζ T cells to assess proliferation (CellTrace violet dilution assay) and CD25 upregulation. Live, single cells were identified by FSC-A/SSC-A, FSC-A/FSC-H gating and T cells (RFP^-) were analyzed as indicated. **C** Geometric mean fluorescent intensity of CellTrace violet in the proliferation assay. Data is presented analogous to Fig. 4B (percentage of $CellTrace^{low}$ T cells). Repeated measure two-way ANOVA with Šídák's multiple comparisons test was applied to compare proliferation of the indicated T cells in coculture with either K562 CD123 or with K562 CD123 expressing one of the inhibitory ligands (PD-L1, HVEM, CD19). **D/E/F** Analysis of further cytokines (**D** IFNγ, **E** IL-2, **F** GM-CSF analogous to Fig. 4C (TNFα). (*p<0.05; ** p<0.01; *** p<0.001; **** p<0.0001; ns, not significant)

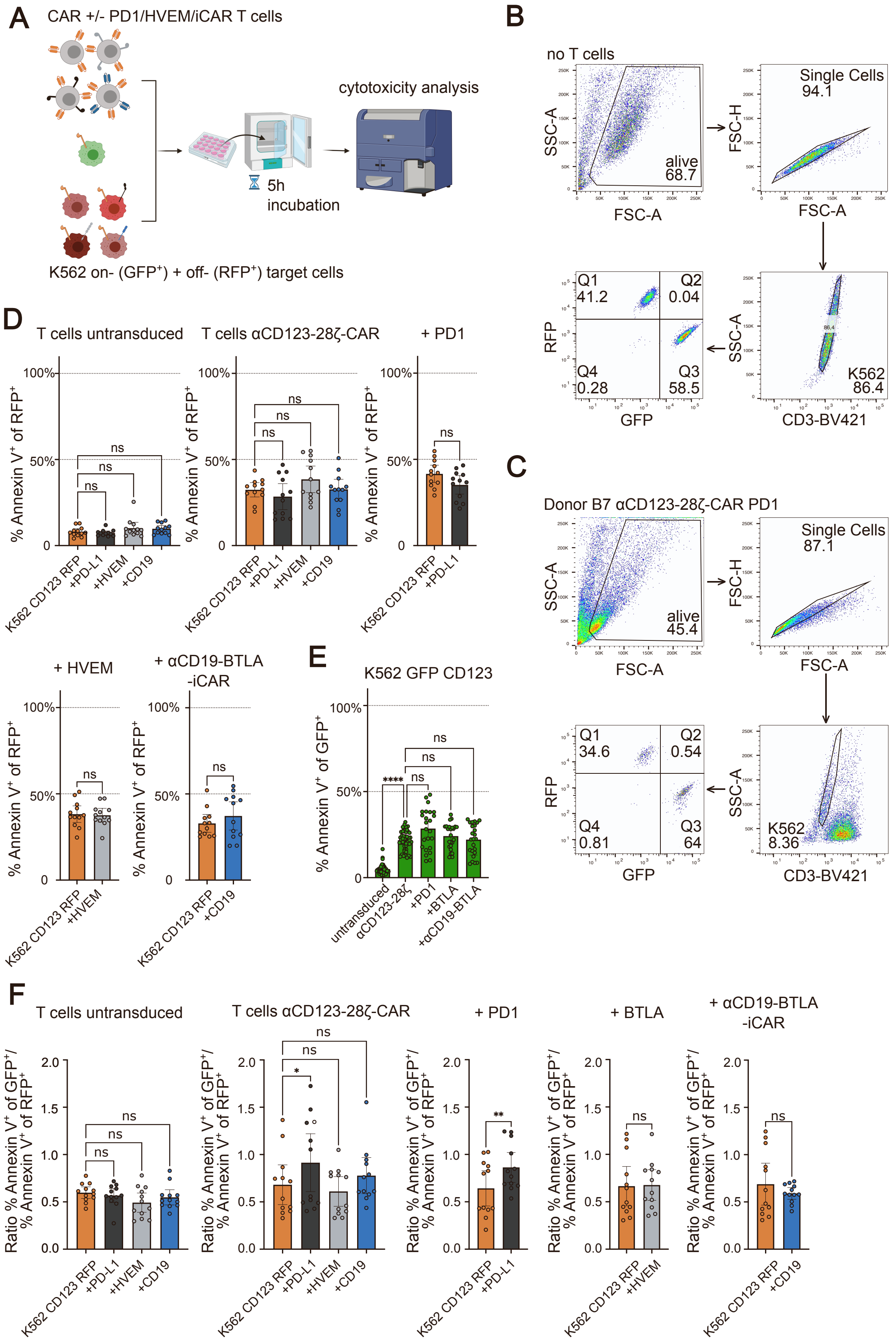
A
CAR +/- PD1/HVEM/iCAR T cells
cytotoxicity analysis
5h
incubation
K562 on- (GFP+) + off- (RFP+) target cells
B
no T cells
SSC-A
FSC-A
alive
68.7
FSC-H
Single Cells
94.1
RFP
GFP
Q1
41.2
Q2
0.04
Q4
0.28
Q3
58.5
SSC-A
CD3-BV421
K562
86.4
C
Donor B7 αCD123-28ζ-CAR PD1
alive
45.4
Single Cells
87.1
Q1
34.6
Q2
0.54
Q4
0.81
Q3
64
K562
8.36
D
T cells untransduced
T cells αCD123-28ζ-CAR
+ PD1
+ HVEM
+ αCD19-BTLA -iCAR
% Annexin V+ of RFP+
100%
50%
0
ns
K562 CD123 RFP
+PD-L1
+HVEM
+CD19
E
K562 GFP CD123
% Annexin V+ of GFP+
****
untransduced
αCD123-28ζ
+PD1
+BTLA
+αCD19-BTLA
F
T cells untransduced
T cells αCD123-28ζ-CAR
+ PD1
+ BTLA
+ αCD19-BTLA -iCAR
Ratio % Annexin V+ of GFP+/ % Annexin V+ of RFP+
2.0
1.5
1.0
0.5
0.0
*
**
ns

Fig. S12 **Assessment of killing in a dual-target-cell killing assay. A** Scheme depicting the setup of CAR-T cell dual-target cell cytotoxicity assay. Untransduced T cells or αCD123-CAR-T cells without or with additional expression of PD1, BTLA or a αCD19-BTLA iCAR were cocultured with K562 GFP^+ CD123 target cells and K562 RFP^+ target cells expressing CD123 alone or together with PD-L1, HVEM or CD19 for 5h (E:T ratio of 10:1:1, n=6 donors, each assay in duplicates). Subsequently, cells were stained with Annexin-V-PE and apoptotic target cells were identified by flow cytometry. **B,C** Gating strategy for the flow cytometry based dual-target-cell cytotoxicity assay. (**B**) shows representative plots of the no T cell control, (**C**) shows representative plots of a condition with CAR-T cells. **D** Rate of Annexin-V^+/RFP^+ of total RFP^+ cells presented as mean with 95% CI. Dots represent individual repeats. Panels show data for untransduced, CAR-, CAR/PD1-, CAR/BTLA- and CAR/iCAR-transduced T cells separately. Bars indicate different RFP^+ target cell lines (K562 CD123 (orange), K562 CD123 PD-L1 (dark grey), K562 CD123 HVEM (light grey) and K562 CD123 CD19 (blue). Comparisons between different target cell lines were analyzed by Friedman-test with Dunn's multiple comparisons test (T cell untransduced, non-normally distributed), RM one-way ANOVA with Holm-Šidák's multiple comparisons test (T cells αCD123-28ζ) or paired, two-tailed t-test (T cells αCD123-28ζ PD1, T cells αCD123-28ζ BTLA, T cells αCD123-28ζ αCD19-BTLA). **E** Rate of Annexin-V^+ GFP^+ of total GFP^+ cells presented as mean with 95% CI. Dots represent individual repeats. Differences compared to killing by αCD123-28ζ T cells were analyzed by Kruskal-Wallis test with Dunn's multiple comparisons test. **F** Panels show ratio of Annexin-V^+ of GFP^+ cells to Annexin-V^+ of RFP^+ cells after coculture with the indicated T cells to detect differential killing of inhibitory ligand expressing target cells. RM one-way ANOVA test with Holm-Šidák's multiple comparisons test or paired, two-tailed t-test for normally distributed data (T cells untransduced, αCD123-ζ + PD1, αCD123-ζ + BTLA, αCD123-ζ + αCD19-BTLA) or Friedman-test with Dunn's multiple comparisons test for non-normally distributed data (T cells αCD123-ζ) was used. (* $p<0.05$; ** $p<0.01$; ns, not significant)

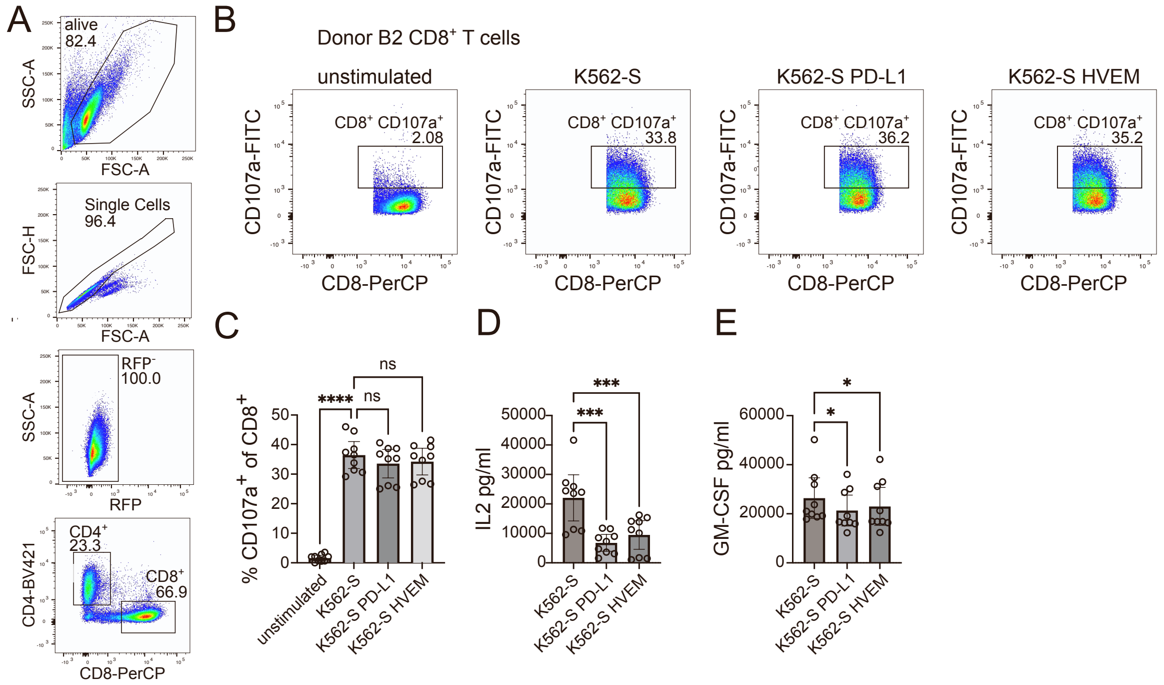

Fig. S13 **PD1 and BTLA engagement does not suppress degranulation of CD8⁺ T cells A** Gating strategy to identify CD8⁺ T cells in coculture with K562-S, K562-S PD-L1 or K562-S HVEM target cells. **B,C** T cells were cocultured with indicated target cells for 5h in presence of CD107a-FITC antibody and Golgistop/Golgiplug reagents. (**B**) Representative plots show CD107a staining of CD8⁺ T cells of one donor. (**C**) Percentage of CD107a⁺ cells of CD8⁺ T cells (n=3, performed in triplicates) **D-E** Parallel cocultures were performed for 24-48h (n=3, performed in triplicates). Then, supernatants were analyzed for cytokine concentration. Bar graphs indicate concentration of IL-2 (**D**) and GM-CSF (**E**) in culture supernatants. For statistical analysis one-way RM ANOVA with Šidák's multiple comparisons test with donor-specific matching was performed. (* $p<0.05$; *** $p<0.001$; **** $p< 0.0001$; ns, not significant)

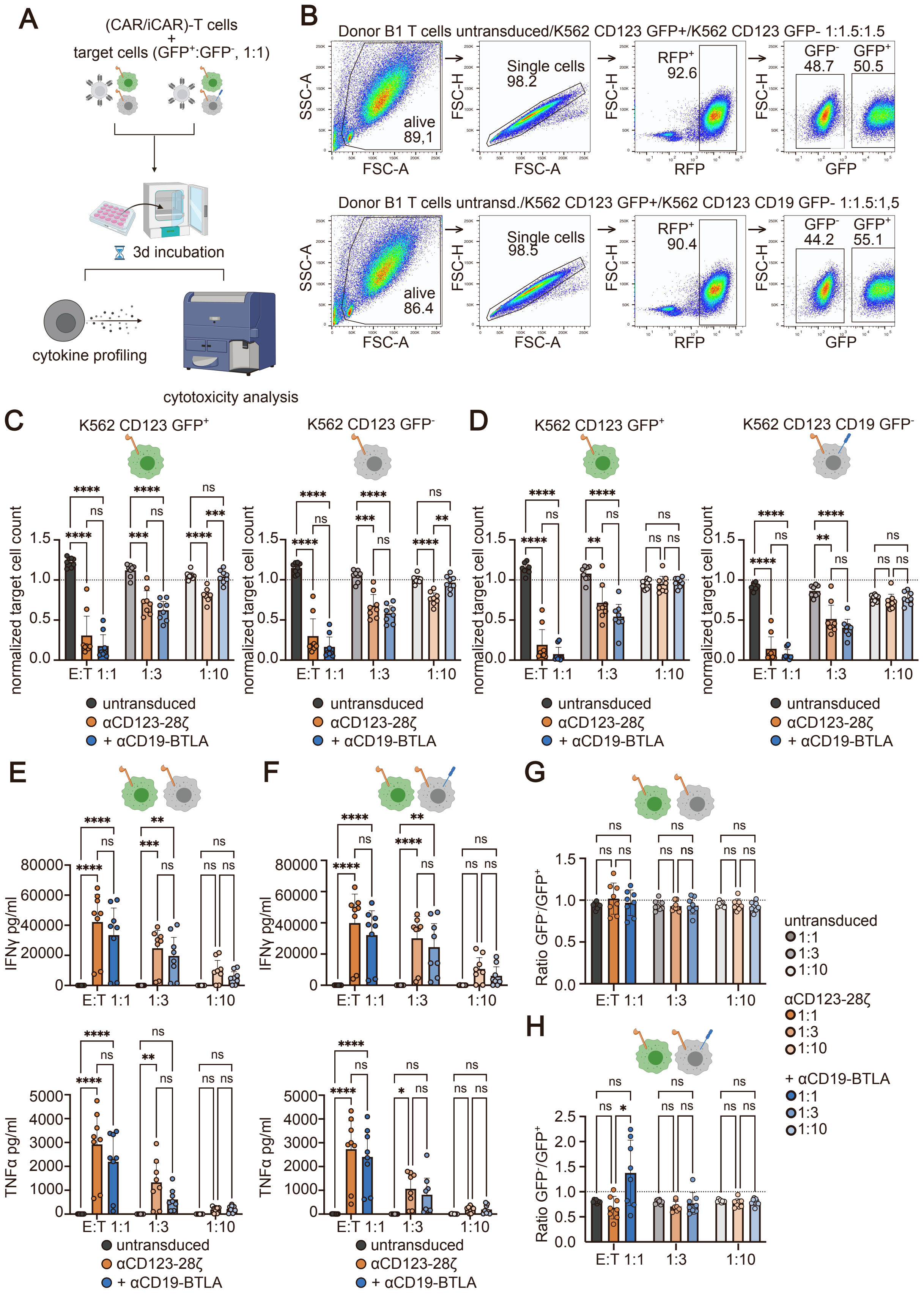
A
(CAR/iCAR)-T cells + target cells (GFP+:GFP-, 1:1)
3d incubation
cytokine profiling
cytotoxicity analysis
B
Donor B1 T cells untransduced/K562 CD123 GFP+/K562 CD123 GFP- 1:1.5:1.5
alive 89,1
Single cells 98.2
RFP+ 92.6
GFP- 48.7
GFP+ 50.5
Donor B1 T cells untransd./K562 CD123 GFP+/K562 CD123 CD19 GFP- 1:1.5:1,5
alive 86.4
Single cells 98.5
RFP+ 90.4
GFP- 44.2
GFP+ 55.1
SSC-A
FSC-A
FSC-H
RFP
GFP
C
K562 CD123 GFP+
K562 CD123 GFP-
D
K562 CD123 GFP+
K562 CD123 CD19 GFP-
normalized target cell count
E:T 1:1
1:3
1:10
untransduced
αCD123-28ζ
+ αCD19-BTLA
E
F
G
H
IFNγ pg/ml
TNFα pg/ml
Ratio GFP-/GFP+
untransduced
1:1
1:3
1:10
αCD123-28ζ
1:1
1:3
1:10
+ αCD19-BTLA
1:1
1:3
1:10

Fig. S14 **Cytotoxic activity and cytokine production in cocultures containing target cells expressing CD123 only as well as target cells coexpressing CD123 and CD19. A** Scheme depicting assay setup. Untransduced T cells or αCD123-28ζ CAR-T cells with or without αCD19-BTLA were cocultured for 72h with GFP^+ K562 CD123 and either GFP^- K562 CD123 or GFP^- K562 CD123 CD19 cells. Subsequently, cytotoxicity and cytokine concentrations were assessed (n=4 donors, all conditions in duplicates). **B** Gating strategy to assess target cell counts in coculture. **C,D** Target cell counts were normalized to target cell count without T cells. Panels separately show the counts of the two indicated target cell populations in cocultures containing no target cells expressing CD19 (**C**) and of the two indicated target cell populations in cocultures containing target cells expressing CD19 (**D**). **E,F** Concentration of IFNγ (top panels) and TNFα (bottom panels) in the cocultures containing no target cells expressing CD19 (**E**) and in cocultures containing target cells expressing CD19 (**F**). **G,H** Ratio of normalized counts of GFP^+ and GFP^- target cells in cocultures with GFP^- target cells not expressing CD19 (**G**) and GFP^- target cells expressing CD19 as indicated (**H**). For statistical analysis two-way RM ANOVA with Tukey's multiple comparisons test with donor-specific matching was performed. (* p<0.05; ** p<0.01; *** p<0.001 ; **** p< 0.0001; ns, not significant)
